# Vitamin D and Vitamin B_12_ in Psychiatric Disorders: An Exploratory Systematic Review and Meta-Analysis of Nutrient-Specific Status and Supplementation Evidence

**DOI:** 10.3390/diseases14050167

**Published:** 2026-05-10

**Authors:** Lavinia-Alexandra Moroianu, Cecilia Curis, Valeriu Ardeleanu, Roxana Elena Bogdan-Goroftei, Simona-Dana Mitincu-Caramfil, Marius Moroianu, Alina Pleșea-Condratovici

**Affiliations:** 1 Faculty of Medicine and Pharmacy, Research Centre in the Medical-Pharmaceutical Field, Dunarea de Jos University of Galați, 800008 Galati, Romania; lavinia.moroianu@yahoo.com (L.-A.M.); cecilia_curis@yahoo.com (C.C.); simona.mitincu@ugal.ro (S.-D.M.-C.); moroianu.g.marius@gmail.com (M.M.); alina.plesea@ugal.ro (A.P.-C.); 2 Faculty of Medicine, “Ovidius” University of Constanta, Mamaia Boulevard 124, 900527 Constanta, Romania

**Keywords:** vitamin D, vitamin B_12_, nutritional psychiatry, systematic review, exploratory meta-analysis, nutrient status, supplementation, psychiatric disorders, heterogeneity

## Abstract

Background/Objectives: Evidence linking vitamins D and B_12_ to psychiatric outcomes remains heterogeneous across designs, populations, phenotypes, exposures, and outcome formats. Methods: We conducted a PRISMA 2020 systematic review and exploratory meta-analysis of nutrient-specific status and supplementation evidence. PubMed/MEDLINE, APA PsycInfo, Cochrane Library, Google Scholar, ClinicalTrials.gov, and ProQuest were searched for human studies published in 2016–2025, with a final update on 1 March 2026. Forty-six studies were included (24 randomized controlled trials, 22 observational studies; *N* = 69,902), and 44 contributed quantitative data. Effects were harmonized to odds ratios (ORs) for cross-family comparability and pooled using Hartung–Knapp random-effects models; supplementation evidence was additionally interpreted on the standardized mean difference (SMD) scale. Results: Across the main evidence families, pooled estimates showed substantial heterogeneity and limited generalizability. Vitamin D supplementation showed an initial inverse estimate on the secondary harmonized OR scale (OR = 0.439, 95% CI 0.272–0.710) and a clinically interpretable SMD of −0.454 (95% CI −0.718 to −0.189), but heterogeneity was high (*I*^2^ = 84.2%) and trim-and-fill attenuated the OR estimate to the null (OR = 0.88, 95% CI 0.48–1.63). Vitamin D status showed a similar pattern (primary OR = 0.615, 95% CI 0.424–0.890; trim-and-fill OR = 0.90, 95% CI 0.54–1.49). Vitamin B_12_ status was inversely associated with outcomes (OR = 0.310, 95% CI 0.115–0.834), but heterogeneity was extreme (*I*^2^ = 94.8%). B_12_ supplementation evidence was sparse and null. Conclusions: The evidence supports targeted deficiency assessment, not routine supplementation.

## 1. Introduction

### 1.1. Clinical and Biological Rationale for an Exploratory Nutrient-Evidence Map of Vitamins D and B_12_ in Psychiatric Disorders

Vitamin B_12_ (cobalamin) is clinically relevant to psychiatric practice because deficiency can manifest through nonspecific yet potentially reversible affective, cognitive, and behavioral symptoms, sometimes preceding overt hematologic abnormalities at first clinical contact. This creates a practical risk of misattribution to primary psychopathology or apparent treatment resistance when B_12_-related etiologies are not considered. Interpretation is also biomarker-dependent: serum B_12_ alone may misclassify status, whereas symptom-compatible or borderline cases often require contextual interpretation with functional indices such as methylmalonic acid and/or homocysteine. These clinical realities justify separating B_12_ status-outcome associations from B_12_ supplementation effects, because treatment responsiveness is expected to vary according to baseline status, population characteristics, comorbidity burden, and psychiatric outcome definition [1,2,3,4,5,6,7].

The expression “nutrient-specific evidence streams” denotes analytically distinct bodies of evidence for vitamin D and vitamin B_12_, further separated by comparison type: status-outcome associations versus supplementation-placebo/usual-care comparisons. This terminology avoids implying patient-level dual-biomarker stratification or within-study vitamin D × vitamin B_12_ interaction testing, which was not consistently available in the primary literature. A harmonized effect-size framework can support structured evidence mapping and cross-family comparability; however, it does not imply biological or clinical equivalence across nutrients, randomized and observational designs, age strata, psychiatric phenotypes, exposure definitions, or outcome formats.

One-carbon metabolism (OCM) integrates folate and methionine cycles with transsulfuration to sustain methyl-group transfer, redox homeostasis, and nucleotide biosynthesis within the central nervous system. Vitamin B_12_ is essential for methionine synthase-mediated remethylation, supporting S-adenosylmethionine (SAM) availability and limiting homocysteine accumulation; disruption of this pathway may promote a low-methylation, pro-oxidative biological state with downstream epigenetic and neurochemical consequences. OCM also intersects monoaminergic biology through redox-sensitive bottlenecks, including tetrahydrobiopterin (BH4), a cofactor for tryptophan hydroxylase (TPH2) and tyrosine hydroxylase (TH), both of which may be functionally constrained by inflammation and oxidative stress. SAM-dependent methyltransferase processes may additionally contribute to transcriptional regulation of pathway-relevant genes. These mechanistic links support biological plausibility, but they do not establish uniform psychiatric effects of vitamin B_12_ status or supplementation across diagnostic categories. Meta-analytic and genetic signals involving homocysteine and OCM-relevant loci across mood and psychosis spectra suggest that nutrient status, methylation biology, oxidative stress, and inflammatory vulnerability may contribute to clinically meaningful heterogeneity in nutritional psychiatry [8,9,10,11,12,13,14,15]. Accordingly, B_12_-related evidence requires interpretation as contex-tdependent and potentially deficiency-linked, rather than as a single generalizable psychiatric effect.

Vitamin D is a pleiotropic secosteroid with central nervous system activity, supported by vitamin D receptor (VDR) expression in multiple brain regions and by local metabolism enabling 1,25-dihydroxyvitamin D (1,25(OH)2D) signaling within neural-glial compartments. Through genomic VDR-retinoid X receptor (RXR) actions at vitamin D response elements (VDREs), vitamin D can influence transcriptional programs relevant to neurodevelopment, synaptic plasticity, oxidative balance, stress responsivity, and immune regulation. These pathways provide plausible entry points for associations with mood, cognition, sleep, and neurodevelopmental phenotypes, particularly under conditions of baseline deficiency or elevated comorbidity burden. A translational hypothesis links vitamin D to serotonergic homeostasis. Experimental and mechanistic studies have reported associations between 1,25(OH)2D signaling and serotonin transporter (SERT; SLC6A4), monoamine oxidase A (MAO-A), and tryptophan hydroxylase-related pathways, suggesting plausible routes through which vitamin D status could interact with mood-relevant phenotypes [16,17,18,19,20,21,22]. Nevertheless, most clinical studies eligible for quantitative synthesis did not directly measure serotonergic biomarkers, dopaminergic biomarkers, or pathway activity. Consequently, serotonergic or dopaminergic subgrouping can only be interpreted as phenotype-informed and exploratory, not as direct evidence of monoaminergic mechanisms.

Convergent biology also supports evaluating nutrient-related psychiatric evidence within broader neuroimmune, astroglial, metabolic, and sleep-linked contexts, rather than within isolated nutrient silos. Neuroinflammation is increasingly regarded as a cross-diagnostic contributor to symptom dimensions spanning mood, cognition, sleep, and psychosis-related phenotypes. Astroglial and glymphatic mechanisms have also been proposed as interfaces linking sleep regulation, extracellular fluid clearance, inflammatory tone, and psychiatric vulnerability [23,24,25,26,27]. Within this broader framework, vitamin D may plausibly influence immune and neuroglial signaling, whereas the folate-B_12_-homocysteine axis may link methylation biology to oxidative and inflammatory vulnerability. These biological considerations justify targeted deficiency hypotheses and exploratory subgroup questions, but they do not establish that nutrient effects are generalizable across psychiatric diagnoses or that supplementation benefits unselected psychiatric populations. The relevant clinical question is therefore not whether vitamin D or vitamin B_12_ exerts a uniform psychiatric effect, but whether specific nutrient status contexts, population characteristics, and comorbidity patterns identify subgroups in which assessment or correction of deficiency may be clinically meaningful.

### 1.2. Objectives and Research Questions (Including Exploratory Subgroup Questions)

Despite extensive growth in nutritional psychiatry, evidence linking vitamin D and vitamin B_12_ to psychiatric outcomes remains methodologically fragmented. Primary studies operationalize exposures heterogeneously, including status thresholds, assay context, supplementation dose, supplementation duration, comparator definition, psychiatric phenotype, and outcome format. Status-outcome associations and supplementation-comparator effects also support different inferential claims: observational status studies can identify associations and risk markers, whereas randomized supplementation trials are required to estimate intervention effects. Combining these evidence types without explicit separation may obscure design-specific inference and overstate clinical generalizability. A further challenge concerns reproducibility and statistical comparability. Effect sizes, covariate adjustment sets, baseline deficiency enrichment, and outcome timepoints are often incompletely or inconsistently reported. Prior syntheses also vary in random-effects estimators, small-study assessments, sensitivity analyses, and moderator testing. These limitations support a prespecified exploratory evidence map and meta-analysis that standardizes effect metrics where appropriate, separates nutrient-specific evidence streams, distinguishes status-outcome associations from supplementation comparisons, and evaluates age- and phenotype-informed subgroup patterns as potential sources of heterogeneity rather than as confirmatory mechanistic tests.

The overarching aim of this study was to provide an exploratory, nutrient-specific systematic review and meta-analysis of evidence linking vitamin D and vitamin B_12_ to clinically relevant psychiatric outcomes. The evidence was organized into parallel streams according to nutrient and comparison type: vitamin D supplementation, vitamin D status, vitamin B_12_ status, and vitamin B_12_ supplementation. Within each eligible stream, pooled effects were estimated only when statistical synthesis was defensible and were interpreted alongside heterogeneity, prediction intervals, risk of bias, certainty of evidence, and sensitivity analyses.

The project was structured to: (i) map and characterize the evidence base across psychiatric phenotypes, exposure definitions, and study designs; (ii) quantify global pooled effects within prespecified evidence families when appropriate; and (iii) explore whether age strata and phenotype-informed subgroup categories were associated with differences in effect estimates. These subgroup analyses were designed to generate hypotheses about clinically meaningful heterogeneity, not to demonstrate serotonergic, dopaminergic, neuroimmune, astroglial, or metabolic mechanisms.

The central research question was: across psychiatric disorders, what are the direction, magnitude, robustness, and generalizability of associations between vitamin D or vitamin B_12_ exposures and psychiatric outcomes within nutrient- and comparison-specific evidence streams? A linked translational question was whether the combined evidence supports targeted assessment and correction of reversible deficiency in clinically selected contexts, rather than routine adjunctive supplementation across unselected psychiatric populations. Heterogeneity was treated as a central finding rather than as a purely statistical nuisance. Accordingly, subgroup and moderator analyses were used to explore whether study-level characteristics, age strata, phenotype-informed categories, baseline deficiency enrichment, and comorbidity burden could help explain variability in effect estimates. These analyses were not designed to prove pathway mechanisms because serotonergic, dopaminergic, astroglial, neuroimmune, or metabolic pathway biomarkers were not consistently measured across the included primary studies.

## 2. Materials and Methods

### 2.1. Reporting Standards and Study Design

This work was conducted as a systematic review with an exploratory, prespecified, multi-phase meta-analytic framework integrating evidence on vitamin D and vitamin B_12_ across psychiatric phenotypes. Reporting followed the PRISMA 2020 statement and its accompanying Explanation and Elaboration guidance [28,29]. The operational protocol—eligibility criteria, search architecture, selection procedures, data-extraction schema, coding rules, risk-of-bias appraisal strategy, and statistical analysis plan, including subgroup and sensitivity analyses—was defined before study screening and quantitative synthesis were initiated.

The review was not prospectively registered in PROSPERO or an equivalent systematic review registry. To mitigate this limitation and support transparency, protocol-equivalent documentation and a complete decision-tracking audit trail were maintained and are provided in the Supplementary Materials. These materials include full database-specific search strategies (Table S1), the included-study synopsis and extraction structure (Tables S2 and S4), full-text exclusions with explicit reasons (Table S3), methodological quality and risk-of-bias appraisals with certainty judgments (Tables S5 and S6), and analytic outputs supporting the multi-phase synthesis, SMD-based supplementation interpretation, and sparse exploratory vitamin B_12_ supplementation evidence (Tables S7–S16). The absence of prospective registration is also acknowledged as a limitation in the Discussion.

### 2.2. Systematic Review Methodology (PICOS; Search; Selection; Extraction)

Eligibility criteria were prespecified using the PICOS framework (Population, Intervention/Exposure, Comparator, Outcomes, Study design). Eligible reports were human primary studies published between 1 January 2016 and 31 December 2025 that evaluated either: (i) vitamin D and/or vitamin B_12_ status, including circulating 25-hydroxyvitamin D (25(OH)D) or serum vitamin B_12_, in relation to psychiatric diagnoses or validated psychiatric symptom outcomes; or (ii) vitamin D and/or vitamin B_12_ supplementation, alone or as a clearly specified regimen, compared with placebo, usual care, or an explicit comparator condition. Eligible populations comprised children/adolescents and adults with psychiatric disorders defined by DSM-5-TR (Diagnostic and Statistical Manual of Mental Disorders, Fifth Edition, Text Revision) [30] and ICD-11 (International Statistical Classification of Diseases and Related Health Problems, 11th Revision) [31], or clinically meaningful symptom states measured with validated instruments.

Animal and in vitro studies, narrative reviews, editorials, protocols without results, and secondary evidence syntheses without new primary patient-level data were excluded from the quantitative synthesis. Higher-level evidence syntheses were retained only for background, mechanistic context, or citation chasing when relevant, to avoid double counting. Reports lacking extractable effect estimates or sufficient data for conversion to a prespecified common metric were excluded from quantitative pooling but could be retained narratively when clinically informative. Eligibility decisions and reasons for exclusion are documented in Table S3 of the Supplementary Materials.

Information sources and database-specific search architectures are provided in Table S1 of the Supplementary Materials. Searches covered PubMed/MEDLINE, APA PsycInfo, Cochrane Library including CENTRAL, Google Scholar, ClinicalTrials.gov, and ProQuest, with coverage from 1 January 2016 to 31 December 2025 and a final search update on 1 March 2026. Search strategies combined controlled vocabulary where available and free-text terms for vitamin D, vitamin B_12_, psychiatric outcomes, and eligible study designs. Mechanistic terms such as serotonin, dopamine, TPH2, serotonergic, and dopaminergic were used only as optional refinements to support contextual interpretation and exploratory subgroup coding; they were not restrictive eligibility criteria.

Google Scholar was used as a supplementary discovery and citation-chasing source rather than as the sole structured bibliographic database. This supplementary search strategy increased retrieval sensitivity for hard-to-index records and supported the identification of potentially eligible primary studies through backward and forward citation chasing. However, because Google Scholar has limited transparency, unstable ranking, and reduced reproducibility compared with conventional bibliographic databases, its role was restricted to supplementary retrieval, and the structured database strategies remained fully documented in Table S1.

Records were de-duplicated before screening. Titles/abstracts and full texts were assessed independently by two reviewers against the prespecified criteria; disagreements were resolved by consensus and, when required, adjudicated by a third reviewer. A calibration exercise on a heterogeneous subset of records was conducted before full screening to harmonize decision rules while preserving the a priori protocol. Data extraction used a piloted, structured form aligned with the multi-phase analytic plan; one reviewer extracted all fields and a second reviewer independently verified them, with unresolved discrepancies adjudicated by a third reviewer.

Extracted items included study design, setting, participant characteristics, age stratum, psychiatric phenotype, exposure or intervention definition, comparator definition, outcome instrument, outcome timepoint, covariate adjustment set, baseline deficiency enrichment, and the most relevant extractable effect estimate for each prespecified exposure-outcome pairing. Study-level decisions regarding qualitative versus quantitative synthesis and the estimate carried forward are traceable in the included study synopsis and consolidated characteristics tables in the Supplementary Materials.

### 2.3. Exposure and Effect-Size Framework (Vitamin D/B_12_ Status; Supplementation Comparisons; Harmonization Rules)

The analytic framework separated evidence into four nutrient- and comparison-specific streams: vitamin D supplementation versus placebo/usual care, vitamin D status versus psychiatric outcomes, vitamin B_12_ status versus psychiatric outcomes, and vitamin B_12_ supplementation versus placebo/usual care. This structure prevented supplementation trials and observational status associations from being interpreted as equivalent forms of evidence. Status-outcome studies were interpreted primarily as associations or risk marker evidence, whereas randomized supplementation studies were interpreted as intervention evidence, subject to baseline status, dose, duration, outcome selection, adherence, and trial design.

In status-outcome studies, vitamin status was operationalized as reported by the original authors, including serum 25(OH)D, serum vitamin B_12,_ or clinically defined categories such as deficient/insufficient versus sufficient, low versus adequate, or lowest versus highest exposure category. When several contrasts were available, the primary contrast prioritized the one most directly aligned with clinical interpretation and prespecified synthesis rules. In supplementation studies, the primary contrast was the randomized intervention regimen versus placebo or usual care at the prespecified endpoint closest to treatment completion.

For cross-family comparability, eligible effects were harmonized to odds ratios (ORs) and analyzed on the natural log scale as log(OR) with corresponding standard errors. For binary outcomes, ORs were extracted as reported, preferentially using the most fully adjusted estimate in observational designs, or computed from endpoint event counts when necessary. Effect direction was coded so that OR < 1 consistently indicated a more favorable psychiatric profile in the higher-status or supplementation group; estimates were inverted where required to preserve this convention.

For continuous psychiatric symptom outcomes, standardized mean differences (SMDs; Hedges’ g) were converted to log odds ratios using the standard logistic approximation log(OR) = (*π*/√3) × SMD, with standard errors transformed on the same scale [32]. This conversion enabled a harmonized OR-based evidence map across binary and continuous outcomes. However, because symptom rating scales may deviate from the distributional assumptions underlying this approximation, OR magnitudes derived from converted continuous outcomes were interpreted qualitatively and comparatively rather than as direct clinical risk estimates.

For supplementation families, where continuous symptom scales were frequent, SMD estimates were retained as the primary interpretability metric, while OR-transformed estimates were retained as harmonized secondary cross-family metrics. Accordingly, supplementation results are interpreted in relation to both the SMD scale and the harmonized OR scale, with Table S14 of the Supplementary Materials providing the SMD-based estimates. This dual reporting preserved cross-family comparability while limiting overinterpretation of the precision and clinical meaning of ORs derived from continuous psychiatric rating scales.

To prevent unit-of-analysis errors and selective inclusion, a single prespecified primary estimate per study within each evidence family was carried forward into each pooled analysis. When multiple eligible psychiatric outcomes were available within a study, diagnosis-level outcomes were prioritized over secondary symptom scales when both addressed the same construct; in trials, the endpoint closest to treatment completion was prioritized. When multiple exposure contrasts were reported, contrasts corresponding most directly to clinically interpretable categories were prioritized, and multiple correlated comparisons from the same participants were not entered into the same pooled analysis. All selections and transformations are auditable in the study-level synthesis tables and in the Phase 3 covariate dataset [33].

### 2.4. Phenotype-Informed Exploratory Subgroup Classification (Serotonergic, Dopaminergic, Mixed/Neurodevelopmental and Multi-Axis Contexts)

Subgroup classification was prespecified as an exploratory, phenotype-informed approach for evaluating heterogeneity, not as a direct test of biological mechanism. Each included study was assigned to one broad category—serotonergic, dopaminergic, or mixed/other—based on the primary clinical phenotype underlying the extracted estimate, DSM/ICD diagnosis when available, validated symptom-defined syndrome when diagnosis was not available, and the broader biomarker/intervention context. These labels were used as study-level heuristic categories for subgroup exploration and should not be interpreted as evidence that serotonergic or dopaminergic pathways were directly measured.

The mixed/other category was defined to capture phenotypes and study configurations in which assignment to a single monoaminergic axis would be biologically or inferentially overconfident. This included neurodevelopmental disorders, biomarker-complex studies, multi-axis clinical contexts, and studies in which vitamin status was evaluated alongside one-carbon metabolism markers, inflammatory measures, or other biological systems. Classification was intentionally conservative: when a study plausibly engaged multiple neurochemical, neuroimmune, metabolic, or developmental pathways, assignment to mixed/other was preferred over forcing a single-axis label.

All subgroup assignments and the specific estimate carried forward into each analytic family are documented in the consolidated study characteristics tables and the Phase 3 covariate dataset. Age was operationalized a priori as children/adolescents (<18 years) versus adults (≥18 years) according to the recruitment frame; when both life stages were enrolled and age-stratified effects were available, these were extracted in parallel for subgroup analyses. Subgroup analyses were applied only as exploratory tools for detecting possible sources of heterogeneity and were interpreted in conjunction with stratum size, confidence intervals, prediction intervals, risk of bias, and biological plausibility [34,35].

### 2.5. Risk of Bias and Certainty of Evidence (RoB 2/NOS; GRADE)

Risk of bias was assessed independently by two reviewers using design-appropriate instruments, with disagreements resolved by consensus and third-reviewer adjudication when required. Randomized controlled trials were appraised using the revised Cochrane Risk of Bias tool (RoB 2), applying the standard domain-based algorithm for the effect of assignment to intervention at the prespecified endpoint [36]. The overall RoB 2 judgment followed the tool’s rules-based approach and reflected the least favorable domain-level rating. Observational studies were appraised with the Newcastle-Ottawa Scale (NOS), generating star-based judgments across Selection, Comparability, and Exposure/Outcome domains, with study-specific decision rationales recorded prospectively to ensure traceability [37].

Certainty of evidence was judged using the GRADE framework for each pooled estimate and, where pooling was not feasible, for single-study estimates when clinically relevant [38]. Judgments considered risk of bias, inconsistency, indirectness, imprecision, and publication bias, informed by RoB 2/NOS appraisals and by heterogeneity, prediction intervals, sensitivity analyses, and small-study assessments. Risk-of-bias and certainty judgments were not used to exclude studies from the primary syntheses; instead, they informed interpretation, sensitivity analyses, and the strength of conclusions drawn from each nutrient- and comparison-specific evidence stream.

### 2.6. Statistical Analysis (Multi-Phase Plan; Thresholds; Sensitivity; Small-Study Effects)

Quantitative syntheses were conducted on the natural-log odds ratio scale using inverse-variance random-effects models. Between-study variance was estimated using restricted maximum likelihood (REML) as *τ*^2^, with *τ* = √*τ*^2^. Pooled 95% confidence intervals were derived using the Hartung-Knapp adjustment to provide more conservative inference under heterogeneity. Statistical heterogeneity was summarized using Cochran’s *Q*, *I*^2^, *τ*^2^, and, where feasible, 95% prediction intervals to describe the expected dispersion of effects in comparable future settings. All tests were two-sided with α = 0.05, and analyses followed the prespecified multi-phase program documented in the Supplementary Materials [39,40,41,42,43].

Phase 1 estimated global pooled effects within each prespecified nutrient- and comparison-specific evidence stream. Evidence families meeting the minimum threshold of *k* ≥ 10 studies were treated as eligible for primary pooled inference and subsequent moderator exploration. Sparse evidence families were retained descriptively or as exploratory analyses but were not interpreted as providing stable or generalizable pooled estimates. Vitamin B_12_ supplementation, with *k* = 4, was therefore treated as exploratory and is interpreted primarily in the Supplementary Materials to avoid visual or inferential parity with adequately powered evidence streams.

Phase 2 evaluated exploratory effect modification by phenotype-informed subgroup category and age stratum using categorical moderator tests and between-subgroup heterogeneity statistics when study counts were sufficient. Within-stratum pooled estimates were reported only when synthesis was numerically stable, generally requiring *k* ≥ 3 studies per stratum. Strata below this threshold were described narratively or flagged as uninterpretable. In particular, subgroup estimates with very small k, very high heterogeneity, or extremely wide confidence intervals were not treated as clinically meaningful evidence of subgroup-specific effects.

Phase 3 examined residual heterogeneity using mixed-effects meta-regression on the log(OR) scale in eligible clusters, with model complexity constrained to reduce overfitting. Candidate moderators were prespecified and included age stratum, phenotype-informed subgroup category, baseline deficiency enrichment, comorbidity burden, study design features, and risk-of-bias or certainty indicators where data availability allowed. Meta-regression was avoided when study counts were insufficient, and an approximate rule of one moderator per at least 10 studies was used to reduce spurious inference.

Sensitivity and influence diagnostics included leave-one-out analyses, outlier/influence screening, comparison of key estimates under alternative reasonable analytic choices, and exploratory trim-and-fill analyses where appropriate. Potential small-study effects were examined using funnel plots and Egger-type regression tests only when *k* ≥ 10 studies were available. Trim-and-fill was interpreted cautiously, particularly under substantial heterogeneity and outcome diversity, and was used as a sensitivity indicator rather than as a definitive correction for publication bias.

For supplementation evidence families dominated by continuous symptom outcomes, SMD-based estimates were used as the primary clinical interpretability metric, whereas OR-transformed estimates were retained for harmonized cross-family presentation. Primary computations and figure generation were implemented in Python 3.12.13 using NumPy 2.0.2, pandas 2.2.2, SciPy 1.16.3, and Matplotlib 3.10.0 in Google Colab, using a scripted reproducible workflow. Pooled estimates and forest plots were independently replicated in MetaAnalysisOnline.com (accessed on 3 March 2026) using the same effect measures and harmonized model settings, including random-effects specification, *τ*^2^ estimator, Hartung-Knapp adjustment, and continuity correction where applicable [39,40,41,42,43].

## 3. Results

### 3.1. Results of the Systematic Review

#### 3.1.1. Study Selection and Eligibility

**Figure 1:**
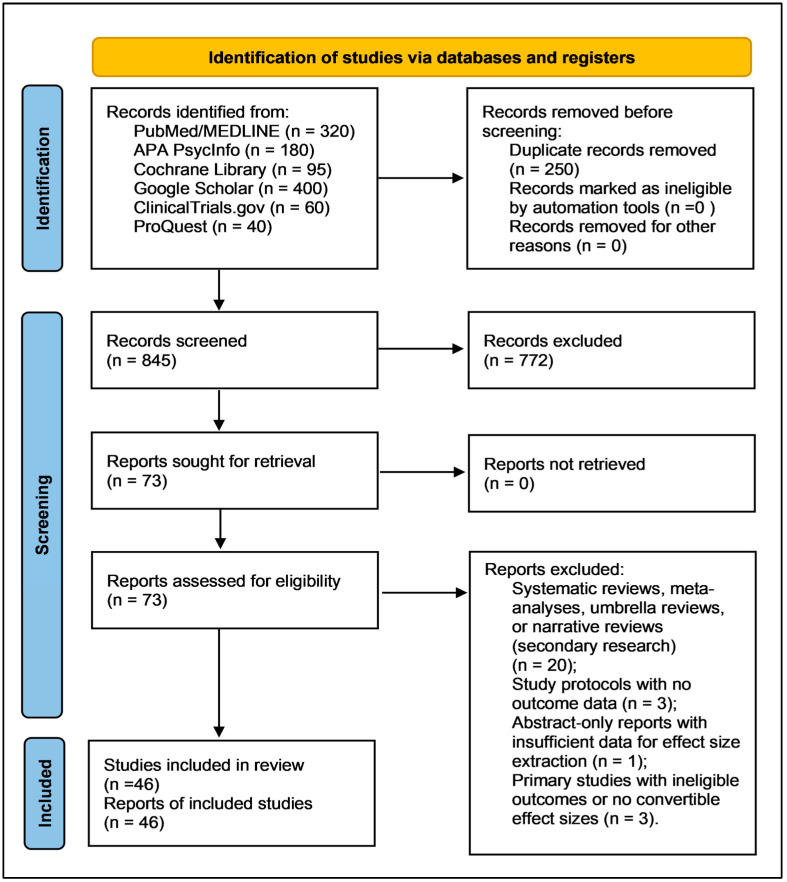
PRISMA 2020 flow diagram of study identification, screening, eligibility assessment, and inclusion. Records were retrieved from PubMed/MEDLINE, APA PsycInfo, Cochrane Library, Google Scholar, ClinicalTrials.gov, and ProQuest, then deduplicated, screened at the title/abstract level, and assessed in full text. Overall, 46 primary studies were included, while 27 full-text reports were excluded for prespecified reasons, as detailed in Table S3. The final search update was run on 1 March 2026.

The study selection process is summarized in Figure 1. Across all searched databases, registers, and supplementary sources, 1,095 records were identified. After removal of 250 duplicates, 845 records were screened at the title/abstract level, of which 772 were excluded. Seventy-three reports were retrieved and assessed in full text, yielding 46 primary studies published between 2016 and 2025 for qualitative synthesis [44,45,46,47,48,49,50,51,52,53,54,55,56,57,58,59,60,61,62,63,64,65,66,67,68,69,70,71,72,73,74,75,76,77,78,79,80,81,82,83,84,85,86,87,88,89]. Of these, 44 studies contributed extractable, protocol-harmonized quantitative data.

Twenty-seven full-text reports were excluded for prespecified reasons, as detailed in Table S3 of the Supplementary Materials [90,91,92,93,94,95,96,97,98,99,100,101,102,103,104,105,106,107,108,109,110,111,112,113,114,115,116]. These comprised 20 secondary or tertiary research reports, including systematic reviews, meta-analyses, and related evidence syntheses excluded to avoid double counting and preserve primary-study attribution; three protocol or baseline-only reports without post-intervention psychiatric outcomes; one abstract-only report without extractable quantitative data; and three primary studies with ineligible outcomes or non-convertible/unavailable effect sizes under the prespecified harmonization rules. Database-specific search strategies are reported in Table S1, while inclusion decisions are traceable through the included-study synopsis in Table S2 and the full-text exclusion log in Table S3.

#### 3.1.2. Characteristics of Included Studies and Evidence Map

Across the 46 included studies, 24 were randomized controlled trials (RCTs; 52.2%) and 22 were observational studies (47.8%), including case-control studies (*n* = 10), cross-sectional studies (*n* = 7), prospective cohort studies (*n* = 4), and one registry-based nested design (*n* = 1) (Table S4, Supplementary Materials). The evidence map populated four prespecified nutrient- and comparison-specific evidence streams: vitamin D supplementation comparisons (*n* = 18; *k* = 17 placebo/usual-care-controlled trials plus *k* = 1 non-placebo active-comparator dosing trial), vitamin D status-outcome associations (*n* = 13), vitamin B_12_ status-outcome associations (*n* = 10), and vitamin B_12_ supplementation comparisons (*n* = 5; *k* = 4 placebo/usual-care-controlled trials plus *k* = 1 standard-care trial without placebo).

Consistent with the prespecified *k* ≥ 10 threshold for primary pooled inference and moderator exploration, only vitamin D supplementation, vitamin D status, and vitamin B_12_ status progressed to Phase 2 subgroup analyses and Phase 3 meta-regression. Vitamin B_12_ supplementation, represented by only four placebo/usual-care-controlled trials, was retained as sparse exploratory evidence and is reported primarily in the Supplementary Materials to avoid visual or inferential parity with adequately powered evidence streams. Overall, 44 of 46 studies yielded extractable, protocol-harmonized inputs for quantitative synthesis; 40 studies contributed to the three *k* ≥ 10 evidence streams eligible for Phase 2–3 analyses, whereas the four placebo-controlled vitamin B_12_ supplementation trials were synthesized descriptively/exploratorily only.

Publication years ranged from 2016 to 2025, with the highest density in 2020 (9/46) and a median publication year of 2019. Geographically, studies spanned 18 countries across five continents, with the largest contribution from Asia (22/46; predominantly Iran and Turkey), followed by Europe (13/46), North America (6/46), Oceania (3/46), and Africa (2/46) (Table S4, Supplementary Materials). The cumulative sample across included studies comprised 69,902 participants, predominantly adults (60,904; 87.1%), with children/adolescents contributing 8998 participants (12.9%). Sex was reported for 69,624 participants (99.6%), including 36,973 males (53.1%) and 32,651 females (46.9%); sex was not reported for 278 participants (0.4%).

A weighted mean age could be computed for 64,941 participants (92.9%) with mean age available, yielding an overall weighted mean age of 52.6 years, with age-stratified weighted means of 59.9 years in adult samples and 6.1 years in pediatric samples (Table S4, Supplementary Materials). For exploratory subgrouping, 18/46 studies (39.1%) were classified as serotonergic phenotype-informed contexts, 10/46 (21.7%) as dopaminergic phenotype-informed contexts, and 18/46 (39.1%) as mixed/other contexts. These labels were used only as study-level exploratory categories for heterogeneity assessment and should not be interpreted as direct evidence that serotonergic or dopaminergic pathway activity was measured. Because several studies enrolled more than one diagnostic group, diagnosis frequencies were not necessarily mutually exclusive; however, each study was assigned to a single phenotype-informed exploratory category for Phase 2 subgrouping and Phase 3 moderator analyses to maintain a consistent unit of inference.

#### 3.1.3. Risk of Bias and Certainty of Evidence

**Figure 2:**
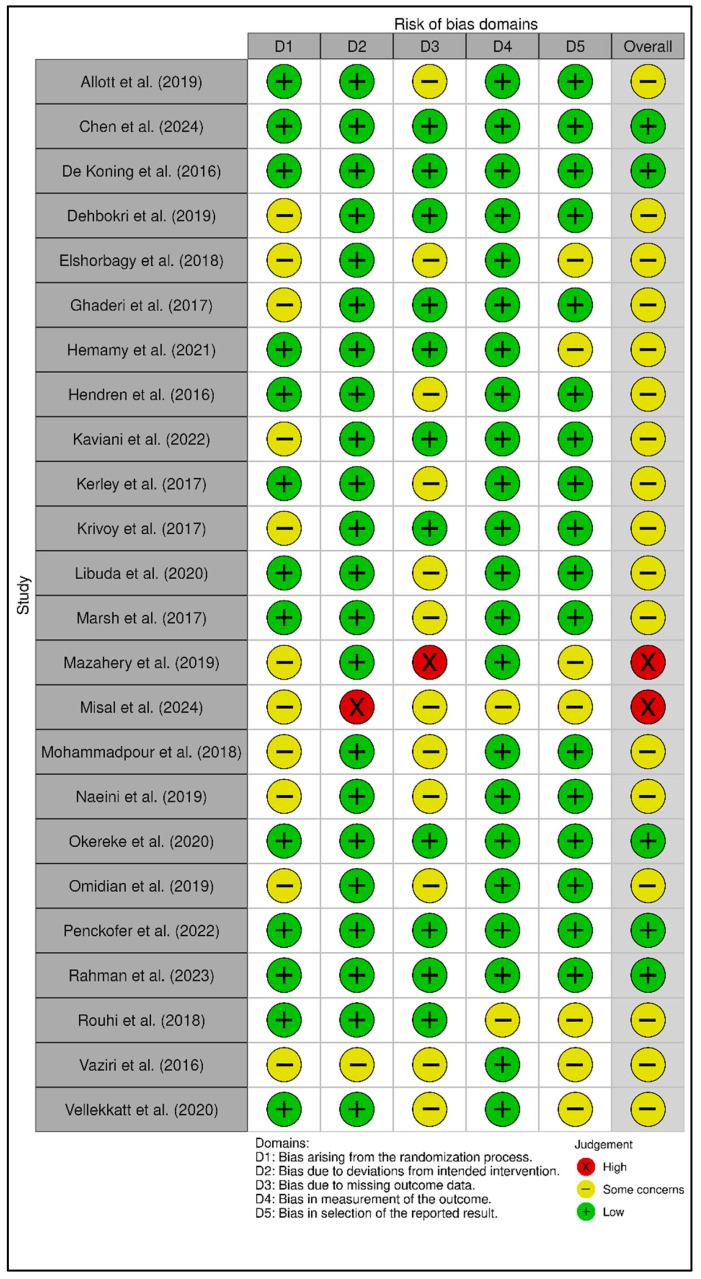
Risk-of-bias traffic-light plot for randomized controlled trials (RoB 2). Domainl-evel risk-of-bias judgments for each included randomized controlled trial, assessed using the revised Cochrane Risk of Bias tool. Domains are: D1, bias arising from the randomization process; D2, bias due to deviations from intended interventions; D3, bias due to missing outcome data; D4, bias in measurement of the outcome; and D5, bias in selection of the reported result, together with the overall RoB 2 judgment. Color coding indicates low risk of bias, some concerns, and high risk of bias [45,49,50,51,53,59,60,61,63,64,65,67,69,70,71,72,73,75,76,77,79,80,85,86].

**Figure 3:**
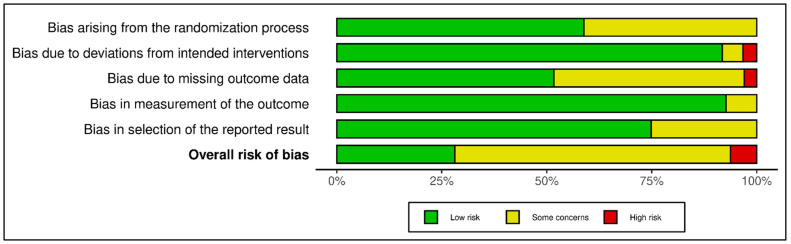
Risk-of-bias summary plot for randomized controlled trials (RoB 2). Summary distribution of RoB 2 judgments across the included randomized controlled trials, shown as the proportion of studies rated low risk of bias, some concerns, or high risk of bias within each RoB 2 domain and for the overall judgment [45,49,50,51,53,59,60,61,63,64,65,67,69,70,71,72,73,75,76,77,79,80,85,86].

For the 24 RCTs [45,49,50,51,53,59,60,61,63,64,65,67,69,70,71,72,73,75,76,77,79,80,85,86], the overall RoB 2 judgments were most frequently “some concerns” (17/24), with 5/24 rated as low risk of bias and 2/24 rated as high risk of bias (Table S5, Supplementary Materials; Figure 2 and Figure 3). At the domain level, uncertainty most often arose from the randomization process (some concerns/high in 11/24) and missing outcome data (some concerns/high in 13/24), whereas deviations from intended interventions and outcome measurement were generally judged as low risk. GRADE certainty for RCT-derived estimates was predominantly moderate (16/24), with 7/24 rated low and 1/24 rated high (Table S5, Supplementary Materials).

Observational studies [44,46,47,48,52,54,55,56,57,58,62,66,68,74,78,81,82,83,84,87,88,89] showed NOS total scores ranging from 4 to 9, with a median score of 7. GRADE certainty for observational estimates was predominantly low (14/22) or very low (7/22), while one observational estimate reached moderate certainty (Table S6, Supplementary Materials). These certainty patterns support cautious interpretation of observational status-outcome associations, particularly when pooled estimates are accompanied by substantial heterogeneity, wide prediction intervals, or residual confounding.

#### 3.1.4. Qualitative Synthesis Across Diagnoses and Nutrient-Specific Evidence Streams

The qualitative synthesis was used to contextualize the quantitative evidence streams and to distinguish association signals from intervention effects across psychiatric phenotypes. Because included studies differed substantially in design, age group, baseline nutrient status, psychiatric diagnosis, intervention structure, outcome instrument, and follow-up duration, the qualitative synthesis was not intended to establish a uniform nutrient effect. Instead, it identifies recurring patterns of heterogeneity and highlights contexts in which nutrient status or supplementation may be clinically informative.

The diagnosis-level synthesis should therefore be interpreted as an evidence map rather than as confirmatory mechanistic evidence. Phenotype-informed categories are used descriptively to organize the literature and to support exploratory heterogeneity assessment; they do not indicate direct measurement of serotonergic, dopaminergic, neuroimmune, astroglial, or metabolic pathway activity in the primary studies.

Across depression-spectrum phenotypes, vitamin B_12_ status signals were heterogeneous and appeared sensitive to phenotype, life stage, and analytic context. Anmella et al. (2025) [47] reported lower vitamin B_12_ concentrations and higher rates of insufficiency in depressive disorders, whereas Dhiman et al. (2021) [52] observed higher B_12_ associated with lower odds of postpartum depression. In contrast, Erensoy (2020) [55] did not detect clear B_12_ differences when comparing depressive and anxiety disorders. Pediatric affective/obsessive phenotypes showed more consistent deficiency patterns, with Esnafoglu & Ozturan (2020) [56] reporting lower B_12_ in pediatric depressive disorder and Esnafoğlu & Yaman (2017) [57] reporting reduced B_12_ in pediatric obsessive-compulsive disorder. Large population analyses were directionally mixed: Huang et al. (2018) [62] found higher B_12_ linked to higher odds of depressive symptoms in adjusted analyses, whereas Laird et al. (2023) [66] reported that low baseline B_12_ (but not folate) predicted incident depressive symptoms. In supplementation evidence, the large prevention-oriented trial de Koning et al. (2016) [50] did not reduce clinically relevant depressive symptoms after two years of vitamin B_12_ plus folic acid (with vitamin D present as background exposure in both arms), while Misal et al. (2024) [71] reported improvement with B_12_ augmentation in late-life depressive symptoms but was not quantitatively pooled due to its non-placebo comparator structure.

Vitamin D supplementation trials in depressive phenotypes were similarly mixed and suggested context dependence, with more prominent symptom signals in short-term, deficiency-enriched, or perinatal samples than in long-term prevention settings. Kaviani et al. (2022) [63] and Omidian et al. (2019) [76] reported greater reductions in depressive symptom severity versus placebo in adults with baseline vitamin D deficiency (including a type 2 diabetes cohort), and Ghaderi et al. (2017) [59] observed modest symptom improvement in a methadone maintenance sample. In perinatal settings, Vaziri et al. (2016) [85] and Rouhi et al. (2018) [80] reported lower postpartum depressive symptom scores with vitamin D_3_ versus placebo. In contrast, the dose-comparison RCT Penckofer et al. (2022) [77] did not show a clear advantage of higher versus lower vitamin D_3_ dosing and was retained for qualitative synthesis only. In adolescents, Libuda et al. (2020) [67] did not demonstrate an effect on self-rated depressive symptoms, while large, long-duration trials in general populations Okereke et al. (2020) [75] and Rahman et al. (2023) [79] reported null effects of sustained vitamin D_3_ supplementation on depressive outcomes. In bipolar depression, Marsh et al. (2017) [69] did not show a clear incremental benefit of vitamin D_3_ over placebo.

In psychosis-spectrum phenotypes, vitamin D status studies consistently documented a high prevalence of hypovitaminosis D and inverse associations with psychotic disorder status across diverse settings, while the observational design precluded inference on causality or directionality. Boerman et al. (2016) [48] reported widespread deficiency in outpatient severe mental illness without independent diagnostic prediction after adjustment, whereas Van der Leeuw et al. (2020) [84] reported lower vitamin D concentrations in psychotic disorder versus controls in adjusted analyses. In inpatient contexts, Endres et al. (2016) [54] documented severe deficiency in adult inpatients with schizophreniform presentations and neurodevelopmental syndromes, and Fabrazzo et al. (2022) [58] reported lower vitamin D in acutely relapsing inpatients versus stabilized outpatients. Case-control studies similarly reported lower vitamin D in schizophrenia compared with controls, including Okasha et al. (2020) [74] and Shahini et al. (2022) [81]. In first-episode psychosis, Yee et al. (2016) [88] suggested that bioavailable vitamin D may better differentiate cases from controls and correlate inversely with negative symptoms. Complementary one-carbon evidence in psychosis included Yazici et al. (2019) [87], who reported substantially higher odds of vitamin B_12_ deficiency in schizophrenia. Interventional studies did not provide consistent support for adjunctive vitamin D or B_12_ improving core symptom outcomes over short follow-up: Krivoy et al. (2017) [65] increased 25(OH)D without improving Positive and Negative Syndrome Scale (PANSS) total scores over eight weeks, while Allott et al. (2019) [45] and Chen et al. (2024) [49] reported no significant symptomatic benefit of B-vitamin/B_12_-folate adjunctive strategies relative to controls despite expected biomarker changes.

In neurodevelopmental phenotypes, vitamin D and one-carbon markers were investigated both as status correlates and as supplementation targets. For Autism Spectrum Disorder (ASD) risk and diagnosis, cohort data were mixed: Ali et al. (2019) [44] and Madley-Dowd et al. (2022) [68] did not show clear associations between vitamin D status and ASD, whereas Sourander et al. (2021) [82] reported higher odds of ASD with low maternal vitamin D in early pregnancy. In pediatric ASD case-control work, Petruzzelli et al. (2020) [78] observed lower vitamin D levels and higher deficiency prevalence in children with ASD. In ASD intervention trials, Kerley et al. (2017) [64] and Mazahery et al. (2019) [70] increased 25(OH)D without demonstrating clear benefits on prespecified core ASD symptom endpoints, whereas Hendren et al. (2016) [61] reported clinical improvement with methylcobalamin. One-carbon status correlates with Autism Spectrum Disorder/Attention-Deficit/Hyperactivity Disorder (ADHD) included Altun et al. (2018) [46] and Yektaş et al. (2019) [89], both reporting lower vitamin B_12_ (and higher homocysteine) relative to controls, with symptom-biomarker relationships varying by phenotype. In ADHD, multiple placebo-controlled RCTs tested vitamin D supplementation, frequently in pediatric, deficiency-enriched samples and often as an adjunct to standard therapy. Dehbokri et al. (2019) [51], Elshorbagy et al. (2018) [53], Mohammadpour et al. (2018) [72], and Naeini et al. (2019) [73] reported short-term improvements in ADHD symptom ratings with vitamin D_3_ versus placebo, while Hemamy et al. (2021) [60] suggested additional benefit when vitamin D was combined with magnesium for behavioral difficulties. Collectively, these trials support biologically plausible symptom signals under conditions of baseline insufficiency, while remaining limited by short follow-up and variability in outcome operationalization.

Finally, several studies addressed mixed or other phenotypes linking vitamin D status or supplementation to broader stress-related or medically complex contexts. Terock et al. (2020) [83] reported an inverse association between 25(OH)D and post-traumatic stress disorder odds after adjustment, with attenuation after accounting for trauma load, whereas Vellekkatt et al. (2020) [86] reported clinician-rated symptom improvement with adjunctive parenteral vitamin D in major depression. Overall, the qualitative evidence most consistently identified low vitamin D status as a common correlate in severe mental illness and selected neurodevelopmental or stress-related contexts, whereas interventional findings were more heterogeneous and appeared sensitive to baseline deficiency, phenotype, outcome definition, comparator structure, and trial duration.

Overall, the qualitative synthesis supports the same interpretive direction as the quantitative analyses: evidence signals are heterogeneous, design-dependent, and context-sensitive. Observational vitamin status findings may identify deficiency-linked vulnerability or broader health status differences, but they do not establish causal protection. Supplementation trials provide more direct intervention evidence, but their findings vary by baseline deficiency enrichment, psychiatric phenotype, trial duration, comparator structure, and outcome scale. Consequently, the qualitative evidence supports targeted assessment and hypothesis generation rather than routine supplementation recommendations across unselected psychiatric populations.

### 3.2. Exploratory Quantitative Synthesis and Meta-Analysis

The quantitative synthesis was structured according to the prespecified nutrient- and comparison-specific evidence streams described above. Primary pooled inference was restricted to evidence families meeting the *k* ≥ 10 threshold: vitamin D supplementation versus placebo/usual care, vitamin D status versus psychiatric outcomes, and vitamin B_12_ status versus psychiatric outcomes. Vitamin B_12_ supplementation, represented by only four placebo-controlled randomized comparisons, was retained as sparse exploratory evidence and is reported primarily in the Supplementary Materials. The results below therefore emphasize heterogeneity, prediction intervals, sensitivity analyses, and the distinction between observational status associations and randomized supplementation evidence.

#### 3.2.1. Global Meta-Analysis (Phase 1)

Phase 1 summarized pooled estimates within prespecified nutrient- and comparison-specific evidence streams, rather than across all included studies as a single clinically homogeneous body of evidence. Three evidence streams met the prespecified *k* ≥ 10 threshold for primary global pooling: vitamin D supplementation versus placebo/usual care, vitamin D status versus psychiatric outcomes, and vitamin B_12_ status versus psychiatric outcomes. Vitamin B_12_ supplementation versus placebo/usual care was retained as sparse exploratory evidence because only four placebo-controlled randomized comparisons were available and this evidence family did not meet the threshold for Phase 2 subgroup analysis, Phase 3 meta-regression, funnel plot assessment, or generalizable pooled inference (Table S7, Supplementary Materials).

Across the three primary evidence streams, pooled estimates showed inverse point estimates but were consistently characterized by substantial or extreme heterogeneity, wide prediction intervals, and sensitivity findings that limited generalizability. Consequently, Phase 1 results should be interpreted as exploratory summaries within heterogeneous evidence streams rather than as stable mean effects generalizable across psychiatric diagnoses, age groups, baseline nutrient status, intervention contexts, or outcome formats.

A. Vitamin D Supplementation versus Placebo/Usual Care

Seventeen randomized comparisons evaluating vitamin D supplementation versus placebo/usual care were included in the primary supplementation evidence stream. On the harmonized odds-ratio scale, the random-effects model yielded an initial inverse pooled estimate (OR = 0.439, 95% CI 0.272–0.710; *p* = 0.0023), suggesting lower odds of adverse psychiatric outcomes in the supplementation arms under the prespecified direction of effect (Table 1). However, the pooled estimate was accompanied by substantial heterogeneity (*Q* = 101.30, *df* = 16, *p* < 0.0001; *I*^2^ = 84.2%; *τ*^2^ = 0.5820), indicating that the included trials did not estimate a common, clinically stable intervention effect.

Because supplementation trials frequently relied on continuous psychiatric symptom scales, the standardized mean difference (SMD) scale provides the primary metric for clinical interpretability in this evidence stream. The corresponding pooled SMD was −0.454 (95% CI −0.718 to −0.189), directionally consistent with symptom improvement in the supplementation group, but still accompanied by substantial heterogeneity (*I*^2^ = 84.2%; *τ*^2^ = 0.1769 on the SMD scale; Table S14, Supplementary Materials). Therefore, the OR-transformed estimate should be read as a harmonized cross-family metric, whereas the SMD estimate is more clinically interpretable for supplementation trials.

**Figure 4:**
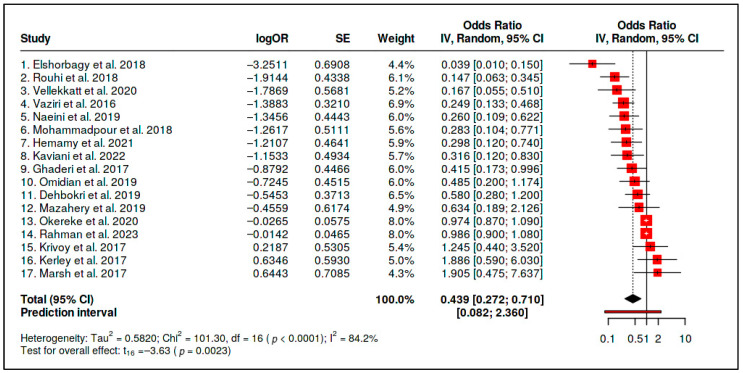
Random-effects forest plot of vitamin D supplementation versus placebo/usual care on psychiatric outcomes. Study-specific odds ratios (ORs) and 95% confidence intervals (CIs) are displayed on a logarithmic scale. Red squares represent individual study estimates, with square size proportional to inverse-variance weight under the random-effects model; horizontal black lines represent 95% CIs. The white cross indicates the point estimate within each study marker. The pooled random-effects estimate is shown as a summary diamond, and the vertical reference line at OR = 1 denotes the null effect. The summary estimate showed an initial inverse pooled association (OR = 0.439, 95% CI 0.272–0.710), but heterogeneity was substantial (*I*^2^ = 84.2%; *τ*^2^ = 0.5820), limiting interpretation as a stable intervention effect across psychiatric populations [51,53,59,60,63,64,65,69,70,72,73,75,76,79,80,85,86].

The primary forest plot illustrates both the inverse average estimate and the considerable between-study dispersion. Several small trials contributed large inverse estimates, whereas other trials were close to the null or directionally inconsistent (Figure 4). This distribution supports quantitative pooling only as an exploratory summary and reinforces the need to interpret the pooled estimate alongside prediction intervals and small-study-effect sensitivity analyses.

The initial summary statistics are presented in Table 2. Although the pooled point estimate was inverse and statistically significant, the prediction interval crossed unity, heterogeneity was substantial, and Egger’s test indicated funnel-plot asymmetry. Therefore, the initial pooled estimate cannot be interpreted as evidence of a stable generalizable supplementation effect without the corresponding trim-and-fill sensitivity analysis.

**Figure 5:**
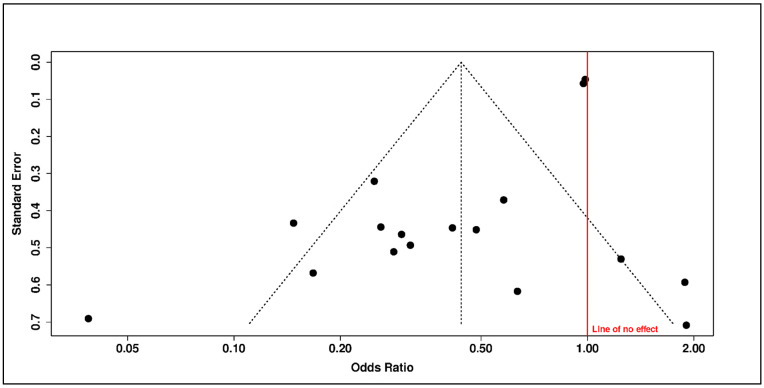
Funnel plot for assessing small-study effects in the random-effects meta-analysis of vitamin D supplementation versus placebo/usual care. Each point represents an individual comparison, plotted as the odds ratio (OR) against the standard error of log(OR). The vertical reference line denotes the null effect at OR = 1, and the triangular boundaries indicate approximate pseudo-95% limits. Visual asymmetry supports caution in interpreting the primary pooled estimate and motivates trim-and-fill sensitivity analysis.

**Figure 6:**
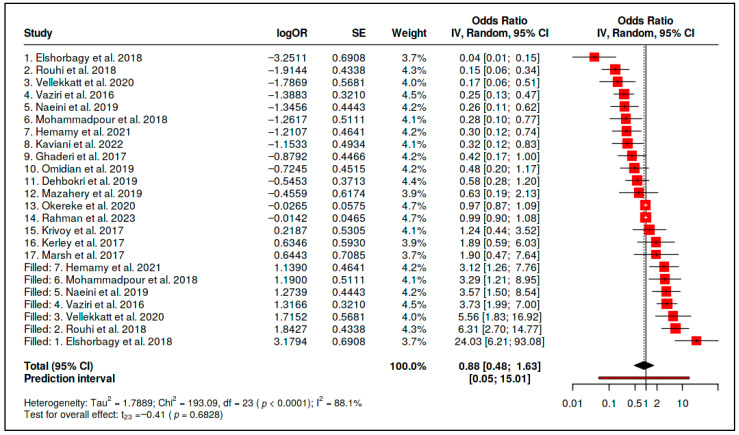
Trim-and-fill-adjusted random-effects meta-analysis of vitamin D supplementation versus placebo/usual care on psychiatric outcomes. Observed studies are listed first, followed by imputed studies added by the Duval and Tweedie trim-and-fill procedure to account for funnel-plot asymmetry. Odds ratios (ORs) and 95% confidence intervals (CIs) are shown on a logarithmic scale. Red squares represent individual observed study estimates, while imputed studies are displayed according to the trim-and-fill output; marker size is proportional to inverse-variance weight under the random-effects model. Horizontal black lines represent 95% CIs, and the white cross indicates the point estimate within each study marker. The pooled random-effects estimate is shown as a summary diamond, and the vertical reference line at OR = 1 denotes the null effect. After imputation of seven studies (adjusted *k* = 24), the pooled estimate attenuated to the null (adjusted OR = 0.88, 95% CI 0.48–1.63; *p* = 0.6828), with high residual heterogeneity (*I*^2^ = 88.1%; *τ*^2^ = 1.7889) and a very wide prediction interval (0.05–15.01), indicating limited robustness and poor clinical portability [51,53,59,60,63,64,65,69,70,72,73,75,76,79,80,85,86].

Small-study-effect assessment further weakened the robustness of the vitamin D supplementation signal. Funnel-plot asymmetry was consistent with an overrepresentation of small studies with favorable estimates (Figure 5 and Figure 6). This pattern indicates that the primary pooled estimate may overstate the average effect and should not be interpreted without the corresponding sensitivity analysis.

Duval and Tweedie trim-and-fill imputed seven potentially missing studies to restore funnel symmetry (Figure 6). After imputation, the adjusted pooled estimate was attenuated to the null (adjusted OR = 0.88, 95% CI 0.48–1.63; *p* = 0.6828). Adjusted heterogeneity remained high (*I*^2^ = 88.1%; *τ*^2^ = 1.7889), and the adjusted 95% prediction interval was very wide (0.05–15.01), indicating that future comparable studies could plausibly observe benefit, no effect, or harm depending on clinical and methodological context (Table 3).

Taken together, the vitamin D supplementation evidence does not support a generalizable psychiatric benefit across unselected populations. The primary estimate suggests an initial inverse average signal, but this signal is not robust to small-study-effect adjustment and is embedded in substantial between-study variability. The appropriate inference is therefore conditional and exploratory: vitamin D supplementation may warrant further study in deficiency-enriched or clinically selected subgroups, but the pooled evidence does not justify routine adjunctive supplementation across heterogeneous psychiatric populations.

B. Vitamin D Status versus Psychiatric Outcomes

**Figure 7:**
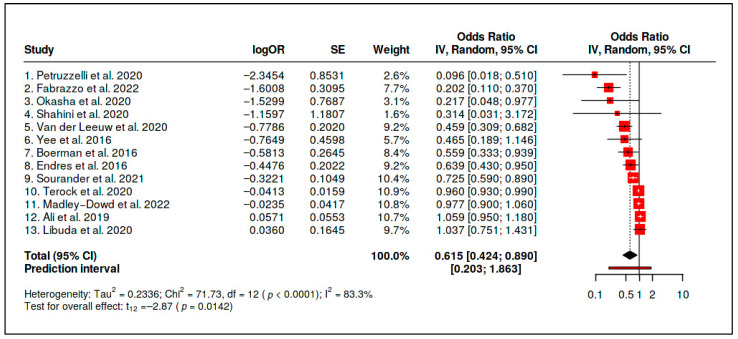
Random-effects forest plot of vitamin D status versus psychiatric outcomes. Study-specific odds ratios (ORs) and 95% confidence intervals (CIs) are shown on a logarithmic scale. OR < 1 denotes a more favorable psychiatric profile in the higher-status or lower-deficiency group according to the harmonized direction of effect. Red squares represent individual study estimates, with square size proportional to inverse-variance weight under the random-effects model; horizontal black lines represent 95% CIs. The white cross indicates the point estimate within each study marker. The pooled random-effects estimate is shown as a summary diamond, and the vertical reference line at OR = 1 denotes the null effect. The pooled estimate was inverse (OR = 0.615, 95% CI 0.424–0.890), but heterogeneity was substantial (*I*^2^ = 83.3%; *τ*^2^ = 0.2336), indicating limited portability across observational contexts [44,48,54,58,67,68,74,78,81,82,83,84,88].

Thirteen observational effect estimates evaluating vitamin D status in relation to psychiatric outcomes were included in the primary vitamin D status evidence stream. The random-effects model yielded an inverse association between higher vitamin D status and adverse psychiatric outcomes (OR = 0.615, 95% CI 0.424–0.890; *p* = 0.0142). However, heterogeneity was substantial (*Q* = 71.73, *df* = 12, *p* < 0.0001; *I*^2^ = 83.3%; *τ*^2^ = 0.2336), indicating that the pooled association represented an average across clinically and methodologically diverse contexts rather than a uniform status-outcome relationship (Table 4 and Figure 7).

The initial summary statistics are presented in Table 5. Although the primary pooled estimate was inverse, the prediction interval crossed unity and heterogeneity was substantial. These findings indicate that the observational vitamin D status association should be interpreted cautiously and should not be treated as a clinically portable protective effect.

**Figure 8:**
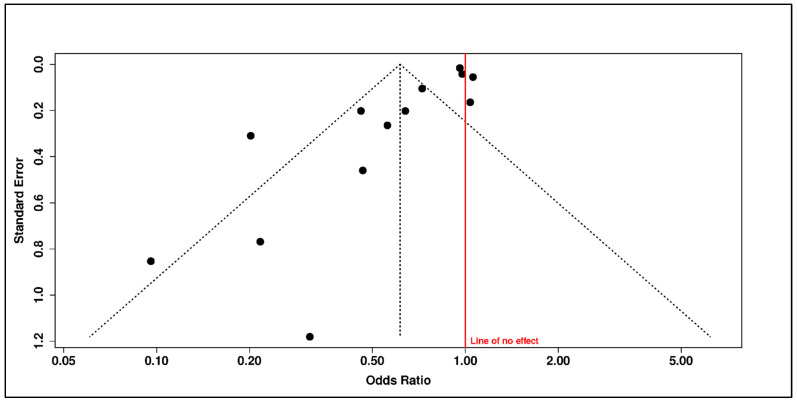
Funnel plot for assessing small-study effects in the random-effects meta-analysis of vitamin D status versus psychiatric outcomes. Each point represents an individual observational estimate, plotted as the odds ratio (OR) against the standard error of log(OR). The vertical reference line denotes the null effect at OR = 1, and the triangular boundaries indicate approximate pseudo-95% limits. Visual and statistical asymmetry support caution in interpreting the primary pooled association and justify trim-and-fill sensitivity analysis [44,48,54,58,67,68,74,78,81,82,83,84,88].

**Figure 9:**
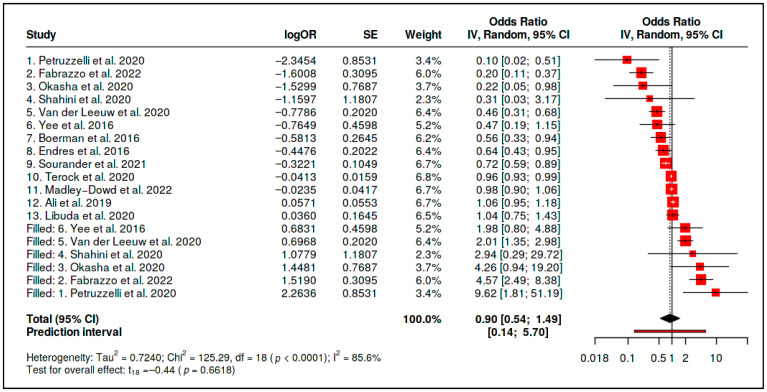
Trim-and-fill-adjusted random-effects meta-analysis of vitamin D status versus psychiatric outcomes. Observed and imputed estimates are shown on the odds-ratio scale after applying the Duval and Tweedie trim-and-fill procedure. Red squares represent individual observed study estimates, while imputed studies are displayed according to the trim-and-fill output; marker size is proportional to inverse-variance weight under the random-effects model. Horizontal black lines represent 95% confidence intervals (CIs), and the white cross indicates the point estimate within each study marker. The pooled random-effects estimate is shown as a summary diamond, and the vertical reference line at OR = 1 denotes the null effect. The adjusted pooled estimate attenuated to the null (adjusted OR = 0.90, 95% CI 0.54–1.49), and the adjusted prediction interval remained wide (0.14–5.70), indicating that the primary inverse association is not robust to small-study-effect adjustment [44,48,54,58,67,68,74,78,81,82,83,84,88].

Sensitivity analyses further limited the robustness of this association. Funnel-plot asymmetry and trim-and-fill adjustment attenuated the estimate to the null (adjusted OR = 0.90, 95% CI 0.54–1.49), while the adjusted prediction interval remained wide and crossed unity (0.14–5.70). These findings indicate that the apparent inverse association between vitamin D status and psychiatric outcomes is vulnerable to small-study effects, publication-related asymmetry, residual confounding, or context-specific differences in population structure and exposure measurement (Figure 8 and Figure 9 and Table 6).

The vitamin D status evidence should therefore be interpreted as observational association evidence rather than as evidence of supplementation benefit or causal protection. Higher vitamin D status may identify healthier baseline profiles, lower inflammatory or metabolic burden, different lifestyle characteristics, or deficiency-related vulnerability in selected populations. However, the available evidence does not establish a clinically portable protective status effect across psychiatric phenotypes.

C. Vitamin B_12_ Status versus Psychiatric Outcomes

**Figure 10:**
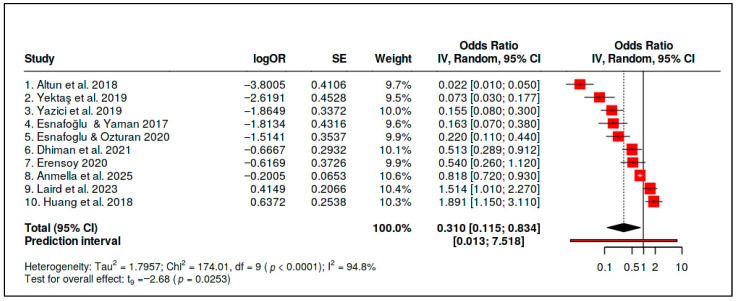
Random-effects forest plot of vitamin B_12_ status versus psychiatric outcomes. Study-specific odds ratios (ORs) and 95% confidence intervals (CIs) are displayed on a logarithmic scale. OR < 1 denotes a more favorable psychiatric profile in the higher-status or lower-deficiency group according to the harmonized effect direction. Red squares represent individual study estimates, with square size proportional to inverse-variance weight under the random-effects model; horizontal black lines represent 95% CIs. The white cross indicates the point estimate within each study marker. The pooled random-effects estimate is shown as a summary diamond, and the vertical reference line at OR = 1 denotes the null effect. The pooled estimate was inverse (summary OR = 0.310, 95% CI 0.115–0.834; *p* = 0.0253), but heterogeneity was extreme (*I*^2^ = 94.8%; *τ*^2^ = 1.7957), and the prediction interval was very wide, limiting clinical portability [46,47,52,55,56,57,62,66,87,89].

Ten observational effect estimates evaluating vitamin B_12_ status in relation to psychiatric outcomes were included in the primary vitamin B_12_ status evidence stream. The pooled random-effects estimate was inverse and statistically significant (OR = 0.310, 95% CI 0.115–0.834; *p* = 0.0253), suggesting that a more favorable vitamin B_12_ status was associated, on average, with lower odds of adverse psychiatric outcomes. This result, however, was accompanied by extreme heterogeneity (*Q* = 174.01, *df* = 9, *p* < 0.0001; *I*^2^ = 94.8%; *τ*^2^ = 1.7957) (Table 7 and Figure 10).

The prediction interval was extremely wide (0.013–7.518), spanning effects from strongly inverse to null or adverse. This interval indicates that the pooled estimate is not clinically portable and should not be interpreted as a generalizable B_12_ status effect across psychiatric contexts. Instead, it summarizes a highly heterogeneous literature in which baseline deficiency, age, psychiatric phenotype, assay context, comorbidity burden, and analytic adjustment likely vary substantially across studies (Table 8).

**Figure 11:**
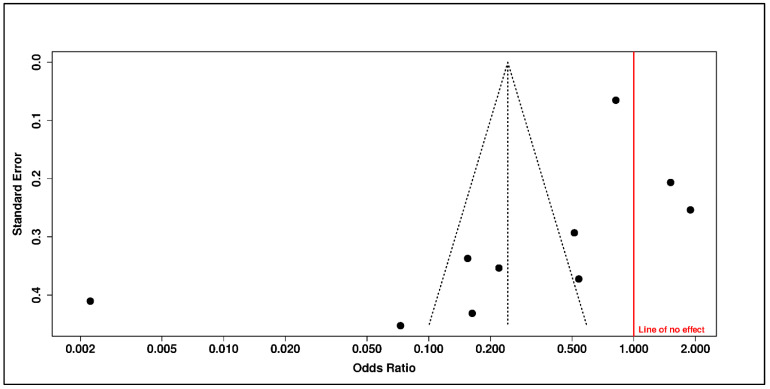
Funnel plot for assessing small-study effects and potential publication bias in the random effects meta-analysis of vitamin B_12_ status versus psychiatric outcomes. Each point represents an individual study, plotted as the odds ratio (OR) against the standard error of log(OR). The vertical reference line denotes the null effect at OR = 1, and the dashed triangular boundaries indicate approximate pseudo-95% limits. Visual inspection did not reveal strong asymmetry, and Egger’s regression test was not statistically significant (intercept = −4.71, 95% CI −9.46 to 0.04; *p* = 0.088), but interpretation remains constrained by extreme heterogeneity and the wide prediction interval [46,47,52,55,56,57,62,66,87,89].

Egger’s test did not reach statistical significance for vitamin B_12_ status (intercept = −4.71, 95% CI −9.46 to 0.04; *p* = 0.088), but this does not resolve the interpretive problem created by extreme heterogeneity and the very wide prediction interval. The B_12_ status result is therefore best interpreted as a context-dependent, deficiency-linked association signal that supports clinical attention to possible reversible deficiency in selected patients, rather than as evidence of a stable population-wide psychiatric effect (Figure 11).

D. Sparse Vitamin B_12_ Supplementation Evidence

Placebo-controlled vitamin B_12_ supplementation evidence was sparse, with only four randomized comparisons available. The pooled OR was null (OR = 0.989, 95% CI 0.563–1.737; *p* = 0.9525), and the corresponding pooled SMD was essentially zero (SMD = −0.006, 95% CI −0.317 to 0.304; Table S14, Supplementary Materials). Heterogeneity was lower than in the primary evidence streams (*I*^2^ = 23.5%; *τ*^2^ = 0.0148), but the small number of studies precluded reliable subgroup analysis, meta-regression, funnel-plot assessment, or generalizable inference.

Because this evidence family did not meet the prespecified *k* ≥ 10 threshold, detailed tables for vitamin B_12_ supplementation are reported in the Supplementary Materials rather than in the main manuscript. This placement avoids visual or inferential parity with adequately powered evidence streams while preserving transparency. The available placebo-controlled B_12_ supplementation evidence does not support a routine adjunctive supplementation effect in unselected psychiatric populations, but its sparse size prevents strong exclusion of benefit in deficiency-enriched or biologically selected subgroups.

#### 3.2.2. Subgroup and Exploratory Analyses (Phase 2)

Phase 2 evaluated whether age strata and phenotype-informed subgroup categories could account for part of the heterogeneity observed in Phase 1. These analyses were exploratory and should not be interpreted as direct tests of serotonergic or dopaminergic biology, because most included studies did not directly measure monoaminergic pathway biomarkers or pathway activity. The subgroup labels therefore functioned as study-level phenotype-informed categories rather than as evidence of mechanism.

Subgroup analyses were restricted to evidence families with sufficient study counts for moderator exploration. A single prespecified effect per study was used for Phase 2 synthesis, and subgroup assignments are reported in Table S8 of the Supplementary Materials. Within-stratum pooled estimates were reported only when at least three studies were available, whereas strata with *k* < 3 were not pooled. All subgroup findings were interpreted in relation to stratum size, heterogeneity, confidence intervals, and residual uncertainty.

For vitamin D supplementation, the serotonergic phenotype-informed stratum showed an inverse pooled estimate (OR = 0.406, 95% CI 0.194–0.848), but heterogeneity was very high (*I*^2^ = 88.7%). The mixed/other stratum showed a directionally similar but non-definitive estimate (OR = 0.519, 95% CI 0.241–1.118; *I*^2^ = 55.1%). The dopaminergic supplementation stratum was clinically and statistically uninterpretable because it included only three studies, showed very high heterogeneity (*I*^2^ = 87.8%), and produced an extremely wide confidence interval (OR = 0.324, 95% CI 0.004–26.712). This stratum should therefore not be interpreted as evidence of a dopaminergic supplementation effect.

For vitamin D status, the dopaminergic phenotype-informed stratum yielded an inverse pooled estimate with negligible statistical heterogeneity (OR = 0.487, 95% CI 0.399–0.593; *I*^2^ = 0.0%), whereas the mixed/other stratum was heterogeneous and non-definitive (OR = 0.582, 95% CI 0.304–1.114; *I*^2^ = 86.5%). The serotonergic status stratum contained only one study and was not pooled. These results suggest possible phenotype-related variability in observational vitamin D status associations, but they do not establish pathway-specific biology.

For vitamin B_12_ status, only the serotonergic phenotype-informed stratum had enough studies for pooling (*k* = 7). Its estimate was inverse but imprecise and statistically non-definitive (OR = 0.610, 95% CI 0.264–1.410), with very high heterogeneity (*I*^2^ = 88.4%). The dopaminergic and mixed/other strata were too sparse for pooling. Therefore, even though between-stratum differences were detected in formal moderator testing, the biological interpretation of these differences remains limited by sparse strata and extreme residual heterogeneity.

Formal between-subgroup tests did not support phenotype-level separation for vitamin D supplementation (*Q*-between = 0.519, *df* = 2, *p* = 0.771) or vitamin D status (*Q*-between = 1.774, *df* = 2, *p* = 0.412). For vitamin B_12_ status, the phenotype-informed moderator test was statistically significant (*Q*-between = 14.311, *df* = 2, *p* = 0.001), but this finding should be interpreted cautiously because some strata were represented by one or two studies and the overall evidence family had extreme heterogeneity.

Age subgroup analyses did not show robust effect modification. For vitamin D supplementation, there was no evidence of differential effects by age group (*Q*-between = 0.524, *df* = 1, *p* = 0.469). For vitamin D status and vitamin B_12_ status, age tests were borderline but did not meet conventional statistical thresholds (*Q*-between = 3.747, *df* = 1, *p* = 0.053; and *Q*-between = 3.372, *df* = 1, *p* = 0.066, respectively). These results suggest that age-related variability may deserve further study, but the present dataset does not support firm age-specific conclusions.

Overall, Phase 2 confirmed that heterogeneity was not adequately resolved by broad phenotype-informed or age subgrouping. The subgroup findings are therefore useful for hypothesis generation and for designing future biomarker-enriched studies, but they should not be interpreted as confirmatory mechanistic evidence.

#### 3.2.3. Meta-Regression and Covariate Adjustment (Phase 3)

Phase 3 examined whether prespecified study-level moderators and quality/certainty indicators explained part of the residual heterogeneity observed in Phase 1 and Phase 2. Meta-regression was conducted only in evidence families meeting the *k* ≥ 10 threshold and was constrained to a parsimonious one-at-a-time framework to reduce overfitting. Candidate moderators included phenotype-informed subgroup category, age stratum, publication year, risk-of-bias or quality indicators, and GRADE certainty, depending on design and data availability.

For vitamin D supplementation, neither phenotype-informed subgroup category nor age explained meaningful variability in effect estimates (*QM* = 0.519, *df* = 2, *p* = 0.771; and *QM* = 0.524, *df* = 1, *p* = 0.469, respectively). The age coefficient for children/adolescents versus adults was small and not supported under Hartung-Knapp-type inference (*β* = −0.328, *p* = 0.499). Publication year, RoB 2 ordinal rating, and GRADE certainty also showed no evidence of systematic moderation.

For vitamin D status, phenotype-informed subgroup category was not associated with differential effects (*QM* = 1.774, *df* = 2, *p* = 0.412). The age moderator test was borderline but did not meet conventional thresholds (*QM* = 3.747, *df* = 1, *p* = 0.053), and the corresponding Hartung-Knapp inference did not support a stable age effect (*β* = 0.528, *p* = 0.110). Publication year, GRADE certainty, and NOS score similarly did not explain the substantial residual heterogeneity.

For vitamin B_12_ status, phenotype-informed subgroup category showed a statistically detectable omnibus moderator signal (*QM* = 14.311, *df* = 2, *p* = 0.001), consistent with Phase 2. However, this result must be interpreted cautiously because the evidence family included only ten studies, some subgroup strata were too sparse for pooling, and overall heterogeneity remained extreme. The age moderator was borderline (*QM* = 3.372, *df* = 1, *p* = 0.066), and the age coefficient was large but imprecise under Hartung-Knapp-type inference (*β* = −2.041, *p* = 0.107).

Overall, Phase 3 did not identify robust, clinically generalizable moderators that could fully explain the heterogeneity observed across the main evidence streams. The findings reinforce the conclusion that pooled estimates should not be treated as stable average effects across psychiatric populations. Instead, heterogeneity appears to be a defining feature of the evidence base and supports the need for future preregistered, deficiency-enriched, biomarker-stratified trials and observational studies with harmonized exposure definitions, comorbidity assessment, and psychiatric endpoints.

## 4. Discussion

### 4.1. Principal Findings and Robustness of the Evidence

This systematic review and exploratory meta-analysis synthesized evidence on vitamin D and vitamin B_12_ in psychiatric disorders through prespecified nutrient- and comparison-specific evidence streams. The central finding is the consistent heterogeneity and limited robustness of the available evidence, rather than the presence of a uniform psychiatric effect. Across the three primary evidence streams eligible for global pooling—vitamin D supplementation, vitamin D status, and vitamin B_12_ status—pooled point estimates were inverse, but all were accompanied by substantial or extreme heterogeneity, prediction intervals crossing or spanning the null, and sensitivity findings that limited clinical portability.

For vitamin D supplementation, the initial pooled estimate suggested an inverse average association on the harmonized OR scale (OR = 0.439, 95% CI 0.272–0.710), and the supplementation-family SMD estimate was directionally consistent with symptom improvement (SMD = −0.454, 95% CI −0.718 to −0.189; Table S14, Supplementary Materials). However, this signal was not robust as a generalizable intervention effect. Heterogeneity was high (*I*^2^ = 84.2%), Egger’s test indicated small-study effects, and trim-and-fill adjustment attenuated the pooled estimate to the null (adjusted OR = 0.88, 95% CI 0.48–1.63), with a very wide adjusted prediction interval (0.05–15.01). These findings indicate that the average estimate cannot be interpreted as evidence supporting routine vitamin D supplementation across unselected psychiatric populations.

For vitamin D status, the primary observational pooled estimate was also inverse (OR = 0.615, 95% CI 0.424–0.890), but heterogeneity was substantial (*I*^2^ = 83.3%), and trim-and-fill adjustment attenuated the estimate to the null (adjusted OR = 0.90, 95% CI 0.54–1.49). Therefore, the vitamin D status result is best interpreted as a heterogeneous observational association rather than as evidence of causal protection. Higher vitamin D status may partly reflect baseline health, lifestyle, seasonality, metabolic status, sun exposure, diet, or comorbidity profiles rather than a direct psychiatric protective effect.

For vitamin B_12_ status, the pooled association was inverse and statistically significant (OR = 0.310, 95% CI 0.115–0.834), but heterogeneity was extreme (*I*^2^ = 94.8%), and the prediction interval was very wide (0.013–7.518). This finding indicates that the pooled estimate is not clinically portable and should not be interpreted as a generalizable B_12_-related psychiatric effect. Instead, it should be interpreted as a context-dependent, deficiency-linked association signal that may be clinically meaningful in selected patients, particularly when B_12_ deficiency is plausible or when psychiatric symptoms overlap with reversible neuropsychiatric manifestations of deficiency.

Placebo-controlled vitamin B_12_ supplementation evidence was sparse (k = 4) and null on both the harmonized OR scale (OR = 0.989, 95% CI 0.563–1.737) and the SMD scale (SMD = −0.006, 95% CI −0.317 to 0.304; Table S14, Supplementary Materials). Because this evidence family did not meet the prespecified *k* ≥ 10 threshold, it cannot support stable pooled inference, subgroup analysis, meta-regression, or small-study-effect assessment. Its relocation to the Supplementary Materials reflects its exploratory status and avoids visual or inferential parity with the adequately powered evidence streams.

### 4.2. Data-Supported Findings Versus Biological Interpretation

Three data-supported conclusions emerge from the synthesis. First, vitamin D supplementation and vitamin D status show initial inverse average estimates, but these are not robust to heterogeneity and small-study-effect sensitivity analyses. Second, vitamin B_12_ status shows a stronger inverse point estimate, but with extreme heterogeneity and a very wide prediction interval, making the average effect non-portable across clinical contexts. Third, placebo-controlled vitamin B_12_ supplementation evidence is too sparse and too close to the null to support routine adjunctive use in unselected psychiatric populations.

These data-supported findings must be separated from biological plausibility. Vitamin D has plausible neuroimmune, neuroglial, serotonergic, stress-regulatory, and neurodevelopmental relevance [16,17,18,19,20,21,22,23,24,25,26,27], while vitamin B_12_ participates in one-carbon metabolism, methylation, homocysteine regulation, redox balance, and neurochemical pathways [1,2,3,4,5,6,7,8,9,10,11,12,13,14,15]. These mechanisms justify targeted hypotheses, deficiency assessment, and biologically enriched future study designs. They do not, however, demonstrate that vitamin D or vitamin B_12_ supplementation produces uniform psychiatric benefits across diagnoses, age groups, or baseline nutrient states.

The phenotype-informed serotonergic, dopaminergic, and mixed/other subgroup categories should therefore be interpreted as exploratory descriptors of clinical and biological plausibility, not as direct mechanistic evidence. Most primary studies did not measure serotonergic or dopaminergic pathway biomarkers, astroglial activity, glymphatic function, neuroimmune signaling, or methylation indices in a way that would allow pathway-level causal inference. The subgroup analyses identify possible sources of heterogeneity and support hypothesis generation, but they should not be used to claim demonstrated monoaminergic or pathway-specific effects.

This distinction is particularly important for the vitamin B_12_ status evidence. The significant phenotype-informed moderator signal for B_12_ status suggests that effect estimates differ across broad clinical categories, but the strata were sparse and overall heterogeneity remained extreme. Therefore, the moderator result should be treated as a signal that clinical context matters, not as confirmation of a specific serotonergic, dopaminergic, or mixed biological mechanism.

### 4.3. Clinical Interpretation and Translational Implications

From a clinical perspective, the findings support targeted assessment rather than routine supplementation. For vitamin B_12_, assessment is clinically plausible when patients present with depressive, cognitive, psychotic, fatigue-related, or nonspecific neuropsychiatric symptoms that may overlap with deficiency states, especially in older adults, restrictive diets, malabsorption risk, metformin or proton-pump inhibitor exposure, alcohol use, pregnancy/postpartum contexts, or other clinically relevant risk settings. In borderline cases, serum B_12_ alone may be insufficient, and functional markers such as methylmalonic acid and/or homocysteine may improve interpretability when available [1,2,3,4,5,6,7].

For vitamin D, the findings support clinical interest in deficiency detection and correction for general medical reasons and for hypothesis-driven psychiatric research, but not routine adjunctive supplementation as a psychiatric intervention for unselected patients. The attenuation of both supplementation and status estimates after trim-and-fill adjustment indicates that the evidence is not sufficiently robust for diagnosis-agnostic psychiatric recommendations. Where vitamin D supplementation is studied or considered, baseline deficiency, dose, duration, seasonality, inflammation, metabolic burden, and psychiatric endpoint selection should be explicitly incorporated into decision-making and trial design.

Metabolic and endocrine comorbidity may confound both nutrient status and psychiatric outcomes. Obesity, insulin resistance, endocrine disruption, inflammatory burden, altered diet, reduced outdoor activity, and medication-related metabolic effects can influence vitamin D or B_12_ status while also shaping psychiatric severity, functioning, and treatment response. This issue is especially relevant in severe mental illness, where metabolic and endocrine disorders frequently co-occur and complicate clinical interpretation [26]. Accordingly, nutrient status should not be interpreted as an isolated causal exposure when metabolic, endocrine, inflammatory, and lifestyle factors are insufficiently measured.

A precision-oriented interpretation is therefore more consistent with the evidence than a broad supplementation-oriented interpretation. Nutrient assessment may be clinically useful when deficiency is plausible, when symptoms are compatible with reversible deficiency states, or when comorbid medical risk justifies testing. However, routine adjunctive supplementation across broad psychiatric diagnoses is not supported by the present evidence. The available findings support targeted deficiency assessment, correction of confirmed or strongly suspected deficiency according to standard clinical care, and future trials designed around biologically plausible responder subgroups.

### 4.4. Limitations and Research Priorities

Several limitations constrain causal interpretation and clinical translation. First, the review was not prospectively registered in PROSPERO or an equivalent registry. Although protocol-equivalent documentation, search strategies, screening decisions, exclusion reasons, risk-of-bias assessments, certainty judgments, and analytic outputs were maintained in the Supplementary Materials, the absence of prospective registration remains a methodological limitation. Second, Google Scholar was used as a supplementary retrieval and citation-chasing source; although this improved sensitivity for difficult-to-index records, Google Scholar has limited transparency, ranking instability, and reduced reproducibility compared with conventional bibliographic databases.

Third, the included studies differed substantially in baseline deficiency enrichment, assay platforms, threshold definitions, psychiatric phenotypes, outcome instruments, follow-up durations, supplementation dose, comparator structure, and covariate adjustment. These differences reduce cross-study comparability and help explain why prediction intervals were wide even when pooled mean effects were statistically significant. Fourth, observational status-outcome estimates remain vulnerable to residual confounding, including socioeconomic patterning, diet, comorbidity burden, health-seeking behavior, seasonality, sun exposure, inflammatory status, and confounding by indication for supplementation.

Fifth, the conversion of continuous psychiatric symptom outcomes to the OR scale introduces approximation error. The Chinn-type transformation supports cross-family harmonization, but it relies on distributional assumptions that may not hold for symptom-rating scales. Therefore, OR magnitudes derived from continuous outcomes should be interpreted qualitatively and comparatively rather than as direct clinical risk estimates. For supplementation evidence families, the SMD scale is more clinically interpretable and is reported in Table S14 of the Supplementary Materials.

Sixth, phenotype-informed subgrouping remains a coarse study-level approximation. The serotonergic, dopaminergic, and mixed/other categories were based mainly on clinical phenotype and biomarker/intervention context, not on direct pathway measurement. Sparse strata, high heterogeneity, and wide confidence intervals limited subgroup inference, especially for the dopaminergic vitamin D supplementation stratum and for vitamin B_12_ status strata outside the serotonergic category. These analyses therefore generate hypotheses but do not demonstrate pathway mechanisms.

Future trials should be preregistered, adequately powered, and deficiency-enriched. They should report baseline vitamin D and B_12_ status using standardized assay methods, include functional B_12_ markers when feasible, define psychiatric endpoints prospectively, harmonize outcome timing, and stratify or randomize by baseline deficiency. Trials should avoid enrolling broad, unselected psychiatric populations when the biological hypothesis concerns deficiency correction, because such designs may dilute treatment effects and obscure responder subgroups.

Future observational studies should improve the measurement of metabolic, endocrine, inflammatory, lifestyle, dietary, medication-related, and socioeconomic covariates. Individual-participant data meta-analyses and large harmonized prospective cohorts may be especially useful for disentangling nutrient status from comorbidity burden and baseline health profiles. Designs capable of testing nutrient-phenotype, nutrient-deficiency, and vitamin D × vitamin B_12_ interaction structures are required before claims of synergy or pathway-specific effects can move beyond mechanistic plausibility.

## 5. Conclusions

This exploratory systematic review and meta-analysis indicate that evidence linking vitamin D and vitamin B_12_ to psychiatric outcomes remains heterogeneous, context-dependent, and insufficient to support routine adjunctive supplementation across unselected psychiatric populations. Vitamin D supplementation and vitamin D status showed initial inverse pooled estimates, but both evidence streams were characterized by substantial heterogeneity, prediction intervals crossing unity, small-study-effect concerns, and attenuation to the null after trim-and-fill adjustment. Vitamin B_12_ status showed an inverse association with psychiatric outcomes, but extreme heterogeneity and a very wide prediction interval limit the clinical portability of the pooled estimate and support interpretation as a context-dependent, deficiency-linked association signal rather than as a generalizable effect. Placebo-controlled vitamin B_12_ supplementation evidence was sparse and null, allowing only exploratory interpretation. Overall, the findings support targeted assessment and correction of reversible deficiency in clinically selected patients, particularly when B_12_ deficiency is plausible or when vitamin D deficiency is relevant for broader medical care, but they do not justify diagnosi-sagnostic supplementation recommendations. Future research should prioritize preregistered, adequately powered, deficiency-enriched, and biomarker-stratified studies with harmonized psychiatric endpoints, standardized nutrien-status assessment, careful measurement of metabolic and endocrine comorbidity, and explicit testing of clinically plausible effect modifiers.

## Figures and Tables

**Table 1:** Study-level effect estimates for vitamin D supplementation versus placebo/usual care on psychiatric outcomes.

No.	Study	logOR	SE	Weight (%)	OR (95% CI)
1	Elshorbagy et al. (2018) [53]	−3.2511	0.6908	4.4	0.039 (0.010; 0.150)
2	Rouhi et al. (2018) [80]	−1.9144	0.4338	6.1	0.147 (0.063; 0.345)
3	Vellekkatt et al. (2020) [86]	−1.7869	0.5681	5.2	0.167 (0.055; 0.510)
4	Vaziri et al. (2016) [85]	−1.3883	0.3210	6.9	0.249 (0.133; 0.468)
5	Naeini et al. (2019) [73]	−1.3456	0.4443	6.0	0.260 (0.109; 0.622)
6	Mohammadpour et al. (2018) [72]	−1.2617	0.5111	5.6	0.283 (0.104; 0.771)
7	Hemamy et al. (2021) [60]	−1.2107	0.4641	5.9	0.298 (0.120; 0.740)
8	Kaviani et al. (2022) [63]	−1.1533	0.4934	5.7	0.316 (0.120; 0.830)
9	Ghaderi et al. (2017) [59]	−0.8792	0.4466	6.0	0.415 (0.173; 0.996)
10	Omidian et al. (2019) [76]	−0.7245	0.4515	6.0	0.485 (0.200; 1.174)
11	Dehbokri et al. (2019) [51]	−0.5453	0.3713	6.5	0.580 (0.280; 1.200)
12	Mazahery et al. (2019) [70]	−0.4559	0.6174	4.9	0.634 (0.189; 2.126)
13	Okereke et al. (2020) [75]	−0.0265	0.0575	8.0	0.974 (0.870; 1.090)
14	Rahman et al. (2023) [79]	−0.0142	0.0465	8.0	0.986 (0.900; 1.080)
15	Krivoy et al. (2017) [65]	0.2187	0.5305	5.4	1.245 (0.440; 3.520)
16	Kerley et al. (2017) [64]	0.6346	0.5930	5.0	1.886 (0.590; 6.030)
17	Marsh et al. (2017) [69]	0.6443	0.7085	4.3	1.905 (0.475; 7.637)
Random effects model (IV, 95% CI)				100.00	0.439 (0.272; 0.710)
	Prediction Interval (PI)				(0.082; 2.360)

Notes: logOR denotes the natural logarithm of the odds ratio, SE denotes the standard error of logOR, and OR denotes the odds ratio after back-transformation. OR < 1 indicates a more favorable psychiatric profile in the vitamin D supplementation group under the harmonized direction of effect. This table reports the study-level inputs used for the initial random-effects synthesis; interpretation of the pooled estimate requires consideration of heterogeneity, prediction intervals, and small-study-effect sensitivity analyses.

**Table 2:** Summary of the initial random-effects meta-analysis of vitamin D supplementation versus placebo/usual care.

Domain	Statistic	Value
Pooled effect	Number of studies (*k*)	17
	Odds Ratio (OR, 95% CI)	0.439 (0.272; 0.710)
	*p*-value (overall effect)	0.0023
Heterogeneity	Cochran’s *Q* test (Chi^2^)	101.30
	Degrees of freedom (*df*)	16
	*p*-value (*Q* test)	<0.0001
	*I*^2^ (95% CI), %	84.2 (76.0; 89.6)
	*τ*^2^ (95% CI)	0.5820 (0.249; 1.957)
	*τ* (95% CI)	0.7629 (0.499; 1.399)
	Higgins’ *H* (95% CI)	2.520 (2.042; 3.100)
Prediction interval (PI)	95% Prediction Interval (OR)	0.082 to 2.360
Publication bias	Egger’s intercept (95% CI)	−2.18 (−3.23 to −1.13)
	*t*-statistic	−4.06
	*p*-value (Egger’s test)	0.001

Notes: OR (Odds Ratio) represents the relative odds of the psychiatric outcome in the vitamin D supplementation group compared with placebo/usual care under the harmonized direction of effect; OR < 1 indicates a more favorable psychiatric profile in the supplementation group. logOR is the natural logarithm of the odds ratio, and SE denotes the standard error of logOR. Cochran’s *Q* assesses whether observed variability across studies exceeds that expected by chance; *df* corresponds to the number of studies minus one. *I*^2^ is reported as a percentage and quantifies the proportion of total variability attributable to between-study heterogeneity rather than sampling error; values in parentheses denote the 95% confidence interval. *τ*^2^ represents the estimated between-study variance of true effects, while *τ* is its square root. Higgins’ *H* statistic is calculated as √(*Q*/*df*) and reflects excess heterogeneity beyond chance. The prediction interval indicates the range in which the true effect of a future comparable study is expected to lie under the random-effects model. Egger’s test evaluates funnel-plot asymmetry as an indicator of possible small-study effects or publication bias. Because heterogeneity was substantial (*I*^2^ = 84.2%), the prediction interval crossed unity (0.082–2.360), and Egger’s test was significant (*p* = 0.001), this table should be interpreted as an initial pooled estimate requiring sensitivity analysis rather than as evidence of a stable generalizable supplementation effect.

**Table 3:** Trim-and-fill-adjusted meta-analytic findings for vitamin D supplementation versus placebo/usual care.

Domain	Statistic	Value
Trim-and-Fill (sensitivity)	Number of imputed (“filled”) studies	7
	Adjusted number of studies (*k*)	24
Adjusted pooled effect	Adjusted Odds Ratio (OR, 95% CI)	0.88 (0.48; 1.63)
	*p*-value (overall effect)	0.6828
Adjusted heterogeneity	Cochran’s *Q* test (Chi^2^)	193.09
	Degrees of freedom (*df*)	23
	*p*-value (*Q* test)	<0.0001
	*I*^2^ (95% CI), %	88.1 (83.7; 90.5)
	*τ*^2^ (95% CI)	1.7889 (0.976; 3.773)
	*τ* (95% CI)	1.3375 (0.988; 1.942)
	Higgins’ *H* statistic (95% CI)	2.897 (2.474; 3.247)
Adjusted prediction interval (PI)	95% Prediction Interval (OR)	0.05 to 15.01

Notes: The trim-and-fill procedure is an exploratory sensitivity analysis used to assess the potential impact of funnel-plot asymmetry and small-study effects. Adjusted *k* represents the observed studies plus imputed studies. OR < 1 indicates a more favorable psychiatric profile in the vitamin D supplementation group. The attenuation of the pooled estimate to the null after imputation, combined with high residual heterogeneity and a very wide prediction interval, indicates that the initial pooled estimate is not robust as a generalizable intervention effect.

**Table 4:** Study-level effect estimates for vitamin D status versus psychiatric outcomes.

No.	Study	logOR	SE	Weight (%)	OR (95% CI)
1	Petruzzelli et al. (2020) [78]	−2.3454	0.8531	2.60	0.096 (0.018; 0.510)
2	Fabrazzo et al. (2022) [58]	−1.6008	0.3095	7.70	0.202 (0.110; 0.370)
3	Okasha et al. (2020) [74]	−1.5299	0.7687	3.10	0.217 (0.048; 0.977)
4	Shahini et al. (2022) [81]	−1.1597	1.1807	1.60	0.314 (0.031; 3.172)
5	Van der Leeuw et al. (2020) [84]	−0.7786	0.2020	9.20	0.459 (0.309; 0.682)
6	Yee et al. (2016) [88]	−0.7649	0.4598	5.70	0.465 (0.189; 1.146)
7	Boerman et al. (2016) [48]	−0.5813	0.2645	8.40	0.559 (0.333; 0.939)
8	Endres et al. (2016) [54]	−0.4476	0.2022	9.20	0.639 (0.430; 0.950)
9	Sourander et al. (2021) [82]	−0.3221	0.1049	10.40	0.725 (0.590; 0.890)
10	Terock et al. (2020) [83]	−0.0413	0.0159	10.90	0.960 (0.930; 0.990)
11	Madley-Dowd et al. (2022) [68]	−0.0235	0.0417	10.80	0.977 (0.900; 1.060)
12	Ali et al. (2019) [44]	0.0571	0.0553	10.70	1.059 (0.950; 1.180)
13	Libuda et al. (2020) [67]	0.0360	0.1645	9.70	1.037 (0.751; 1.431)
Random effects model (IV, 95% CI)				100.00	0.615 (0.424; 0.890)
Prediction Interval (PI)					(0.203; 1.863)

Notes: logOR denotes the natural logarithm of the odds ratio, SE denotes the standard error of logOR, and OR denotes the odds ratio after back-transformation. OR < 1 indicates a more favorable psychiatric profile in the higher-status or lower-deficiency group according to the harmonized direction of effect. These study-level observational estimates should be interpreted as association evidence rather than as evidence of supplementation benefit or causal protection.

**Table 5:** Summary of the initial random-effects meta-analysis of vitamin D status versus psychiatric outcomes.

Domain	Statistic	Value
Pooled effect	Number of studies (*k*)	13
	Odds Ratio (OR, 95% CI)	0.615 (0.424; 0.890)
	*p*-value (overall effect)	0.0142
Heterogeneity	Cochran’s *Q* test (Chi^2^)	71.73
	Degrees of freedom (*df*)	12
	*p*-value (*Q* test)	<0.0001
	*I*^2^ (95% CI), %	83.3 (72.7; 89.7)
	*τ*^2^ (95% CI)	0.2336 (0.090; 11.65)
	*τ* (95% CI)	0.4830 (0.300; 1.079)
	Higgins’ *H* statistic (95% CI)	2.4500 (1.916; 3.120)
Prediction interval (PI)	95% Prediction Interval (OR)	0.203 to 1.863
Publication bias	Egger’s intercept (95% CI)	−2.09 (−3.220 to −0.960)
	*t*-statistic	−3.627
	*p*-value (Egger’s test)	0.004

Notes: OR (Odds Ratio) represents the relative odds of the psychiatric outcome in the higher vitamin D status group, lower-deficiency group, or corresponding favorable-status contrast according to each study’s design and the harmonized direction of effect. OR < 1 indicates a more favorable psychiatric profile associated with higher vitamin D status. Because this evidence stream is observational, the pooled estimate should be interpreted as an association rather than as evidence of causal protection. Substantial heterogeneity and a prediction interval crossing unity indicate limited clinical portability and support the need for sensitivity analyses.

**Table 6:** Trim-and-fill-adjusted meta-analytic findings for vitamin D status versus psychiatric outcomes.

Domain	Statistic	Value
Trim-and-Fill (sensitivity)	Number of imputed (“filled”) studies	6
	Adjusted number of studies (*k*)	19
Adjusted pooled effect	Adjusted Odds Ratio (OR, 95% CI)	0.90 (0.54; 1.49)
	*p*-value (overall effect)	0.6618
Adjusted heterogeneity	Cochran’s *Q* test (Chi^2^)	125.29
	Degrees of freedom (*df*)	18
	*p*-value (*Q* test)	<0.0001
	*I*^2^ (95% CI), %	85.6 (79.5; 89.6)
	*τ*^2^ (95% CI)	0.7240 (0.332; 8.011)
	*τ* (95% CI)	0.851 (0.576; 2.830)
	Higgins’ *H* statistic (95% CI)	2.638 (2.209; 3.096)
Adjusted prediction interval (PI)	95% Prediction Interval (OR)	0.14 to 5.70

Notes: The trim-and-fill procedure is an exploratory sensitivity analysis intended to assess the potential influence of funnel-plot asymmetry and small-study effects. Adjusted *k* represents the observed studies plus imputed studies. OR < 1 indicates a more favorable psychiatric profile associated with higher vitamin D status or lower-deficiency contrasts. The attenuation of the pooled estimate to the null after imputation indicates that the primary observational association is not robust to small-study-effect adjustment.

**Table 7:** Study-level effect estimates for vitamin B_12_ status versus psychiatric outcomes.

No.	Study	logOR	SE	Weight (%)	OR (95% CI)
1	Altun et al. (2018) [46]	−6.1030	0.4106	9.70	0.002 (0.001; 0.005)
2	Yektaş et al. (2019) [89]	−2.6191	0.4528	9.60	0.073 (0.030; 0.177)
3	Yazici et al. (2019) [87]	−1.8649	0.3372	10.00	0.160 (0.080; 0.300)
4	Esnafoğlu & Yaman (2017) [57]	−1.8134	0.4316	9.60	0.160 (0.070; 0.380)
5	Esnafoglu & Ozturan (2020) [56]	−1.5141	0.3537	9.9	0.220 (0.110; 0.440)
6	Dhiman et al. (2021) [52]	−0.6667	0.2932	10.10	0.514 (0.289; 0.912)
7	Erensoy (2020) [55]	−0.6169	0.3726	9.90	0.540 (0.260; 1.120)
8	Anmella et al. (2025) [47]	−0.2005	0.0653	10.60	0.820 (0.720; 0.930)
9	Laird et al. (2023) [66]	0.4149	0.2066	10.40	1.510 (1.010; 2.270)
10	Huang et al. (2018) [62]	0.6372	0.2538	10.20	1.890 (1.150; 3.110)
Random effects model (IV, 95% CI)				100.00	0.310 (0.115; 0.834)
Prediction Interval (PI)					(0.013; 7.518)

Notes: logOR denotes the natural logarithm of the odds ratio, SE denotes the standard error of logOR, and OR denotes the odds ratio after back-transformation. OR < 1 indicates a more favorable psychiatric profile in the higher-status or lower-deficiency group according to the harmonized direction of effect. Because the included studies were observational and highly heterogeneous, these estimates should be interpreted as association evidence rather than as evidence of a stable or causal B_12_ effect.

**Table 8:** Summary of the random-effects meta-analysis of vitamin B_12_ status versus psychiatric outcomes.

Domain	Statistic	Value
Pooled effect	Number of studies (*k*)	10
	Odds Ratio (OR, 95% CI)	0.310 (0.115; 0.834)
	*p*-value (overall effect)	0.0253
Heterogeneity	Cochran’s *Q* test (Chi^2^)	174.01
	Degrees of freedom (*df*)	9
	*p*-value (*Q* test)	<0.0001
	*I*^2^ (95% CI), %	94.8 (92.3; 96.5)
	*τ*^2^ (95% CI)	1.7957 (0.7910; 6.2860)
	*τ* (95% CI)	1.340 (0.889; 2.507)
	Higgins’ *H* statistic (95% CI)	4.400 (3.601; 5.369)
Prediction interval (PI)	95% Prediction Interval (OR)	0.013 to 7.518
Publication bias	Egger’s intercept (95% CI)	−4.71 (−9.46 to 0.04)
	*t*-statistic	−1.945
	*p*-value	0.088

Notes: OR (Odds Ratio) represents the relative odds of the psychiatric outcome according to the harmonized vitamin B_12_ status contrast. OR < 1 indicates a more favorable psychiatric profile in the higher-status or lower-deficiency group. The pooled estimate should be interpreted with caution because heterogeneity was extreme and the prediction interval was very wide. These findings indicate a context-dependent association signal rather than a clinically portable mean effect.

## Data Availability

No new primary data were generated in this study. All analyses were based on extracted and harmonized study-level data derived from published studies. The harmonized study-level dataset, including effect sizes and covariates supporting the findings of this systematic review and meta-analysis, is provided in the Supplementary Materials.

## Supplementary Materials

The following supporting information can be downloaded at: https://www.mdpi.com/article/10.3390/diseases14050167/s1, Table S1. Database-specific search strategies used in the systematic review; Table S2. Synopsis of studies contributing to the qualitative and/or quantitative evidence synthesis; Table S3. Studies excluded from the qualitative and/or quantitative synthesis: rationale aligned with prespecified eligibility criteria; Table S4. Overview of included studies and consolidated key characteristics; Table S5. Risk-of-Bias (RoB 2) appraisal for randomized controlled trials and Certainty-of-Evidence ratings under the GRADE approach; Table S6. Methodological quality appraisal of observational studies using the Newcastle-Ottawa Scale (NOS) and Certainty-of-Evidence judgments under the GRADE framework; Table S7. Comparative random-effects meta-analysis results across nutrient- and comparison-specific evidence streams; Table S8. Phase 2 study-level effect estimates used for phenotype-informed exploratory subgroup and age-moderator analyses; Table S9. Phase 2 random-effects subgroup meta-analyses by phenotype-informed exploratory subgroup; Table S10. Phase 2 tests for subgroup differences using phenotype-informed exploratory subgroup as a categorical moderator (Q-between); Table S11. Phase 2 tests for subgroup differences using age subgroup as a categorical moderator (Adults vs. Children/Adolescents; Q-between); Table S12. Phase 3 study-level covariates, phenotype-informed exploratory subgroup assignments, and quality/certainty assessments used for meta-regression and covariate adjustment; Table S13. Phase 3 meta-regression and covariate-adjustment summary for phenotype-informed exploratory subgroup, age, and study-level covariates; Table S14. SMD-based interpretability analysis for supplementation evidence streams; Table S15. Exploratory study-level effect estimates for vitamin B12 supplementation versus placebo/usual care; Table S16. Exploratory random-effects meta-analysis of vitamin B12 supplementation versus placebo/usual care.
