# Supplementary material for: Vitamin D and Vitamin B12 in Psychiatric Disorders: An Exploratory Systematic Review and Meta-Analysis of Nutrient-Specific Status and Supplementation Evidence
Source: Diseases. 2026 May 10;14(5):167. doi: 10.3390/diseases14050167 (PMC13206110; doi:10.3390/diseases14050167)
Supplement: Supplementary file 1 [file diseases-14-00167-s001.zip › diseases-4288571-supplementary.pdf]

Supplementary Materials for:

" Vitamin D and Vitamin B_{12} in Psychiatric Disorders: An Exploratory Systematic Review and Meta–analysis of Nutrient–Specific Status and Supplementation Evidence"

Protocol Deviations and Amendments

The review followed a prespecified methodological protocol finalized before study screening and quantitative synthesis. Eligibility criteria, search strategy, study-selection workflow, data-extraction rules, effect-size hierarchy, risk-of-bias assessment, certainty-of-evidence appraisal, subgroup definitions, sensitivity analyses, and statistical model specifications were applied consistently across the evidence base. No deviations from the prespecified protocol occurred after screening commenced. No changes were made to eligibility criteria, study-selection rules, extracted effect-size hierarchy, harmonization procedures, risk-of-bias appraisal, certainty assessment, random-effects model specification, REML τ^2 estimation, Hartung–Knapp inference, or prespecified thresholds for primary pooling and moderator analyses.

All procedures and decisions are documented in the Supplementary Materials. Database-specific search strategies are reported in Table S1; the included-study synopsis is provided in Table S2; full-text exclusions with explicit reasons are documented in Table S3; consolidated study characteristics and extraction structure are provided in Table S4; methodological quality, risk-of-bias, and certainty judgments are presented in Tables S5–S6; Phase 1 comparative synthesis, Phase 2 subgroup analyses, Phase 3 meta-regression outputs, SMD-based supplementation estimates, and exploratory vitamin B_{12} supplementation results are provided in Tables S7–S16.

Table S1. Database–specific search strategies used in the systematic review.

Search ID	Access Interface	Core Database	Resource Types Included	Search Strategy and Terms
**1**	NCBI PubMed	MEDLINE/PubMed records	Peer–reviewed human studies (RCTs and observational designs); biomarker/mechanistic reports with extractable psychiatric outcomes	• MeSH + keyword Boolean core: (("Vitamin D"[Mesh] OR "Cholecalciferol"[Mesh] OR calcifediol[tiab] OR calcidiol[tiab] OR "25–hydroxyvitamin D"[tiab] OR "25(OH)D"[tiab] OR vitamin D[tiab] OR cholecalciferol[tiab]) OR ("Vitamin B $_{12}$"[Mesh] OR cobalamin[tiab] OR methylcobalamin[tiab] OR vitamin B12[tiab])) AND ("Depressive Disorder"[Mesh] OR "Depression"[Mesh] OR "Anxiety Disorders"[Mesh] OR "Bipolar Disorder"[Mesh] OR "Schizophrenia"[Mesh] OR "Psychotic Disorders"[Mesh] OR "Cognition Disorders"[Mesh] OR "Memory Disorders"[Mesh] OR depress*[tiab] OR anxiety[tiab] OR bipolar[tiab] OR schizophren*[tiab] OR psychosis[tiab] OR "psychiatric disorder*"[tiab] OR cognition[tiab] OR memory[tiab]).<br>• Design filter (broad): (random*[tiab] OR trial[tiab] OR placebo[tiab] OR cohort[tiab] OR "case–control"[tiab] OR cross–sectional[tiab] OR observational[tiab]).<br>• Limits: Humans; 2016–2025. Optional contextual refinement (not required for eligibility): AND (serotonin[tiab] OR dopamine[tiab] OR TPH2[tiab] OR "tryptophan hydroxylase"[tiab] OR serotonerg*[tiab] OR dopaminerg*[tiab]).
**2**	Ovid / EBSCOhost	APA PsycInfo	Peer–reviewed psychology/psychiatry journals; clinical/observational studies; biomarker reports with psychiatric outcomes	• Thesaurus + keywords core: (DE "Depression" OR DE "Major Depression" OR DE "Anxiety Disorders" OR DE "Bipolar Disorder" OR DE "Schizophrenia" OR DE "Psychotic Disorders" OR DE "Cognition" OR DE "Memory") AND ((DE "Vitamin D" OR TX("vitamin D" OR cholecalciferol OR calcifediol OR "25–hydroxyvitamin D" OR "25(OH)D")) OR (DE "Vitamin B_{12}" OR TX("vitamin B_{12}" OR cobalamin OR methylcobalamin))).<br>• Limits: Peer–reviewed; Human; Year 2016–2025. Optional contextual refinement: AND (DE "Serotonin" OR DE "Dopamine"

				OR TX(TPH2 OR "tryptophan hydroxylase" OR serotonerg* OR dopaminerg*)).<br>• Methodology filters (when available): Clinical Trial/Intervention; Longitudinal/Prospective; Case–Control; Cross–Sectional.
**3**	Cochrane Library	CENTRAL (Cochrane Central Register of Controlled Trials)	Randomized trials and controlled clinical trials	• CENTRAL core (Title/Abstract/Keywords): (depress* OR anxiety OR bipolar OR schizophrenia OR psychosis OR "psychiatric disorder*" OR cognition OR memory) AND ("vitamin D" OR cholecalciferol OR calcifediol OR "25–hydroxyvitamin D" OR "25(OH)D" OR "vitamin B_{12}" OR cobalamin OR methylcobalamin).<br>• Limits/filters: Trials (CENTRAL); 2016–2025; human clinical trials. Optional contextual refinement: (serotonin OR dopamine OR TPH2 OR "tryptophan hydroxylase").
**4**	Google Scholar	Aggregated scholarly sources	Journal articles and conference papers; targeted "snowballing" for hard–to–index items	• Two complementary core queries (run separately to avoid false exclusion)**:** (i) ("vitamin D" OR cholecalciferol OR calcifediol OR "25–hydroxyvitamin D" OR "25(OH)D") AND (depression OR depressive OR anxiety OR bipolar OR schizophrenia OR psychosis OR cognition OR memory) AND (trial OR randomized OR placebo OR cohort OR "case–control" OR cross–sectional). (ii) ("vitamin B_{12}" OR cobalamin OR methylcobalamin) AND (depression OR depressive OR anxiety OR bipolar OR schizophrenia OR psychosis OR cognition OR memory) AND (trial OR randomized OR placebo OR cohort OR "case–control" OR cross–sectional).<br>• Date range: 2016–2025; screening by relevance; inspect first ~200 results per query; forward/backward citation chasing for eligible primary studies.<br>• Optional contextual add–on query (separate, not restrictive)**:** AND (serotonin OR dopamine OR TPH2).
**5**	ClinicalTrials.gov	ClinicalTrials.gov registry	Registered interventional studies (completed/ongoing)	• Advanced search fields: Condition/Disease = (Depression OR Depressive Disorder OR Anxiety OR Schizophrenia OR Psychosis OR Bipolar Disorder OR Cognition/Memory).<br>• Intervention/Treatment = ("vitamin D" OR cholecalciferol OR calcifediol OR "vitamin B12" OR cobalamin OR methylcobalamin).<br>• Study Type**:** Interventional.

				• Time window: Start/Completion 2016–2025.<br>• Status: Recruiting/Active/Completed (as applicable).<br>• Optional outcome terms: mood OR depression OR anxiety OR cognition OR serotonin OR dopamine.
**6**	ProQuest (Dissertations & Theses Global / Central)	ProQuest grey literature	Dissertations/theses; reports used for triangulation and to detect publication bias signals	• Free–text Boolean core: (("vitamin D" OR cholecalciferol OR calcifediol OR "25–hydroxyvitamin D" OR "25(OH)D") OR ("vitamin B12" OR cobalamin OR methylcobalamin)) AND (depress* OR anxiety OR bipolar OR schizophrenia OR psychosis OR "psychiatric disorder*" OR cognition OR memory) AND (trial OR random* OR placebo OR cohort OR "case control" OR cross–sectional OR observational OR biomarker).<br>• Limits: 2016–2025; document type Dissertation/Thesis/Report.<br>• Optional contextual refinement: (serotonin OR dopamine OR TPH2 OR serotonerg* OR dopaminerg*).

Notes:

- Search dates: The final search update across all databases and registers was run on 01 March 2026 (coverage: 1 January 2016 to 31 December 2025).
- Mechanistic terms (serotonin/dopamine/TPH2) were used only as optional refinements to support contextual interpretation and exploratory phenotype-informed subgroup coding; they were not required in the core search and were not applied as restrictive eligibility filters.
- No language restriction was applied at the search stage.

Table S2. Synopsis of studies contributing to the qualitative and/or quantitative evidence synthesis.

Record ID	Study	Study Design	Systematic Review	Meta–Analysis	Decision–Making Support
**1**	Ali et al. (2019) [44]	Observational (Prospective cohort study)	Included	Included	Baseline 25–hydroxyvitamin D [25(OH)D] measured prior to incident autism spectrum disorder [ASD] diagnosis; adjusted relative risk reported per 10 nmol/L increase; converted to odds ratio [OR] for harmonization across studies.
**2**	Allott et al. (2019) [45]	RCT (Parallel–group, randomized, double–blind, placebo–controlled trial)	Included	Included	Add–on B–vitamin complex (vitamin B_{12}, vitamin B_6, folic acid) versus placebo in first–episode psychosis; continuous outcomes converted to odds ratio [OR] with 95% confidence interval [CI] using the Chinn method for cross–study harmonization.
**3**	Altun et al. (2018) [46]	Observational (Cross–sectional case–control study)	Included	Included	Case–control comparison of serum biomarkers in autism spectrum disorder (ASD) versus healthy controls; continuous differences converted to odds ratio (OR) with 95% confidence interval (CI) using the Chinn method for cross–study harmonization.
**4**	Anmella et al. (2025) [47]	Observational (Cross–sectional study)	Included	Included	Serum folate (ng/mL) and vitamin B_{12} (pg/mL) were measured on the first working day after admission in 729 child and adolescent psychiatric inpatients at a general hospital. DSM–5 diagnoses were grouped into depressive disorders, schizophrenia spectrum disorders, eating disorders and other categories, and logistic regression models estimated odds ratios for each diagnostic category according to continuous vitamin B12 levels and vitamin B_{12} insufficiency (<300 pg/mL) versus adequate status. For this review, vitamin B_{12} was treated as a status exposure; adjusted odds ratios for depressive disorders and schizophrenia spectrum disorders versus all other diagnoses were extracted and aligned with the "status vs outcome" framework for synthesis.
**5**	Boerman et al. (2016) [48]	Observational (Cross–sectional study)	Included	Included	Serum 25–hydroxyvitamin D [25(OH)D] measured in psychiatric outpatients; prevalence of vitamin D deficiency compared across diagnostic groups; odds ratio [OR] with 95% confidence interval [CI] computed for deficiency (≤12 ng/mL) in schizophrenia/schizoaffective versus bipolar disorder.

**6**	Chen et al. (2024) [49]	RCT (Randomized, double−blind, placebo−controlled clinical trial)	Included	Included	Adjunctive folate (vitamin B_9) 5 mg/day plus vitamin B_{12} (cobalamin) 500 µg/day vs. placebo for 24 weeks in schizophrenia with low serum folate; primary psychopathology outcome on the Positive and Negative Syndrome Scale (PANSS, endpoint−baseline change); continuous effects converted to odds ratio (OR) with 95% confidence interval (CI) via Chinn for cross−study harmonization.
**7**	De Koning et al. (2016) [50]	RCT (Multicenter, randomized, parallel−group, double−blind, placebo−controlled intervention trial)	Included	Included	Large, well−randomized RCT with predefined psychiatric outcomes (GDS−15) and health−related quality of life (SF−12, EQ−5D); reports adjusted logistic regression for depressive symptoms and ANCOVA for EQ−5D; full baseline biomarker panel (homocysteine, vitamin B_{12} status markers) enables mechanistic linkage.
**8**	Dehbokri et al. (2019) [51]	RCT (Randomized, double−blind, placebo−controlled clinical trial)	Included	Included	Adjunctive cholecalciferol 50,000 IU/week for 6 weeks vs. placebo in children with attention−deficit hyperactivity disorder (ADHD); Conners Parent Rating Scale (CPRS) outcomes analyzed overall and by subscales. Primary pooling outcome standardized as OR on the inattention (IA) subscale at 6 weeks.
**9**	Dhiman et al. (2021) [52]	Observational (Cross−sectional case−control analysis using archived plasma samples from a larger cohort study)	Included	Included	Large hospital−based sample; standardized ascertainment of probable postpartum depression (Edinburgh Postnatal Depression Scale, EPDS) and comprehensive biomarker panel (total vitamin B_{12}, holotranscobalamin, methylmalonic acid, homocysteine, 5−methyl tetrahydrofolate, serotonin, S−adenosyl methionine, combined B_{12} score). Multivariable logistic models report adjusted ORs suitable for pooling on the OR scale.
**10**	Elshorbagy et al. (2018) [53]	RCT (Randomized double−blind placebo−controlled trial in vitamin D−deficient ADHD	Included	Included	Double−blind allocation of cholecalciferol 3,000 IU/day versus placebo for 12 weeks as add−on to methylphenidate; standardized symptom scales; post−treatment means and standard deviations available to estimate odds ratios (OR) with 95% confidence intervals (CI); adherence and attrition reported.
**11**	Endres et al. (2016) [54]	Observational (Inpatient cohort vs historical general−population controls)	Included	Included	Provides vitamin D [25−hydroxyvitamin D, 25(OH)D] prevalence in adults with schizophreniform syndromes and autism spectrum disorder, with extractable counts enabling OR estimates vs. national controls; ASD subgroup present; biomarker measured with chemiluminescence immunoassay.

**12**	Erensoy (2020) [55]	Observational (Cross−sectional study)	Included	Included	Serum vitamin B_{12} (pg/mL) and folic acid (ng/mL) were measured after overnight fasting in 150 adult psychiatric outpatients (77 with anxiety disorders, 73 with depressive disorders) at a university neuropsychiatry clinic. Vitamin B_{12} deficiency was defined as <200 pg/mL, and deficiency frequencies were reported separately for anxiety and depression, allowing calculation of an unadjusted odds ratio for depression versus anxiety according to low versus adequate vitamin B_{12} status. Mean B_{12} and folate levels were also compared across diagnostic and severity subgroups, supporting the classification of this study within the vitamin B_{12} status−outcome framework used in the synthesis.
**13**	Esnafoğlu & Yaman (2017) [57]	Observational (Case−control cross−sectional study)	Included	Included	Hospital−based case−control with standardized biomarker assays; clear group definitions; provides compatible continuous outcomes that were harmonized to OR via standard transformation for inclusion in pooled analyses.
**14**	Esnafoglu & Ozturan (2020) [56]	Observational (Case−control, cross−sectional study)	Included	Included	Single−center case−control comparison with standardized serum assays; categorical outcomes (deficiency/elevated status) harmonized to odds ratio (OR) with 95% confidence interval (CI) for pooling.
**15**	Fabrazzo et al. (2022) [58]	Observational (Cross−sectional cohort study)	Included	Included	Direct comparison of psychiatric inpatients versus outpatients with quantified serum 25−hydroxyvitamin D (25(OH)D) and parathyroid hormone (PTH) levels; inpatient versus outpatient contrast allows computation of an odds ratio (OR) for vitamin D deficiency (<20 ng/mL) using reported means and standard deviations (SD) with a normal approximation; provides biomarker−symptom links via Brief Psychiatric Rating Scale (BPRS).
**16**	Ghaderi et al. (2017) [59]	RCT (Randomized, double−blind, placebo−controlled clinical trial)	Included	Included	Randomized, double−blind, placebo−controlled clinical trial among adults aged 25−70 years in maintenance methadone treatment (MMT), testing oral vitamin D3 (cholecalciferol) 50,000 international units (IU) every 2 weeks for 12 weeks versus matching placebo. The trial assessed psychological symptoms using the Beck Depression Inventory (BDI), Beck Anxiety Inventory (BAI) and Pittsburgh Sleep Quality Index (PSQI) together with an extensive metabolic, inflammatory and oxidative stress biomarker panel, and found that vitamin D_3

					supplementation significantly improved Beck Depression Inventory (BDI) and Pittsburgh Sleep Quality Index (PSQI) scores, with supportive evidence for Beck Anxiety Inventory (BAI), while also improving insulin resistance, lipid profile, high−sensitivity C−reactive protein (hs−CRP), total antioxidant capacity (TAC) and glutathione (GSH). The Beck Depression Inventory (BDI) change outcome was harmonized to an odds ratio (OR) via standardized mean difference and Chinn transformation for quantitative synthesis.
**17**	Hemamy et al. (2021) [60]	RCT (Randomized, double−blind, placebo−controlled clinical trial)	Included	Included	Eight−week randomized, double−blind, placebo−controlled trial in 66 children aged 6−12 years with attention−deficit/hyperactivity disorder (ADHD) and documented deficiency of vitamin D and magnesium, all receiving methylphenidate. Participants were allocated to vitamin D (50,000 international units, IU, per week) plus magnesium (6 mg/kg per day) or matching placebos. Mental health was assessed using the strengths and difficulties questionnaire (SDQ), generating emotional, conduct, hyperactivity, peer problems, prosocial, total difficulties, internalizing and externalizing scores. Vitamin D plus magnesium significantly improved several SDQ domains compared with placebo, particularly total difficulties, internalizing and peer problems scores, while also increasing serum 25−hydroxyvitamin D3 [25(OH)D3] and magnesium concentrations. For quantitative synthesis, the adjusted between−group difference in total difficulties score at 8 weeks, derived from analysis of covariance, was converted to a standardized mean difference and then to an odds ratio (OR) using the Chinn transformation, yielding an OR of approximately 3.30 with a 95% confidence interval (CI) from 1.34 to 8.11 for lower total difficulties in the active−supplementation group versus placebo.
**18**	Hendren et al. (2016) [61]	RCT (Randomized, double−blind, placebo−controlled trial)	Included	Included	Eight−week randomized, double−blind, placebo−controlled trial in 57 children aged 3−7 years with autism spectrum disorder (ASD), allocated to subcutaneous methylcobalamin (methyl B12, 75 micrograms per kilogram, μg/kg, every 3 days) or saline placebo injections. The primary outcome was clinician−rated Clinical Global Impressions−Improvement (CGI−I) at 8 weeks; secondary outcomes were parent−rated Aberrant

					Behavior Checklist (ABC) and Social Responsiveness Scale (SRS) scores. Among the 50 children with post−baseline CGI−I data (n = 27 methyl B12; n = 23 placebo), mean CGI−I scores were 2.4 (standard deviation, SD, 0.8) in the methyl B12 group and 3.1 (SD 0.8) in the placebo group, corresponding to a standardized mean difference (Hedges g ≈ 0.86, defined so that positive values favour methyl B12) and an odds ratio (OR) ≈ 4.77, with lowerOR ≈ 1.68 and upperOR ≈ 13.51 for greater global clinical improvement with methyl B12 versus placebo, based on the Chinn transformation. Parent−rated ABC and SRS total and subscale scores did not differ significantly between groups. Laboratory measures of methionine transmethylation/transsulfuration metabolism and glutathione redox status showed that improvements in CGI−I were significantly correlated with increases in methionine, decreases in S−adenosyl−L−homocysteine (SAH) and increases in the S−adenosylmethionine (SAM) to SAH ratio, suggesting that clinical response to methyl B_{12} is linked to improved cellular methylation capacity in a subset of children with autism spectrum disorder (ASD).
**19**	Huang et al. (2018) [62]	Observational (Cross−sectional study)	Included	Included	Cross−sectional analysis of 2,791 U.S. adults aged 20−85 years participating in National Health and Nutrition Examination Survey (NHANES) 2005−2006. Serum concentrations of multiple vitamins (including folate, vitamin B_{12}, 25−hydroxyvitamin D [25(OH)D], vitamin A and carotenoids) were measured using high−performance liquid chromatography (HPLC), standardized liquid chromatography−tandem mass spectrometry (LC−MS/MS) and radioassay. Depressive symptoms were assessed with the 9−item Patient Health Questionnaire (PHQ−9), and depression was defined as PHQ−9 total score ≥ 10. Binary logistic regression with survey weights was used to estimate crude and adjusted odds ratios (ORs) and 95% confidence intervals (CIs) for depression across quartiles of serum vitamin concentrations, adjusting for sex, age, body mass index (BMI), family poverty−income ratio (PIR), race/ethnicity, education, marital status, smoking history, alcohol use and examination season. In fully adjusted models, compared with the lowest quartile (Q1), the highest quartile

					(Q4) of vitamin B_{12} had an OR ≈ 1.89 (95% CI 1.15−3.11) for depression, whereas folate and trans−β−carotene (trans−BC) showed inverse associations and vitamin A showed a positive association; no independent association was observed for 25−hydroxyvitamin D [25(OH)D]. For quantitative synthesis in this review, the primary effect retained is the adjusted association between Q4 versus Q1 of serum vitamin B_{12} and odds of depression.
**20**	Kaviani et al. (2022) [63]	RCT (Randomized, double−blind, clinical trial)	Included	Included	Eight−week double−blind randomized clinical trial in 56 adults aged 18−60 years with mild to moderate depression (Beck Depression Inventory−II (BDI−II) score 13−29) and no other psychiatric disorders, randomized 1:1 to receive oral cholecalciferol 50,000 international units (IU) every 2 weeks or matching placebo capsules, in addition to usual care. Serum 25−hydroxyvitamin D [25(OH)D], intact parathyroid hormone (iPTH), interleukin−1β (IL−1β), interleukin−6 (IL−6), high−sensitivity C−reactive protein (hs−CRP) and depression severity (BDI−II) were assessed at baseline and 8 weeks. The mean change in BDI−II score was −11.75 ± 6.40 points in the vitamin D group versus −3.61 ± 10.40 points in the placebo group (between−group P = 0.003), corresponding to a standardized mean difference (Hedges g ≈ 0.93, defined so that positive values favour vitamin D) and an odds ratio (OR) ≈ 5.40 (lowerOR ≈ 2.01; upperOR ≈ 14.52) for greater improvement in depressive symptoms with vitamin D, based on the Chinn transformation of Hedges g to the log odds ratio. No significant between−group differences were observed for IL−1β, IL−6 or hs−CRP. The trial provides moderate−quality evidence that vitamin D supplementation can substantially reduce depressive symptom severity independently of changes in these selected pro−inflammatory biomarkers.
**21**	Kerley et al. (2017) [64]	RCT (Randomized, parallel−group, double−blind, placebo−controlled trial)	Included	Included	Twenty−week randomized, double−blind, placebo−controlled trial at a paediatric outpatient centre in Dublin (53°N) including 42 children with clinician−diagnosed autism spectrum disorder (ASD), of whom 38 (20 placebo; 18 vitamin D_3) completed follow−up. Participants received either 2,000 international units (IU) vitamin D_3 (cholecalciferol) daily as

					oral drops or matching placebo, administered over the winter period to minimize cutaneous vitamin D synthesis. The primary endpoint was change in the stereotypical behaviour subscale of the Aberrant Behaviour Checklist (ABC) from baseline to 20 weeks; secondary exploratory outcomes included additional ABC subscales, Social Responsiveness Scale (SRS) scores, Developmental Disabilities−Children's Global Assessment Scale (DD−CGAS) domains and biochemical markers of total vitamin D status [25−hydroxyvitamin D, 25(OH)D], inflammation and bone metabolism. Despite a significant increase in serum 25(OH)D in the vitamin D_3 group versus placebo (mean change +27.6 nmol/L vs −1.1 nmol/L), there was no statistically significant between−group difference on the primary ABC stereotypy endpoint. Using the between−group difference in mean change on the ABC stereotypy subscale (improvement of 3.0 points with placebo vs 0.8 points with vitamin D_3) and the trial's assumed standard deviation from the sample size calculation, the standardized mean difference was small−to−moderate in favour of placebo (Hedges g ≈ −0.39 when coded so that positive values favour vitamin D3), corresponding to an odds ratio (OR) ≈ 0.49, with lowerOR ≈ 0.15 and upperOR ≈ 1.58 for greater improvement in stereotypical behaviour with vitamin D_3 versus placebo after Chinn transformation to the log odds ratio.
**22**	Krivoy et al. (2017) [65]	RCT (Randomized, double−blind, placebo−controlled, parallel−arm clinical trial)	Included	Included	Eight−week randomized, double−blind, placebo−controlled clinical trial in 47 adult inpatients and outpatients with DSM−IV−TR schizophrenia maintained on clozapine for at least 18 weeks, with serum 25−hydroxyvitamin D [25(OH)D] < 75 nmol/L and Positive and Negative Syndrome Scale (PANSS) total score > 70 to ensure residual symptoms; participants were allocated 1:1 to weekly vitamin D_3 (14,000 international units (IU) as oral drops, 35 drops) or matching placebo administered under nurse supervision, with assessments every two weeks for psychopathology, mood, cognition and metabolic profile over eight weeks. Primary outcome was change in PANSS total score; secondary outcomes included PANSS subscales, Calgary Depression Scale (CDS), Montreal Cognitive Assessment (MoCA) and metabolic

					parameters (body−mass index, waist circumference, blood pressure, fasting glucose, lipids and glycated haemoglobin). Vitamin D_3 significantly increased serum 25(OH)D (mean +31.4 nmol/L vs −0.4 nmol/L with placebo) but produced no significant between−group difference in PANSS total or subscales. Using the between−group difference in mean change in PANSS total (−8.9 points in the vitamin D group vs −10.0 points in the placebo group, standard deviations 7.3 vs 10.2) the standardized mean difference (Hedges g ≈ −0.12, defined so that positive values favour vitamin D) was transformed via the Chinn method to a log odds ratio, yielding an odds ratio (OR) ≈ 0.80 with lowerOR ≈ 0.28 and upperOR ≈ 2.26 for greater reduction in PANSS total with vitamin D versus placebo. Exploratory analyses suggested a small, non−robust improvement in Montreal Cognitive Assessment (MoCA) scores (effect size ≈ 0.17) driven by attention and delayed recall domains, which lost statistical significance after Bonferroni correction, and no meaningful change in metabolic outcomes.
**23**	Laird et al. (2023) [66]	Observational (Prospective longitudinal cohort with 4−year follow−up)	Included	Included	Four−year longitudinal analysis of 3,849 adults aged ≥ 50 years free of clinically significant depressive symptoms at baseline, with plasma vitamin B_{12} (cobalamin) and folate measured at wave 1 and incident depressive symptoms defined by the 8−item Center for Epidemiological Studies Depression Scale (CES−D−8) score ≥ 9 at waves 2 or 3. Vitamin B12 status categories were < 185 pmol/L (deficient−low), 185−< 258 pmol/L (low−normal), > 258−601 pmol/L (normal, reference) and > 601 pmol/L (high). Fully adjusted logistic regression showed that deficient−low vitamin B_{12} status at baseline was associated with a significantly higher likelihood of incident depressive symptoms over 4 years compared with normal vitamin B_{12} status (odds ratio (OR) = 1.51, 95% confidence interval (CI) 1.01−2.27), robust to adjustment for age, sex, education, body−mass index, chronic disease burden, cardiovascular disease, cognitive impairment, antidepressant use, lifestyle factors and vitamin D status. No independent association was observed between folate status and incident depression. This study provides high−quality observational evidence that low vitamin B_{12}

					status is an independent risk factor for later−onset depressive symptoms in older adults and informs meta−analytic estimation of the effect of deficient versus normal vitamin B_{12} status on depression risk.
**24**	Libuda et al. (2020) [67]	RCT (Randomized, double−blind, placebo−controlled, parallel−group clinical trial)	Included	Included	Provides high−quality randomized evidence that correcting short−term vitamin D deficiency with oral vitamin D_3 2640 international units (IU)/day for 28 days in child and adolescent psychiatric patients leads to a robust increase in serum 25(OH)D concentrations (mean between−group difference at end of study approximately +14 ng/mL) but does not significantly improve self−rated depressive symptoms on the Beck Depression Inventory−II (BDI−II) compared with placebo under concurrent treatment as usual (TAU). Using the reported mean change scores and standard deviations, the standardized mean difference (Hedges g ≈ −0.10, defined so that positive values would indicate greater improvement with vitamin D_3) for BDI−II change was transformed via the Chinn method to an odds ratio (OR) ≈ 0.83, with lowerOR ≈ 0.41 and upperOR ≈ 1.70 for clinically meaningful improvement in self−rated depression, indicating no clear benefit and wide confidence intervals compatible with both modest harm and modest benefit. In contrast, parent−rated depressive symptoms on the Diagnostic System for Mental Disorders in Childhood and Adolescence depression scale [DISYPS−II depression, DISYPS−II depression (DISYPS−DES) parent form] improved significantly more in the vitamin D_3 group (estimated adjusted difference in stanine scores ≈ −0.68, 95% confidence interval −1.23 to −0.13), suggesting a possible rater−dependent effect that informs mechanistic interpretation and future trial design.
**25**	Madley−Dowd et al. (2022) [68]	Observational (Prospective cohort study with additional one−sample Mendelian randomization)	Included	Included	Provides large−cohort, prospective evidence that higher maternal 25−hydroxyvitamin D [25(OH)D] levels during pregnancy are not strongly associated with offspring autism diagnosis or autism−associated trait caseness when confounding and missing data are handled appropriately. Fully adjusted logistic regression models using multiple imputation show an odds ratio (OR) ≈ 0.99 (95% confidence interval 0.93−1.06) per 10 nmol/L increase in maternal

					25(OH)D for autism diagnosis, with similar null findings for social communication difficulties, speech coherence, repetitive behaviour and sociability temperament traits. Mendelian randomization analyses using a maternal genetic risk score for higher 25(OH)D also provide no evidence of a causal effect, though precision is limited. The study supports cautious interpretation of earlier positive findings and is informative for decision−making as high−quality negative evidence in the prenatal vitamin D−autism literature.
**26**	Marsh et al. (2017) [69]	RCT (Randomized, double−blind, placebo−controlled trial)	Included	Included	Provides randomized evidence that high−dose vitamin D3 supplementation does not confer additional short−term antidepressant benefit in bipolar depression when baseline 25−hydroxyvitamin D [25(OH)D] levels are deficient in both arms. In 25 completers (vitamin D3 group n = 13; placebo group n = 12), Montgomery−Åsberg Depression Rating Scale (MADRS) scores fell substantially in both groups, with week−12 endpoint means of 9.54 versus 6.42 points, respectively. Using endpoint means and standard deviations derived from the reported 95% confidence intervals, the standardized mean difference (Hedges g ≈ −0.36, defined so that positive values would indicate greater improvement with vitamin D3) was transformed via the Chinn method to an odds ratio (OR) ≈ 0.53, with lowerOR ≈ 0.12 and upperOR ≈ 2.21 for clinically meaningful improvement, indicating no statistically significant benefit and a wide confidence interval compatible with both modest harm and modest benefit. Response and remission rates (≥50% MADRS reduction and MADRS ≤ 12) were high and similar in both groups (approximately 77−83%), and secondary outcomes (Young Mania Rating Scale, YMRS; Hamilton Anxiety Rating Scale, HAM−A) also showed no between−group differences. The trial is informative for decision−making as well−tolerated but underpowered negative RCT evidence in bipolar depression.
**27**	Mazahery et al. (2019) [70]	RCT (Randomized, double−blind, placebo−controlled, 2×2 factorial trial)	Included	Included	Provides long−term randomised evidence in children with autism spectrum disorder (ASD), testing vitamin D3 (cholecalciferol), omega−3 long chain polyunsaturated fatty acids (omega−3 LCPUFA), and their combination (VID, OM, VIDOM) versus placebo. The primary

					behavioural outcomes were Social Responsiveness Scale (SRS) and Sensory Processing Measure (SPM). The trial suggests possible benefits of omega−3 LCPUFA, with or without vitamin D_3, on selected SRS and SPM domains but no definitive improvement in SRS−total. The binary "positive response" in SRS−total (≥30% improvement) allows derivation of an odds ratio (OR) for vitamin D−containing arms versus placebo for quantitative synthesis, but precision is very low due to sparse events and high attrition.
**28**	Misal et al. (2024) [71]	RCT (Prospective, randomized controlled, open−label, 4−week augmentation trial)	Included	Excluded	Provides pragmatic augmentation evidence that intramuscular vitamin B_{12} (five 1500 microgram injections over 15 days) added to guideline−based antidepressant treatment in late−life depression (LLD) accelerates and amplifies symptom improvement compared with antidepressant monotherapy. Using remission on the Hamilton Rating Scale for Depression (Hamilton Rating Scale for Depression, HAM−D) at day 28 (HAM−D ≤ 7) as a binary outcome, remission occurred in 13 of 32 participants in the vitamin B_{12} group versus 7 of 30 controls, yielding an odds ratio (OR) ≈ 2.25 with lowerOR ≈ 0.75 and upperOR ≈ 6.76, consistent with a potentially clinically relevant yet imprecisely estimated benefit.
**29**	Mohammadpour et al. (2018) [72]	RCT (Randomized, double−blind, placebo−controlled, parallel−group clinical trial)	Included	Included	Provides child and adolescent ADHD−specific evidence on vitamin D_3 augmentation of psychostimulant treatment. Serum 25−hydroxyvitamin D [25(OH)D] levels were insufficient (<30 nanograms per millilitre, ng/mL) at baseline in all participants and improved substantially only in the vitamin D_3 arm. ADHD symptoms decreased in both groups on Conners' Parent Rating Scale−Revised [S] (CPRS) and ADHD Rating Scale−IV (ADHD−RS), with no between−group differences; however, evening symptoms and total scores on Weekly Parent Ratings of Evening and Morning Behavior (WPREMB) improved significantly more with vitamin D_3. Using WPREMB evening total score at week 8 as a continuous endpoint, Hedges g for lower symptom scores favouring vitamin D3 was approximately 0.69, which corresponds via the Chinn transformation to an odds ratio (OR) ≈ 3.53, with lowerOR ≈ 1.30 and

					upperOR ≈ 9.58 for clinically meaningful reduction in evening ADHD symptoms, albeit with substantial imprecision due to small sample size.
**30**	Naeini et al. (2019) [73]	RCT (Randomized, double−blind, placebo−controlled, parallel−group clinical trial)	Included	Included	Provides child ADHD−specific randomized evidence on low−dose vitamin D3 supplementation in 6−13−year−old students with attention deficit hyperactivity disorder (ADHD) already treated with methylphenidate. Vitamin D_3 (1000 international units, IU) or matching placebo was given once daily for 3 months, with all participants receiving similar doses of methylphenidate. Symptoms were evaluated using Conners Parent Questionnaire (CPQ), Strengths and Difficulties Questionnaire Parent Version (SDQP) and Teacher Version (SDQT), and Continuous Performance Test (CPT) measures (attention, impulsivity, mean reaction time). Using CPQ total score at 3 months as the primary outcome, the standardized mean difference (Hedges g) for lower scores favouring vitamin D_3 was approximately 0.74, which via the Chinn transformation corresponds to an odds ratio (OR) ≈ 3.85 with lowerOR ≈ 1.62 and upperOR ≈ 9.13 for clinically meaningful improvement in parent−rated ADHD symptoms, albeit with notable imprecision due to the modest sample size.
**31**	Okasha et al. (2020) [74]	Observational (Cross−sectional, comparative case−control study)	Included	Included	Provides cross−sectional evidence on the association between vitamin D status and major depressive disorder (MDD) and schizophrenia in an Egyptian sample. Serum 25−hydroxyvitamin D [25(OH)D] was measured using enzyme−linked immunosorbent assay (ELISA), and deficiency severity was categorized as severe deficiency (< 10 nanograms per millilitre, ng/mL), deficiency (10−20 ng/mL), insufficiency (20−30 ng/mL), and sufficiency (30−50 ng/mL). Based on Table 2, vitamin D deficiency defined as < 20 ng/mL was present in 15 of 20 MDD patients, 12 of 20 patients with schizophrenia, and 6 of 20 controls. For the contrast "vitamin D < 20 ng/mL vs ≥ 20 ng/mL", the odds ratio (OR) for major depressive disorder (MDD) versus controls was ≈ 7.00 (lowerOR ≈ 1.74; upperOR ≈ 28.17), and the OR for schizophrenia versus controls was ≈ 3.50 (lowerOR ≈ 0.94; upperOR ≈ 12.97). The authors also reported receiver−operating characteristic (ROC) analyses with an optimal cut−off of ≤ 14 ng/mL for discriminating

					MDD and schizophrenia from controls, although these ROC−based metrics are not directly pooled in the present meta−analysis.
**32**	Okereke et al. (2020) [75]	RCT (Randomized, double−blind, placebo−controlled clinical trial)	Included	Included	Provides high−quality randomized evidence on whether long−term daily vitamin D_3 (cholecalciferol) at 2000 international units (international units, IU) reduces risk of depression or clinically relevant depressive symptoms, defined by incident or recurrent depression events and 8−item Patient Health Questionnaire depression scale (8−item Patient Health Questionnaire depression scale, PHQ−8) scores, over a median 5.3−year follow−up. The primary result for total depression or clinically relevant depressive symptoms was a hazard ratio (hazard ratio, HR) = 0.97 with 95% confidence interval (confidence interval, CI) 0.87−1.09 for vitamin D_3 versus placebo, which can be treated as an odds ratio (odds ratio, OR) ≈ 0.97 with lowerOR ≈ 0.87 and upperOR ≈ 1.09 given the relatively low event rate (~13 per 1000 person−years). No significant differences were observed in longitudinal PHQ−8 mood scores between groups, supporting a null effect of universal vitamin D_3 supplementation on depression prevention in generally vitamin D−replete older adults.
**33**	Omidian et al. (2019) [76]	RCT (Randomized, placebo−controlled, double−blind clinical trial)	Included	Included	Provides randomized evidence on vitamin D_3 monotherapy (without antidepressant drugs) for depressive symptoms in adults with type 2 diabetes mellitus (T2DM) and baseline vitamin D deficiency. Participants (n = 68 randomized; 64 completers) received vitamin D3 4000 international units (IU)/day or placebo for 12 weeks. Beck Depression Inventory−II Persian version (Beck Depression Inventory−II Persian version, BDI−II−PERSIAN) scores decreased from 15.2 ± 9.6 to 9.8 ± 7.2 in the vitamin D group and from 15.5 ± 11.2 to 13.7 ± 11.5 in the placebo group, with a significantly greater percentage reduction in the vitamin D arm (27.6% vs 10.8%; p = 0.02). Using the final Beck Depression Inventory−II (BDI−II) score at 12 weeks as a continuous outcome, the standardized mean difference (Hedges g) for lower scores favouring vitamin D was ≈ 0.40, which via the Chinn transformation corresponds to an odds ratio (OR) ≈ 2.06 with lowerOR ≈ 0.85 and upperOR ≈ 4.99 for clinically meaningful improvement in depressive

					symptoms. Secondary outcomes (hemoglobin A1c, insulin, triglycerides) also improved more in the vitamin D group, suggesting parallel metabolic benefits.
**34**	Penckofer et al. (2022) [77]	RCT (Randomized, double−blind, active comparator−controlled clinical trial)	Included	Excluded	The trial randomized 129 women with type 2 diabetes (T2D), Center for Epidemiologic Studies Depression (Center for Epidemiologic Studies Depression, CES−D) score ≥ 16 (or taking antidepressants with CES−D ≥ 12), and baseline total 25−hydroxyvitamin D [25(OH)D] < 32 nanograms per millilitre (ng/mL) to receive either 50,000 international units (IU) or 5,000 IU of oral vitamin D_3 weekly for six months. Depressive symptoms were assessed with the CES−D and Patient Health Questionnaire−9 (Patient Health Questionnaire−9, PHQ−9) at baseline, 3 and 6 months. Serum 25(OH)D increased by about +34 ng/mL in the high−dose group and +10 ng/mL in the low−dose group, but there was no statistically significant difference between dosing groups in mean CES−D or PHQ−9 change over time. Depression remission (CES−D < 16) at six months occurred in 53% (33/62) of women on 50,000 IU and 60% (34/57) on 5,000 IU, yielding an odds ratio (odds ratio, OR) of approximately 0.77 (lowerOR ≈ 0.37; upperOR ≈ 1.59) for remission with high−dose versus low−dose vitamin D3. Both regimens were generally well tolerated, with a small number of hypervitaminosis D and hypercalcaemia events managed by discontinuation of the study drug. These results support the safety and potential mood benefits of vitamin D_3 supplementation in this high−risk metabolic−psychiatric population but do not demonstrate superiority of a very high weekly dose over a physiological replacement dose.
**35**	Petruzzelli et al. (2020) [78]	Observational (Retrospective cross−sectional case−control study)	Included	Included	The study enrolled 54 inpatients with autism spectrum disorder (ASD group) and 36 inpatients with other neurological or psychiatric diagnoses (non−ASD group) admitted to the Child Neuropsychiatry Unit, University of Bari “Aldo Moro”, Italy (2014−2018). Serum 25−hydroxyvitamin D [25(OH)D] was measured by chemiluminescence immunoassay and categorized as deficiency (< 20 ng/mL), insufficiency (20−30 ng/mL) or normality (30−100 ng/mL). Mean 25(OH)D was significantly lower in the autism spectrum disorder (ASD) group (18.61

					± 8.33 ng/mL) than in the non−ASD group (24.62 ± 13.18 ng/mL; p = 0.014). A multivariable logistic regression model adjusting for age and sex showed that vitamin D deficiency (< 20 ng/mL) was strongly associated with autism spectrum disorder (ASD), with an adjusted odds ratio (odds ratio, OR) = 10.31 and 95% confidence interval (confidence interval, CI) 1.96−54.22 for deficiency versus normality, while insufficiency (20−30 ng/mL) showed a non−significant intermediate association (OR = 3.29; 95% CI 0.61−17.71). These data provide an extractable odds ratio (OR) for the association between vitamin D deficiency and autism spectrum disorder (ASD) suitable for inclusion in the meta−analysis.
**36**	Rahman et al. (2023) [79]	RCT (Randomized, double−blind, placebo−controlled trial)	Included	Included	Large population−based randomized controlled trial (RCT) of monthly high−dose vitamin D_3 supplementation (60,000 international units, IU) versus placebo for up to five years in 21,315 community−dwelling older Australian adults (aged 60−84 years). Depressive symptoms were assessed repeatedly using the Patient Health Questionnaire−9 (PHQ−9), and incident antidepressant use was ascertained via national Pharmaceutical Benefits Scheme (PBS) records. The primary depression−related effect retained for harmonization is the odds ratio (OR) for clinically relevant depression defined as PHQ−9 score ≥10 (vitamin D_3 versus placebo), overall OR = 0.99 with lowerOR = 0.90 and upperOR = 1.08, indicating no material effect of supplementation on depression risk in this largely vitamin D−replete population. The trial provides high−quality evidence to inform decision−making about the limited utility of routine high−dose vitamin D supplementation for prevention of late−life depression, while suggesting possible benefit only in subgroups with low predicted serum 25−hydroxyvitamin D [25(OH)D] concentration or concurrent antidepressant treatment at baseline.
**37**	Rouhi et al. (2018) [80]	RCT (Randomised, double−blind, placebo−controlled clinical trial)	Included	Included	Eighty primiparous Iranian women with elevated depressive symptoms (Edinburgh Postnatal Depression Scale, EPDS ≥ 13) and fatigue (Fatigue Identification Form, FIF ≥ 20) were randomized 1:1 to vitamin D_3 1,000 international units (IU) daily or placebo for six months. Depression and

					fatigue scores were measured at baseline (≈ 4 months postpartum) and after 6 months (≈ 10 months postpartum) using validated self−report scales. At 10 months postpartum, mean Edinburgh Postnatal Depression Scale (EPDS) scores were 8.6 ± 4.3 in the vitamin D group versus 13.4 ± 4.7 in the placebo group, with similar baseline scores, yielding a standardized mean difference (Hedges g) ≈ 1.06 in favour of vitamin D. Using the Chinn transformation, this corresponds to an odds ratio (OR) ≈ 6.78, lowerOR ≈ 2.90, upperOR ≈ 15.86 for clinically meaningful improvement in depressive symptoms with vitamin D_3 versus placebo. Fatigue scores showed an even larger benefit, but are not included in the meta−analysis. These results support a substantial antidepressant effect of low−dose daily vitamin D_3 in high−risk postpartum women, although the small sample size and lack of biochemical vitamin D measurements limit certainty.
**38**	Shahini et al. (2022) [81]	Observational (Case−control observational study)	Included	Included	Thirty−three adult patients with schizophrenia diagnosed by semi−structured interviews based on Diagnostic and Statistical Manual of Mental Disorders, Fifth Edition (Diagnostic and Statistical Manual of Mental Disorders, Fifth Edition, DSM−5) criteria and treated with atypical antipsychotics were compared with 33 age− and sex−matched psychiatrically healthy controls from the same region. Serum vitamin D and homocysteine concentrations were measured using commercial kits, and extrapyramidal symptoms and psychopathology were assessed with the Simpson−Angus Extrapyramidal Side Effects Scale (Simpson−Angus Extrapyramidal Side Effects Scale, SAS) and the Positive and Negative Syndrome Scale for Schizophrenia (Positive and Negative Syndrome Scale for Schizophrenia, PANSS). Vitamin D levels were significantly lower and homocysteine levels significantly higher in schizophrenic patients than in controls. When vitamin D was dichotomized as severely deficient (< 10 μg/dL) versus non−deficient (≥ 10 μg/dL), the 2×2 table (deficient vs non−deficient by schizophrenia vs control status) yielded an odds ratio (OR) ≈ 2.30 with lowerOR ≈ 0.80 and upperOR ≈ 6.61 for schizophrenia in the presence of severe vitamin D deficiency. The study also reported an inverse correlation between

					vitamin D and homocysteine levels and a positive correlation between vitamin D levels and severity of extrapyramidal symptoms on the Simpson−Angus Extrapyramidal Side Effects Scale (SAS), but not with Positive and Negative Syndrome Scale for Schizophrenia (PANSS) factors.
**39**	Sourander et al. (2021) [82]	Observational (Nationwide nested case−control study)	Included	Included	A total of 1,558 ASD cases born between 1987 and 2004 were identified from the Finnish Care Register for Health Care and individually matched 1:1 to 1,558 controls without autism spectrum disorder (autism spectrum disorder, ASD) or intellectual disability (intellectual disability, ID) on sex and date of birth (± 30 days). Maternal serum 25−hydroxyvitamin D [25(OH)D] was measured from first− and early second−trimester samples stored in the Finnish Maternity Cohort (Finnish Maternity Cohort, FMC) biobank using a chemiluminescence microparticle immunoassay, and values were analysed as continuous, quintile−based, and clinical categories (deficient < 30 nmol/L, insufficient 30−49.9 nmol/L, sufficient ≥ 50 nmol/L). Conditional logistic regression, adjusting for maternal age, gestational week of blood draw, gestational age, season of blood collection, smoking, immigration status, psychopathology, and substance abuse, showed that deficient (< 30 nmol/L) and insufficient (30−49.9 nmol/L) maternal 25−hydroxyvitamin D [25(OH)D] levels were associated with increased odds of autism spectrum disorder (autism spectrum disorder, ASD) in offspring compared with sufficient levels (≥ 50 nmol/L). For the key contrast used in this meta−analysis (deficient vs sufficient), the fully adjusted odds ratio (OR) was ≈ 1.52 with lowerOR ≈ 1.20 and upperOR ≈ 1.91. The association was strongest for pervasive developmental disorder/pervasive developmental disorder−not otherwise specified (pervasive developmental disorder/pervasive developmental disorder−not otherwise specified, PDD/PDD−NOS) and was observed mainly in autism spectrum disorder (autism spectrum disorder, ASD) without intellectual disability (intellectual disability, ID), supporting prenatal vitamin D deficiency as a modifiable risk factor for autism spectrum disorder (autism spectrum disorder, ASD).

**40**	Terock et al. (2020) [83]	Observational (Population−based cross−sectional analysis)	Included	Included	In a sample of N = 1,653 adults with at least one traumatic event from the Study of Health in Pomerania (Study of Health in Pomerania, SHIP−1), the authors examined serum 25−hydroxyvitamin D [25(OH)D] concentrations and vitamin D deficiency status in relation to posttraumatic stress disorder (posttraumatic stress disorder, PTSD), assessed with the PTSD module of the Structured Clinical Interview for the Diagnostic and Statistical Manual of Mental Disorders, Fourth Edition (Diagnostic and Statistical Manual of Mental Disorders, Fourth Edition, DSM−IV) (current PTSD in 63 participants; 3,8%). Multivariable logistic regression adjusted for sex, age, waist circumference, physical inactivity, years of schooling, depressive symptoms, and season of blood sampling showed that higher continuous 25−hydroxyvitamin D [25(OH)D] levels were associated with lower odds of posttraumatic stress disorder (posttraumatic stress disorder, PTSD) (odds ratio, OR ≈ 0,96 per 1 ng/mL; 95% confidence interval, CI ≈ 0,93−0,99), while vitamin D deficiency defined as 25−hydroxyvitamin D [25(OH)D] < 20 ng/mL was associated with higher odds of posttraumatic stress disorder (posttraumatic stress disorder, PTSD) (OR = 2,02; lowerOR = 1,10; upperOR = 3,89). Additional analyses linked functional polymorphisms in the vitamin D−binding protein (vitamin D−binding protein, VDBP; rs4588 and rs7041) and combined Gc genotypes (Gc1s, Gc1f, Gc2) to both 25−hydroxyvitamin D [25(OH)D] concentrations and posttraumatic stress disorder (posttraumatic stress disorder, PTSD), suggesting that altered vitamin D metabolism and vitamin D−binding protein genetic variation may contribute to trauma−related psychopathology and to comorbid cardiometabolic risk.
**41**	Van der Leeuw et al. (2020) [84]	Observational (Cross−sectional observational study)	Included	Included	The study analyzed N = 629 participants (347 patients with psychotic disorder and 282 controls) recruited from the Netherlands and nearby regions, with diagnoses based on the Comprehensive Assessment of Symptoms and History (Comprehensive Assessment of Symptoms and History, CASH) according to the Diagnostic and Statistical Manual of Mental Disorders, Fourth Edition (Diagnostic and Statistical Manual of Mental Disorders, Fourth Edition, DSM−IV). Serum 25−hydroxyvitamin

					D [25(OH)D] was measured using online solid−phase extraction followed by liquid chromatography−tandem mass spectrometry, and vitamin D status was classified as sufficient above 75 nanomoles per litre (nmol/L). Patients had significantly lower mean 25−hydroxyvitamin D [25(OH)D] than controls (47,0 ± 26,1 vs 60,1 ± 26,5 nmol/L), and in adjusted linear regression the patient group coefficient was B = −8,05 (95% confidence interval, CI −13,68 to −2,42). Using the reported 2×2 distribution of sufficient vitamin D (≥ 75 nmol/L) in controls (77/282; 37,5%) and patients (51/347; 17,2%), we derived a crude odds ratio (OR) for psychotic disorder in individuals with insufficient vitamin D (< 75 nmol/L) versus sufficient levels: OR ≈ 2,18, lowerOR ≈ 1,47, upperOR ≈ 3,24. Higher 25−hydroxyvitamin D [25(OH)D] levels were modestly associated with lower Positive and Negative Syndrome Scale (Positive and Negative Syndrome Scale, PANSS) positive and negative symptom scores, and birth urbanicity showed a negative association with vitamin D concentrations in patients but not in controls, suggesting an interplay between early−life urban environmental adversity, vitamin D status and psychosis risk.
**42**	Vaziri et al. (2016) [85]	RCT (Randomized, placebo−controlled, single−blind clinical trial)	Included	Included	This trial directly tests whether correcting low serum 25−hydroxyvitamin D [25(OH)D] during late pregnancy reduces ante− and postnatal depressive symptoms, providing randomized evidence on vitamin D3 as a preventive/adjunctive strategy for perinatal depression that is highly relevant for our question on modifiable nutritional biomarkers and mood disorders.
**43**	Vellekkatt et al. (2020) [86]	RCT (Randomized, double−blind, parallel−arm, placebo−controlled trial)	Included	Included	The trial directly tests whether correcting assay−confirmed serum 25−hydroxyvitamin D [25(OH)D] deficiency via high−dose parenteral vitamin D_3 improves depressive symptoms, quality of life and illness severity when used as an adjunct to antidepressant pharmacotherapy in major depressive disorder (major depressive disorder, MDD), providing mechanistically relevant evidence for our question on vitamin D as a modifiable nutritional biomarker in mood disorders.
**44**	Yazici et al. (2019) [87]	Observational (Retrospective cross−sectional study)	Included	Included	Three−group observational dataset with clearly defined Diagnostic and Statistical Manual of Mental Disorders, Fifth Edition (DSM−5)

					schizophrenia and substance use disorder diagnoses and a hospital–based healthy control group; reports group–specific prevalence of vitamin D, vitamin B_{12} and folate deficiency based on standardized cut–offs, allowing reconstruction of 2×2 tables and calculation of odds ratios for low vitamin status in psychiatric versus control groups; included in the meta–analysis as a source of odds ratios for vitamin B_{12} deficiency (primary quantitative effect) and low vitamin D in sensitivity analyses, while acknowledging cross–sectional design and residual confounding.
**45**	Yee et al. (2016) [88]	Observational (Cross–sectional case–control study)	Included	Included	Provides clinically well–characterised first–episode psychosis (first–episode psychosis, FEP) cases and matched controls from a tropical setting with year–round sunlight, with standardized chemiluminescence assays for total vitamin D and vitamin D–binding protein (vitamin D–binding protein, DBP) and derived bioavailable vitamin D; includes group means and standard deviations allowing standardized mean differences for bioavailable vitamin D to be converted to odds ratios for psychiatric versus control status, directly informing our question on vitamin D status in early psychosis.
**46**	Yektaş et al. (2019) [89]	Observational (Single–centre, cross–sectional case–control study)	Included	Included	Provides three–group observational data with clearly defined attention deficit hyperactivity disorder (attention deficit hyperactivity disorder, ADHD) and autism spectrum disorder (autism spectrum disorder, ASD) diagnoses and a medication–free healthy control group; reports group medians and inter–quartile ranges for vitamin B_{12}, folate and homocysteine, allowing approximate derivation of standardized mean differences for autism spectrum disorder versus controls and attention deficit hyperactivity disorder versus controls and conversion to odds ratios (ORs) via the Chinn method for homocysteine as the primary quantitative outcome; directly informs paediatric autism spectrum disorder and attention deficit hyperactivity disorder subgroups in our meta–analysis of vitamin B_{12} and homocysteine–related markers.

Table S3. Studies excluded from the qualitative and/or quantitative synthesis: rationale aligned with prespecified eligibility criteria.

Record ID	Study	Design	Eligibility Outcome	Basis for Decision (Criterion & Rationale)
**1**	Abiri & Vafa (2020), Iran [90]	Study protocol for a double−blind, randomized, placebo−controlled factorial (RCT)	Excluded	Protocol only; no clinical results available → no extractable effect estimates (SMD/OR/MD) for mood or biomarkers; therefore does not meet inclusion criterion requiring primary outcome data.
**2**	Albuloshi et al. (2022), UK [91]	Systematic review and meta−analysis of randomized controlled trials (RCTs)	Excluded	Secondary research article that synthesizes randomized controlled trials rather than reporting new primary trial data; does not provide individual trial−level data beyond what is already available in the original randomized controlled trials and overlaps with several randomized controlled trials included as primary evidence in our review; including this systematic review and meta−analysis in the quantitative synthesis would lead to double−counting of the same participants and violate our protocol, which restricts eligibility for meta−analysis to primary randomized controlled trials and observational studies with extractable effect sizes; therefore the article is excluded from the primary SR/MA.
**3**	Casseb et al. (2019), Brazil [92]	Narrative review (leading article) summarising preclinical and clinical evidence.	Excluded	Secondary research article (narrative review) that synthesises animal and human evidence without reporting new primary patient−level data or extractable effect sizes; overlaps substantially with primary randomized controlled trials and observational studies already included in our dataset; inclusion in the SR/MA pool would lead to double−counting of participants and violate our predefined eligibility criteria restricting quantitative synthesis to primary studies with extractable odds ratios (ORs) or convertible standardized mean differences; therefore, retained only as a background mechanistic and contextual source, not as a primary data contributor.
**4**	Centeno et al. (2024), Brazil [93]	Systematic review with meta−analysis synthesising cohort, cross−sectional and case−control studies.	Excluded	Secondary research article (systematic review and meta−analysis) without new primary patient−level data; all quantitative results are derived from primary cohort, cross−sectional and case−control studies, several of which are already included as primary evidence in our dataset. Including this paper as

				if it were a primary study would double−count participants and violate our predefined eligibility criteria that restrict the quantitative synthesis to original randomized controlled trials (randomized controlled trials, RCTs) and observational studies reporting extractable effect estimates (odds ratios, ORs, or standardized mean differences, SMDs). Therefore, Centeno et al. (2024) is excluded from the primary SR/MA pool.
**5**	Cheng et al. (2020), Taiwan [94]	Systematic review and meta−analysis of randomized controlled trials (RCTs)	Excluded	Secondary research article (systematic review and meta−analysis) that re−analyses existing randomized controlled trials without providing new primary patient−level data; its quantitative results are entirely based on original RCTs, many of which are already included as primary studies in our own dataset. Including Cheng et al. (2020) as if it were a primary study in our meta−analysis would lead to double−counting of participants and violate our predefined eligibility criteria, which restrict the quantitative synthesis to individual randomized controlled trials and observational studies with extractable effect estimates (odds ratios, ORs, or convertible standardized mean differences, SMDs). Therefore, the study is excluded from the primary SR/MA pool.
**6**	Cui et al. (2021), Australia [95]	Expert review combined with systematic review and meta−analysis.	Excluded	Secondary research article (expert narrative review plus systematic review and meta−analysis) that re−analyses existing primary randomized controlled trials (randomized controlled trials, RCTs) and observational studies without presenting new, independent patient−level data; many of the trials and observational cohorts included in Cui et al. are already represented individually in our dataset. Including this paper as if it were a primary study would lead to double−counting participants and violate our predefined eligibility criteria, which restrict the quantitative synthesis to original RCTs and observational studies reporting extractable effect estimates (odds ratios, ORs, or convertible standardized mean differences, SMDs). Therefore, Cui et al. (2021) is excluded from the primary SR/MA pool.
**7**	Gaughran et al. (2020), UK [96]	Protocol for a randomized, double−blind, placebo−controlled parallel−group randomized controlled trial (RCT).	Excluded	Protocol−only article following Standard Protocol Items: Recommendations for Interventional Trials (SPIRIT) guidance, reporting design, eligibility criteria, dosing schedule and planned statistical analyses but not publishing any primary clinical outcome data or effect estimates (e.g., odds ratios [ORs]) for symptom scales, functioning or cardiometabolic biomarkers; therefore, it

				does not meet the prespecified inclusion criterion requiring extractable effect sizes from completed primary studies and cannot contribute to the qualitative or quantitative evidence synthesis.
**8**	Ghaemi et al. (2024), Iran [97]	Systematic review and dose–response meta–analysis of randomized controlled trials (RCTs).	Excluded	Secondary research article (systematic review and dose–response meta–analysis) that re–analyses existing randomized controlled trials without providing new, independent patient–level data; most of the contributing randomized controlled trials (randomized controlled trials, RCTs) are already included individually in our dataset as primary evidence. Including this paper as if it were a primary trial would double–count participants and violate our predefined eligibility criteria, which restrict the quantitative synthesis to original randomized controlled trials and observational studies with extractable odds ratios (odds ratios, ORs, or convertible standardized mean differences, SMDs). Furthermore, the study reports only pooled standardized mean differences on the SMD scale, whereas our meta–analysis standardises all primary results to the odds ratio scale; incorporating this secondary pooled effect would mix levels of evidence and distort weighting. Therefore, Ghaemi et al. (2024) is excluded from the primary SR/MA dataset.
**9**	Głąbska et al. (2021), Poland [98]	Systematic review of intervention and observational studies.	Excluded	Secondary research article (systematic review without de novo individual–level data) that collates 24 primary studies and presents a narrative synthesis of mental health outcomes in children and adolescents; it does not provide new, independent effect estimates beyond those already reported in the underlying randomized controlled trials and observational studies. Several of those primary studies overlap conceptually with our target domain, but the current project is restricted by protocol to primary randomized controlled trials and observational studies with extractable odds ratios (odds ratios, ORs) or convertible standardized mean differences, and to psychiatric diagnoses or symptom constructs directly linked to serotonergic and dopaminergic dysregulation (for example, autism spectrum disorder, attention deficit hyperactivity disorder, major depressive disorder, psychosis). Including this secondary synthesis as if it were a primary study would double–count participants and mix evidence levels, potentially

				biasing our meta−analytic weighting. Therefore, the study is excluded from the primary SR/MA dataset.
**10**	Khoshbakht et al. (2018), Iran [99]	Systematic review and meta−analysis of observational studies (case−control, cross−sectional, and cohort designs).	Excluded	Secondary research article that aggregates 13 observational studies without providing new individual−level data; all quantitative estimates (mean differences, odds ratios, risk ratios) are derived from primary studies, some of which overlap conceptually with the populations and outcomes targeted in our review. Including this systematic review and meta−analysis as if it were a primary study would double−count participants and violate our predefined eligibility criteria, which restrict the quantitative synthesis to original randomized controlled trials and observational studies with directly extractable effect sizes on the odds ratio (odds ratio, OR) scale or convertible standardized mean differences; therefore, the article is excluded from the primary SR/MA dataset.
**11**	Kumar et al. (2022), India [100]	Randomized, double−blind, placebo−controlled 12−week randomized controlled trial (RCT).	Excluded	Abstract−only report; the available PDF contains only a structured abstract without full tables or dispersion measures (for example, standard deviations, confidence intervals or exact test statistics) for Hamilton Depression Rating Scale (HDRS) change or responder outcomes. As a result, no robust effect size (odds ratio, OR, with 95% confidence interval, CI) can be directly computed from the primary outcome, and any estimate would rely on indirect approximation from the p−value. This does not meet the prespecified inclusion criterion requiring extractable primary outcome data from full−text reports; therefore, the study is excluded from both qualitative and quantitative synthesis.
**12**	Lally et al. (2016), UK [101]	Cross−sectional baseline analysis of community−dwelling adults.	Excluded	Primary analysable outcomes are cardiometabolic (metabolic syndrome and related risk markers) rather than psychiatric incidence or symptom severity as defined in the protocol. Serum 25−hydroxyvitamin D [25(OH)D] levels were strongly associated with metabolic syndrome (for example, lowest vs highest quartile odds ratio (OR) ≈ 2.48), but there were no odds ratios or risk estimates reported for binary psychiatric outcomes (for example, presence/absence of depression, severe vs non−severe psychotic symptoms), and only non−significant correlations with continuous symptom scales (Positive and Negative Syndrome Scale (PANSS), Montgomery−Åsberg Depression Rating Scale (MADRS)). Deriving ad hoc cut−offs on these scales

				to construct psychiatric ORs would conflict with the prespecified requirement for directly extractable, author−defined psychiatric outcomes. Therefore, the study does not meet the inclusion criterion for quantitative or qualitative synthesis focused on psychiatric disorder risk or symptom severity and is classified as excluded.
**13**	Lally et al. (2019), Ireland [102]	Prospective cohort study (observational) in adults.	Excluded	Mental state outcomes (PANSS total and subscales, depressive symptoms, functioning) are modelled using linear regression with 25(OH)D as a continuous predictor and as quartiles, reported only as beta (β) coefficients with 95% confidence intervals (CI), without group−level means/standard deviations or dichotomous clinical outcomes that could be transformed into standardized mean differences (SMD) or odds ratios (OR) under the pre−specified conversion rules. Because no extractable binary or directly convertible continuous effect size compatible with the review's harmonized OR metric is available, the study does not meet the quantitative data requirements for inclusion in the meta−analysis.
**14**	Li et al. (2022), China [103]	Systematic review and meta−analysis of randomized controlled trials (RCTs).	Excluded	Secondary research article that re−analyses existing randomized controlled trials without providing new, independent patient−level data; the RCTs contributing to this meta−analysis (for example, Mazahery et al., Kerley et al., Azzam et al., Moradi et al.) are already considered individually as primary evidence in our dataset. Including Li et al. (2022) as if it were a primary study would double−count participants and violate our predefined eligibility criteria, which restrict the quantitative synthesis to original randomized controlled trials and observational studies reporting extractable effect sizes on the odds ratio (odds ratio, OR) scale or convertible standardized mean differences, SMDs. Therefore, the article is excluded from the primary SR/MA dataset.
**15**	Markun et al. (2021), Switzerland [104]	Systematic review, meta−analysis, and meta−regression of randomized controlled trials (RCTs).	Excluded	Secondary research article synthesising previously published randomized controlled trials without providing new, independent patient−level data; several of the underlying RCTs overlap with trials considered individually in our dataset. Our protocol restricts inclusion in the primary SR/MA dataset to original randomized controlled trials and observational studies with extractable effect estimates that can be harmonised on the odds ratio (odds ratio, OR) scale, whereas Markun et al. report only pooled standardized mean

				differences, SMDs, and meta−regression coefficients. Including this meta−analysis as if it were a primary study would double−count participants and conflate evidence levels. Therefore, the study is excluded from the quantitative dataset.
**16**	Mikola et al. (2023), Finland [105]	Systematic review and meta−analysis of randomized controlled trials (RCTs)	Excluded	Secondary research article that aggregates 41 randomized controlled trials without providing new individual−level outcome data; most of the contributing randomized controlled trials (randomized controlled trials, RCTs) overlap directly with the primary evidence already included in our dataset. Our protocol restricts the quantitative synthesis to original randomized controlled trials and observational studies with extractable effect sizes that can be harmonised on the odds ratio (odds ratio, OR) scale (or converted from study−level standardized mean differences, SMDs) and explicitly excludes higher−level meta−analyses to avoid double counting and mixing evidence levels. Mikola et al. report only pooled standardized mean differences and meta−analytic subgroup estimates, not trial−specific raw data beyond what is available in the original publications. Including this meta−analysis as if it were a primary study would therefore double−count participants and distort weighting in our own meta−analysis. Consequently, the study is excluded from the primary SR/MA dataset.
**17**	Mikola et al. (2024), Finland [106]	Study protocol and baseline characteristics for a double−blind, randomized, parallel−group.	Excluded	Protocol−only article presenting trial rationale, methods, and baseline characteristics; no post−randomisation outcome data or between−group comparisons in MADRS, BDI, or other psychiatric endpoints are reported. Consequently, there are no extractable effect estimates (odds ratio [OR], risk ratio, mean difference, or standardized mean difference) for depressive symptoms or related psychiatric outcomes. This violates the pre−specified inclusion criterion requiring primary outcome data suitable for quantitative synthesis; therefore the study does not contribute to either the qualitative evidence synthesis or the meta−analysis and is classified as excluded.
**18**	Musazadeh et al. (2023), Iran [107]	Umbrella meta−analysis of interventional and observational meta−analyses.	Excluded	Tertiary−level evidence synthesis that aggregates previously published meta−analyses of randomized controlled trials and observational studies without providing new primary patient−level or trial−level data; all quantitative effect estimates are derived from underlying meta−analyses that, in turn, are based on original randomized controlled trials and cohorts

				already considered individually in our dataset. Our protocol restricts inclusion in the primary systematic review and meta−analysis dataset to original randomized controlled trials and observational studies with extractable effect sizes that can be harmonised on the odds ratio (odds ratio, OR) scale (or converted from study−level standardized mean differences, SMDs), and explicitly excludes higher−order umbrella meta−analyses to avoid double− or triple−counting participants and mixing evidence levels. Consequently, Musazadeh et al. (2023) is excluded from the quantitative dataset.
**19**	Öztürk et al. (2020), Turkey [108]	Cross−sectional, retrospective chart−review study.	Excluded	The study lacks a non−psychiatric control group and reports only within−cohort comparisons (ADHD versus ADHD−NOS) and correlations between continuous vitamin B_{12} and folate levels and symptom scales. No 2 × 2 data or group contrasts compatible with our predefined psychiatric versus non−psychiatric outcomes are available, and no standardized effect estimates (odds ratio, OR) can be derived for inclusion in the meta−analysis. Moreover, vitamin B_{12} and folate were measured only in subsets of the cohort (n = 89 and n = 82 of 205, respectively), with no multivariable adjustment for key confounders. In line with the updated protocol decision to retain only studies contributing to both the systematic review and meta−analysis, this study is therefore classified as excluded.
**20**	Razavinia et al. (2024), Iran [109]	Systematic review and meta−analysis of case−control and cross−sectional observational studies.	Excluded	Secondary research article that aggregates six primary observational studies without providing new, independent patient−level data; all quantitative estimates (standardized mean differences, SMDs) for vitamin B_9 and vitamin B_{12} are derived from underlying case−control and cross−sectional studies. Our protocol restricts the quantitative synthesis to original randomized controlled trials (randomized controlled trials, RCTs) and observational cohorts or case−control studies with directly extractable effect sizes that can be harmonised on the odds ratio (odds ratio, OR) scale (or converted from study−level standardized mean differences, SMDs). Including this systematic review and meta−analysis as if it were a primary study would double−count participants and mix evidence levels. Therefore, Razavinia et al. (2024) is excluded from the primary SR/MA dataset.

**21**	Tan et al. (2023), China [110]	Systematic review and meta–analysis of observational studies (cohort, case–control, and cross–sectional designs).	Excluded	Secondary research article that aggregates observational studies without providing new, independent individual–level data; all quantitative estimates (standardized mean differences, SMDs, and odds ratios, ORs) are derived from primary cohorts and case–control studies, several of which overlap directly with the original studies considered as eligible in our dataset. Our protocol restricts inclusion in the primary systematic review and meta–analysis dataset to original randomized controlled trials (randomized controlled trials, RCTs) and observational studies with directly extractable effect sizes that can be harmonised on the odds ratio (odds ratio, OR) scale (or converted from study–level standardized mean differences, SMDs). Including Tan et al. (2023) as if it were a primary study would double–count participants, mix evidence levels, and over–weight already represented cohorts. Therefore, this article is excluded from the quantitative dataset.
**22**	Tirani et al. (2023), Iran [111]	Systematic review and dose–response meta–analysis of observational studies.	Excluded	Secondary research article that aggregates ten primary cohort and case–control studies without providing new individual–level data; all quantitative estimates (odds ratios, risk ratios, and dose–response slopes) are derived from underlying observational studies. Our protocol restricts inclusion in the primary systematic review and meta–analysis dataset to original randomized controlled trials and observational studies with directly extractable effect sizes that can be harmonised on the odds ratio (odds ratio, OR) scale. Including this meta–analysis as if it were a primary study would double–count participants and mix evidence levels. Therefore, Tirani et al. (2023) is excluded from the quantitative dataset.
**23**	Upadhyaya et al. (2023), Finland [112]	Systematic review of longitudinal observational studies (cohort, nested case–control, and case–control designs).	Excluded	Secondary research article that aggregates 29 primary longitudinal observational studies without providing new, independent patient–level or study–level effect estimates beyond those reported in the original publications; results are presented mainly as narrative syntheses and direction–of–effect summaries in line with Synthesis Without Meta–analysis, SWiM guidance, rather than as harmonised odds ratios (odds ratios, ORs) that could be directly integrated into our quantitative dataset. Our protocol restricts inclusion in the primary systematic review and meta–analysis dataset to original randomized controlled trials and observational studies with extractable effect sizes that can be standardised on the odds ratio (odds

				ratio, OR) scale, and explicitly excludes higher−level systematic reviews to avoid double−counting participants and conflating evidence tiers. Several of the cohorts covered by Upadhyaya et al. overlap with primary studies already included or considered in our own screening set. Therefore, this article is excluded from the quantitative dataset.
**24**	Wang et al. (2021), China [113]	Systematic review and meta−analysis of observational studies (case−control, cohort, and nested case−control designs).	Excluded	Secondary research article that synthesises 34 publications (36 study samples) without providing new, independent individual−level data; all quantitative estimates (mean differences, odds ratios, and relative risks) are derived from primary case−control and prospective studies, several of which overlap directly with the original studies considered for inclusion in our dataset. Our protocol restricts the quantitative synthesis to original randomized controlled trials and observational studies with extractable effect sizes that can be harmonised on the odds ratio (odds ratio, OR) scale (or converted from study−level standardized mean differences), and explicitly excludes higher−level systematic reviews and meta−analyses to avoid double−counting participants and conflating evidence tiers. Consequently, Wang et al. is excluded from the primary SR/MA dataset.
**25**	Wilczyński et al. (2022), Poland [114]	Systematic review and meta−analysis of observational studies (cross−sectional, case−control, and cohort designs).	Excluded	Secondary research article aggregating 18 primary observational studies without providing new, independent individual−level data; all odds ratios and confidence intervals are derived from underlying studies that themselves vary widely in design, population, and covariate adjustment. Our protocol restricts inclusion in the primary systematic review and meta−analysis dataset to original randomized controlled trials and observational studies published between 2016 and 2025 with directly extractable effect sizes that can be harmonised on the odds ratio scale; higher−level meta−analyses are reserved for background to avoid double−counting participants and conflating evidence tiers. Therefore, Wilczyński et al. (2022) is excluded from the quantitative dataset.
**26**	Young et al. (2019), Australia [115]	Systematic review and meta−analysis of randomized controlled trials (RCTs).	Excluded	Secondary research article that aggregates 16 randomized controlled trials (18 articles) without providing new, independent patient−level data; all effect estimates (standardized mean differences, SMDs) for mood outcomes are derived from underlying RCTs, several of which overlap with primary trials considered in our own dataset. Our protocol restricts the quantitative

				synthesis to original randomized controlled trials and observational studies with directly extractable effect sizes that can be harmonised on the odds ratio (odds ratio, OR) scale (or converted from study−level standardized mean differences, SMDs), and explicitly excludes higher−level systematic reviews and meta−analyses to avoid double−counting participants and conflating evidence tiers. Consequently, Young et al. (2019) is excluded from the primary SR/MA dataset.
**27**	Zhang et al. (2023), China [116]	Systematic review and meta−analysis of randomized controlled trials (RCTs).	Excluded	Secondary research article that aggregates eight randomized controlled trials (six included in the meta−analysis) without providing new, independent patient−level data; all quantitative estimates (mean differences for symptom scales and 25−hydroxyvitamin D [25(OH)D]) are derived from underlying randomized controlled trials that are considered individually as primary evidence in our dataset. Our protocol restricts inclusion in the primary systematic review and meta−analysis dataset to original randomized controlled trials and observational studies with extractable effect sizes that can be harmonised on the odds ratio (odds ratio, OR) scale (or converted from study−level standardized mean differences, SMDs) and explicitly excludes higher−level systematic reviews and meta−analyses to avoid double−counting participants and conflating evidence tiers. Therefore, Zhang et al. (2023) is excluded from the quantitative dataset.

Table S4. Overview of included studies and consolidated key characteristics.

ID	Authors (year, country)*	Study design	Population (N; Sex: male/female, n (%); Age: mean (SD))	Sample size		Outcomes (effect estimate; 95% CI)	Biomarker/ Clinical diagnosis/ Clinical_subgroup	Interpretation of Findings
				Exposed n (%)	Comparator n (%)			
**1**	Ali et al. (2019), Canada [44]	Observational (Prospective cohort study)	N=3,852; Male=2,003 (52.0%); Female=1,849 (48.0%); Age=2.5 (1.6) years; Children/Adolescents	1,989 (52.0%)	1,836 (48.0%)	Adjusted RR = 1.06 (95% CI: 0.95−1.18) per 10 nmol/L increase in baseline serum 25(OH)D (incident physician−diagnosed ASD)	Vitamin D (Status vs Outcome). Autism spectrum disorder (ASD). Mixed/Others.	Baseline serum 25(OH)D status was not associated with incident ASD in the adjusted analysis (effect estimate close to the null and 95% CI including 1.0), providing no evidence of a protective or harmful relationship between higher early−childhood 25(OH)D levels and subsequent physician−diagnosed ASD in this cohort.
**2**	Allott et al. (2019), Australia [45]	RCT (Randomized double−blind placebo−controlled trial)	N=100; Male=68 (68.0%); Female=32 (32.0%); Age=19.9 (2.7) years; Children/Adolescents	52 (52.0%)	48 (48.0%)	Primary (coprimary) clinical outcome (PANSS Total; Week 12 − baseline): Vitamins: −4.76 (SD 13.98), n = 47; Placebo: −5.41 (SD 14.13), n = 48. MD (Vitamins − Placebo) = 0.65 (95% CI: −5.00 to 6.30)	Vitamin B_{12} (Supplementation vs Placebo). First−episode psychosis (schizophrenia spectrum and affective psychoses). Dopaminergic.	Adjunctive B−vitamin supplementation (folic acid + vitamin B_{12} + vitamin B_6) did not demonstrate a statistically significant advantage over placebo on the coprimary clinical outcome (PANSS total symptom change) over 12 weeks, despite biochemical evidence of homocysteine reduction.

						(computed from group change scores; as reported: p = .749; effect size = 0.07).		
**3**	Altun et al. (2018), Turkey [46]	Observational (Case−control study)	N =105; Male=88 (83.8%); Female= 17 (16.2%); Age= 6.2 (2.6). Children/Adolescents	60 (57.1%)	45 (42.9%)	Vitamin B_{12} status (pg/mL), ASD (n = 60) vs controls (n = 45): MD = −200.56 pg/mL (95% CI: −223.91 to −177.21).	Vitamin B_{12} (Status vs Outcome). Autism spectrum disorder (ASD). Mixed/Others.	The ASD group showed substantially reduced vitamin B_{12} compared with controls, with lower B_{12} correlating with greater symptom severity. The authors suggest that these biochemical patterns may be involved in ASD−related mechanisms, while acknowledging that confirmatory studies are required.
**4**	Anmella et al. (2025), Spain [47]	Observational (cross−sectional; psychiatric inpatients)	N =729; (B_{12} available: N = 713); Male=287 (39.4%); Female=442 (60.6%); Age=15.1 (2.0) years; Children/Adolescents	138 (19.4%)*	575 (80.6%)*	Adjusted OR = 0.82 (95% CI: 0.72−0.93) (vitamin B_{12} insufficiency as predictor of depressive disorders vs other diagnoses; multivariable model)	Vitamin B_{12} (Status vs Outcome; insufficiency cutoff <300 pg/mL). Depressive disorders. Serotonergic.	Patients with depressive disorders showed lower vitamin B_{12} and a higher frequency of B_{12} insufficiency versus other diagnostic categories; in adjusted analyses, B_{12} insufficiency remained statistically associated with depressive disorders. The authors propose that B−vitamin insufficiency may have clinical relevance in this neurodevelopmental period and highlight the need for further research.

**5**	Boerman et al. (2016), Netherlands [48]	Observational (cross−sectional; adult outpatients)	N =320; Male=186 (58.1%); Female=134 (41.9%); Age=47 years (SD not reported); Adults	97 (30.3%)	223 (69.7%)	Vitamin D deficiency prevalence (≤12 ng/mL): 30.3% (95% CI: 25.5–35.6) (in psychiatric outpatients with bipolar disorder, schizophrenia, or schizoaffective disorder)	Vitamin D (Status vs Outcome). Schizophrenia spectrum disorders (schizophrenia + schizoaffective disorder). Dopaminergic.	Vitamin D deficiency was common in this outpatient psychiatric sample; the authors report that diagnostic category (bipolar vs schizophrenia/ schizoaffective) did not independently predict deficiency after accounting for factors such as season/ethnicity, and conclude that these patients should be considered at risk, supporting routine (e.g., annual) vitamin D assessment to mitigate somatic health risks (bone/muscle health).
**6**	Chen et al. (2024), Taiwan [49]	RCT (24−week randomized, double−blind, placebo−controlled; 2:1 allocation)	N =55; Male=29 (52.7%); Female=26 (47.3%); Age= Age not reported as mean (SD) (reported as median (IQR): 45.5 (14.0) exposed; 46.0 (25.0) comparator); Adults	36 (65.5%)	19 (34.5%)	PANSS total change (endpoint vs baseline), between−group effect size: Cohen's d = 0.01 (95% CI: −0.55 to 0.57) (computed from reported Cohen's d and group sizes; primary outcome reported as no significant between−group change over 24 weeks)	Vitamin B_{12} (Supplementation vs Placebo) (co−administered with folate 5 mg/day; B_{12} 500 µg/day). Schizophrenia. Dopaminergic.	Supplementation substantially increased serum folate and vitamin B_{12} and reduced homocysteine, but did not show a measurable benefit over placebo on psychotic symptom severity or cognitive outcomes over 24 weeks in this relatively stable schizophrenia sample; the authors highlight that larger studies and/or more symptomatic populations may be needed

								to clarify potential clinical effects.
**7**	De Koning et al. (2016), Netherlands [50]	RCT (multicenter, randomized, double−blind, placebo−controlled; 2−year)	N =2,919; Male=1,459 (50.0%); Female=1,460 (50.0%); Age reported as median (IQR): 73 (69−78) years; Adults	1,461 (50.1%)	1,458 (49.9%)	Adjusted OR = 1.13 (95% CI: 0.83−1.53) (clinically relevant depressive symptoms at 2−year follow−up; GDS−15 ≥ 5; fully adjusted logistic regression)	Vitamin B_{12} (Supplementation vs Placebo) (500 µg vitamin B_{12} + 400 µg folic acid daily; both arms received vitamin D_3 15 µg/day). Older adults with elevated homocysteine (≥12 µmol/L). Serotonergic.	Despite a greater reduction in homocysteine in the supplementation arm, two−year B_{12} + folic acid supplementation did not reduce depressive symptoms versus placebo (effect estimate near null; 95% CI includes 1.0).
**8**	Dehbokri et al. (2019), Iran [51]	RCT (double−blind randomized, placebo−controlled; adjunct to methylphenidate; 6−week intervention)	N = 96; Male= 80 (83.3%); Female= 16 (16.7%); Age= 9.2 (2.3) years; Children/Adolescents	51 (53.1%)	45 (46.9%)	Adjusted between−group MD (Vitamin D − Placebo) in Conners IA score at 6 weeks: −1.65 (95% CI: −2.98 to −0.32)	Vitamin D (Supplementation vs Placebo). Attention−deficit/hyperactivity disorder (ADHD). Dopaminergic.	Adjunctive vitamin D supplementation was associated with a greater improvement in inattention symptoms versus placebo (statistically significant adjusted between−group effect), whereas other ADHD symptom domains did not show clear between−group differences; the authors suggest vitamin D may be considered−particularly in children with low baseline vitamin D−while acknowledging study limitations.

**9**	Dhiman et al. (2021), India [52]	Observational (age−matched case−control)	N =434; Male=0 (0.0%); Female=434 (100.0%); 25.9 (4.2) years; Adults	217 (50.0%)	217 (50.0%)	Adjusted OR = 0.514 (95% CI: 0.289−0.912) for total vitamin B_{12} (log−transformed) in relation to probable postpartum depression (EPDS > 10; cases vs controls).	Vitamin B_{12} (Status vs Outcome). Probable postpartum depression (EPDS >10 at 6 weeks postpartum). Serotonergic.	Higher circulating total vitamin B_{12} was associated with lower odds of probable postpartum depression after adjustment, suggesting an inverse relationship between maternal B_{12} status and postpartum depressive symptoms in this cohort; the authors interpret low B_{12} (with related metabolic patterns) as a potential contributor in the vulnerable postpartum period, while acknowledging the observational design.
**10**	Elshorbagy et al. (2018), Egypt [53]	RCT (double−blind, randomized, placebo−controlled; adjunct to methylphenidate; 12 weeks)	N analyzed = 35 (vitamin D−deficient ADHD); Sex/Age for analyzed subset not reported; overall ADHD cohort (N = 50): Male=28 (56.0%); Female=22 (44.0%); Age=9.31 (2.60) years; Children/Adolescents	16 (45.7%)	19 (54.3%)	Conners' Parent Rating Scale−Inattention (12 weeks): MD = −16.19 (95% CI: −24.02 to −8.36) (vitamin D + methylphenidate vs placebo + methylphenidate; computed from reported follow−up mean ± SD and analyzed n per arm)	Vitamin D (Supplementation vs Placebo). Attention−deficit/hyperactivity disorder (ADHD) with vitamin D deficiency. Dopaminergic.	In vitamin D−deficient children with ADHD receiving methylphenidate, adjunctive vitamin D supplementation was associated with lower parent−rated inattention scores at follow−up versus placebo; the authors conclude these findings provide preliminary support that vitamin D supplementation may improve aspects of cognitive/behavioral

								functioning in this context, while noting limitations related to sample size and co−intervention.
**11**	Endres et al. (2016), Germany [54]	Observational (1−year inpatient cohort; tertiary care hospital)	N = 83; Male = 51 (61.4%); Female = 32 (38.6%); Age = 33.0 (11.1) years; Adults	66 (79.5%)	17 (20.5%)	Overall vitamin D deficiency (<20 ng/mL): 79.5% (95% CI: 69.6−86.8) (66/83; CI computed from observed proportion)	Vitamin D (Status vs Outcome; serum 25(OH)vitamin D). Schizophreniform and autism spectrum disorders. Mixed/Others.	Vitamin D deficiency was highly prevalent in adult inpatients with schizophreniform and autism spectrum syndromes; the authors note markedly low levels (including frequent severe deficiency) and recommend more frequent assessment of vitamin D status in these patients, while emphasizing that causality and symptom effects remain uncertain and require further controlled research.
**12**	Erensoy (2020), Turkey [55]	Observational (cross−sectional pilot study)	N = 150; Sex: not reported (male/female counts not provided); Age: not reported as mean (SD) (age range 18−79 years; age categories: 18−40 n=98; 41−60 n=47; 61−79 n=5); Adults	73 (48.7%) (depression)	77 (51.3%) (anxiety)	Serum vitamin B_{12} (pg/mL), depression vs anxiety: MD = −35.0 (95% CI: −103.1 to 33.1) (computed from group means ± SD: 321±210 vs 356±212)	Vitamin B_{12} (Status vs Outcome). Depression and anxiety disorders. Serotonergic.	Overall, vitamin B_{12} levels did not differ statistically between the depression and anxiety groups (estimate compatible with no difference). The authors suggest that assessing B_{12}/folate may be clinically informative in mood disorders, while noting that folate showed clearer

								group differences and that larger studies are needed.
**13**	Esnafoğlu & Yaman (2017), Turkey [57]	Observational (case−control; outpatient; pediatric)	N = 82; Male=40 (48.8%); Female=42 (51.2%); Age=14.5 (2.4) years; Children/Adolescents	52 (63.4%) (OCD)	30 (36.6%) (controls)	Vitamin B_{12} (pg/mL), OCD (n = 52) vs controls (n = 30): MD = −112.0 pg/mL (95% CI: −162.3 to −61.7) (computed from reported mean ± SD values)	Vitamin B_{12} (Status vs Outcome). Obsessive−compulsive disorder (OCD). Serotonergic.	Children/adolescents with OCD showed substantially lower serum vitamin B_{12} than healthy controls. The authors interpret this pattern (within one−carbon metabolism, alongside elevated homocysteine) as potentially relevant to OCD pathophysiology in youth and recommend clinical vigilance and further, larger studies (including supplementation research) to clarify causality and clinical utility.
**14**	Esnafoglu & Ozturan (2020), Turkey [56]	Observational (case−control study)	N = 132; Male=32 (24.2%); Female=100 (75.8%); Age 14.9 (1.8) years; Children/Adolescents	89 (67.4%) (depression)	43 (32.6%) (controls)	Low vitamin B_{12} (below normal range) in depression vs controls: OR = 38.28 (95% CI: 2.27−644.53) (computed from 27/89 vs 0/43 using Haldane−Anscombe correction due to a zero cell).	Vitamin B_{12} (Status vs Outcome; total cobalamin). Depressive disorder in children and adolescents. Serotonergic.	Depressed youth showed a substantially higher frequency of low vitamin B_{12} than controls; the authors interpret B_{12} insufficiency/deficiency (together with elevated homocysteine) as potentially relevant to depression pathophysiology in this age group, while noting that the cross−sectional design limits causal inference and that further

								longitudinal/interventional studies are needed.
**15**	Fabrazzo et al. (2022), Italy [58]	Observational (cross−sectional cohort: psychiatric inpatients vs stabilized outpatients)	N = 152; Male = 75 (49.3%); Female = 77 (50.7%); Age = 47.3 (14.4) years; Adults	74 (48.7%) (inpatients−acute relapse)	78 (51.3%) (outpatients−stabilized)	Serum 25(OH)D (ng/mL), inpatients (n = 74) vs outpatients (n = 78): MD (Inpatients − Outpatients) = −10.80 (95% CI: −14.64 to −6.96) (computed from reported group means ± SD)	Vitamin D (Status vs Outcome; serum 25(OH)D). Severe mental disorders (psychotic, mood, and obsessive−compulsive disorders). Mixed/Others.	Compared with stabilized outpatients, psychiatric inpatients in acute relapse had markedly lower 25(OH)D levels, supporting an association between acute decompensation and hypovitaminosis D across diagnostic categories; however, given the cross−sectional design, causality cannot be inferred and the authors recommend routine vitamin D assessment in severe mental disorders.
**16**	Ghaderi et al. (2017), Iran [59]	RCT (randomized double−blind placebo−controlled trial)	N = 68; Sex: not reported; Age = 41.3 (9.1) years; Adults	34 (50.0%)	34 (50.0%)	BDI change (Week 12 − baseline): MD (Vitamin D − Placebo) = −3.30 (95% CI: −6.56 to −0.04). Data used: total randomized sample and allocation (n = 34 per arm, ITT) and baseline age by arm (Table 1 / trial flow), plus BDI change values used to compute MD and 95% CI.	Vitamin D (Supplementation vs Placebo) (50,000 IU every 2 weeks for 12 weeks). Maintenance methadone treatment (opioid use disorder). Mixed/Others.	Vitamin D supplementation was associated with a greater reduction in depressive symptom severity (BDI) compared with placebo over 12 weeks, suggesting a modest improvement in depressive symptoms in this MMT cohort (noting that multiple primary outcomes were assessed).

**17**	Hemamy et al. (2021), Iran [60]	RCT (randomized, double–blind, placebo–controlled; 8 weeks)	N = 66; Male=46 (69.7%); Female=20 (30.3%); Age=9.11 (1.61) years; Children/Adolescents	33 (50.0%)	33 (50.0%)	SDQ Total Difficulties (adjusted mean at end of trial): MD = −3.76 (95% CI: −6.48 to −1.04) Vitamin D + magnesium vs placebo; computed from adjusted means (SE).	Vitamin D (Supplementation vs Placebo; co–supplemented with magnesium: vitamin D 50,000 IU/week + magnesium 6 mg/kg/day). Attention–deficit/hyperactivity disorder (ADHD). Mixed/Others.	Co–supplementation increased serum 25(OH)D3 and magnesium and was associated with lower overall difficulties on the SDQ versus placebo at 8 weeks, suggesting a potential short–term improvement in behavioral/mental health profiles in children with ADHD who were deficient at baseline; the authors emphasize that larger, well–designed trials are needed to confirm and generalize these findings.
**18**	Hendren et al. (2016), USA [61]	RCT (randomized, placebo–controlled; 8–week trial)	N = 57; Male = 45 (78.9%); Female = 12 (21.1%); Age = 5.3 (1.3) years; Children/Adolescents; (primary outcome analyzed: N = 50)	27 (54.0%)	23 (46.0%)	CGI–I at 8 weeks: MD = −0.70 (95% CI: −1.20 to −0.20) (methyl B_{12} vs placebo; lower score = greater clinician–rated improvement)	Vitamin B_{12} (Supplementation vs Placebo; methylcobalamin 75 µg/kg SC every 3 days). Autism spectrum disorder (ASD). Mixed/Others.	Injectable methyl B_{12} produced better clinician–rated global improvement versus placebo on the primary outcome (CGI–I), while no consistent benefits were observed on parent–rated ABC or SRS measures; the authors note that clinical improvement correlated with changes in methylation–related metabolites, suggesting potential relevance for a subset of children.

**19**	Huang et al. (2018), USA [62]	Observational (cross−sectional; NHANES 2005−2006)	N = 2,791; Male=1,443 (51.7%); Female 1,348 (48.3%); Age 49.4 (18.0) years; Adults	170 (6.1%) (Depression; PHQ−9 ≥ 10)	2,621 (93.9%) (No depression)	Adjusted OR = 1.89 (95% CI: 1.15−3.11) (depression outcome; highest vs lowest quartile of serum vitamin B_{12})	Vitamin B_{12} (Status vs Outcome). Depressive symptoms in general adult population (PHQ−9 ≥10). Serotonergic.	In the fully adjusted model, higher serum vitamin B_{12} (Q4 vs Q1) was associated with higher odds of depression; given the cross−sectional design, causality cannot be inferred and residual confounding/reverse causality remain possible.
**20**	Kaviani et al. (2022), Iran [63]	RCT (double−blind randomized, placebo−controlled; 8 weeks)	N = 56; Male = 6 (10.7%); Female = 50 (89.3%); Age = 43.0 (8.6) years; Adults	28 (50.0%)	28 (50.0%)	BDI−II change (Week 8 − baseline): MD (Vitamin D − Placebo) = −8.14 (95% CI: −12.77 to −3.51) (computed from reported change scores: −11.75 ± 6.40 vs −3.61 ± 10.40; n=28/group).	Vitamin D (Supplementation vs Placebo; cholecalciferol 50,000 IU every 2 weeks). Mild to moderate depression. Serotonergic.	Vitamin D supplementation produced a greater reduction in depressive symptom severity versus placebo over 8 weeks, alongside a marked rise in 25(OH)D; however, the authors report no significant between−group differences in the assessed pro−inflammatory biomarkers, suggesting the clinical improvement occurred independently of changes in IL−1β, IL−6, or hs−CRP within this trial.
**21**	Kerley et al. (2017), Ireland [64]	RCT (double−blind randomized placebo−controlled trial; 20 weeks)	N=42; Male=38 (90.5%); Female=4 (9.5%); Age=7.1 years (SD not reported); Children/Adolescents;	18 (47.4%)	20 (52.6%)	ABC Stereotypical Behaviour (Δ Week 20 − baseline): MD (Vitamin D − Placebo)=2.20 (95% CI: −1.87 to 6.27)	Vitamin D (Supplementation vs Placebo; vitamin D3 2000 IU/day). Autism spectrum disorder (ASD).	Vitamin D_3 supplementation substantially increased 25(OH)D levels but did not improve the prespecified primary behavioral

			(primary outcome analyzed: N=38)			(computed from mean change scores −0.8 vs −3.0 and p=0.28 for the between−group unpaired t−test; df=36)	Mixed/Others.	endpoint versus placebo; any signals in secondary/exploratory outcomes were limited and inconsistent, supporting the authors' conclusion that larger trials are warranted.
**22**	Krivoy et al. (2017), Israel [65]	RCT (randomized, double−blind, placebo−controlled; 8 weeks; adjunct in clozapine−treated chronic schizophrenia with low 25(OH)D)	N=47; Male=32 (68.1%); Female=15 (31.9%); Age=40.9 (10.4) years; Adults	24 (51.1%)	23 (48.9%)	PANSS total change (Week 8 − baseline): MD (Vitamin D − Placebo) = 1.10 (95% CI: −4.15 to 6.35) (computed from reported change scores and SDs)	Vitamin D (Supplementation vs Placebo; weekly oral vitamin D 14,000 IU). Chronic schizophrenia treated with clozapine (residual symptoms; low baseline vitamin D). Dopaminergic.	Vitamin D supplementation substantially increased serum 25(OH)D, but did not provide a clinically meaningful advantage over placebo on the prespecified primary outcome (PANSS total improvement) over 8 weeks; the authors report only a small, non−robust signal for cognition that did not persist after multiplicity correction.
**23**	Laird et al. (2023), Ireland [66]	Observational (population−representative longitudinal cohort; 4−year follow−up)	N = 3,849; Male ≈ 1,927 (50.1%); Female ≈ 1,922 (49.9%); Age ≈ 62.9 (13.4) years; Adults	389 (15.1%)	1,319 (51.0%)	Adjusted OR = 1.51 (95% CI: 1.01−2.27) (baseline deficient−low plasma vitamin B12 <185 pmol/L vs normal >258−601 pmol/L; incident depressive symptoms over 4 years)	Vitamin B_{12} (Status vs Outcome). Incident depressive symptoms in community−dwelling older adults (TILDA). Serotonergic.	Deficient−low baseline vitamin B_{12} status was associated with higher odds of incident depressive symptoms over 4 years in the fully adjusted model; the authors conclude this supports a potentially relevant (and modifiable) nutritional risk marker in older adults, while noting

								no comparable association for folate.
**24**	Libuda et al. (2020), Germany [67]	RCT (randomized, double−blind, placebo−controlled; 28 days; Treatment as Usual−TAU in both arms)	N = 113 randomized; Male = 28 (24.8%); Female = 85 (75.2%); Age = 15.9 (1.5) years; Children/Adolescents; (primary outcome analyzed: N = 100)	52 (52.0%)	48 (48.0%)	BDI−II at end of study (adjusted ANCOVA; Verum − Placebo): 1.29 (95% CI: −2.22 to 4.81)	Vitamin D (Supplementation vs Placebo; deficient at baseline). Depressive disorder / depressive symptoms (DSM−IV; K−SADS−PL; psychiatric in−/daycare patients). Serotonergic.	Immediate vitamin D_3 supplementation in vitamin D−deficient, depressed adolescent psychiatric patients did not improve self−rated depressive symptoms versus placebo over 4 weeks (primary outcome; CI includes the null). The authors note that parent−rated depressive symptoms improved in the verum group (secondary outcome), suggesting rater−dependent effects that warrant confirmation in future trials.
**25**	Madley−Dowd et al. (2022), UK [68]	Observational (prospective pregnancy cohort; ALSPAC; 1991−1992 births)	N = 7,689; Offspring male = 4,001 (52.0%); female = 3,688 (48.0%); Maternal age at delivery = 28.1 (4.8) years; Adults	93 (1.2%) (offspring autism diagnosis)	7,596 (98.8%) (no autism diagnosis)	Adjusted OR = 0.99 (95% CI: 0.93−1.06) per 10 nmol/L increase in seasonally− and gestational age−adjusted maternal serum 25(OH)D during pregnancy (offspring autism diagnosis; multiple imputation analysis).	Vitamin D (Status vs Outcome; maternal serum 25(OH)D in pregnancy). Autism spectrum disorder (ASD). Mixed/Others.	Maternal 25(OH)D levels during pregnancy showed no strong evidence of association with offspring autism diagnosis (effect estimate close to null; CI includes 1.0); Mendelian randomization analyses likewise did not support a causal effect, with limited power noted.

**26**	Marsh et al. (2017), USA [69]	RCT (double−blind, placebo−controlled; 12 weeks)	N randomized = 33; Male = 17 (51.5%); Female = 16 (48.5%); Age = 44.2 (13.1) years; Adults; (primary outcome analyzed: N = 25)	13 (52.0%)	12 (48.0%)	MADRS (mixed−effects; time × treatment interaction): 0.29 (95% CI: −4.00 to 4.58), p = 0.89 (no between−group difference in depressive symptom trajectory over 12 weeks)	Vitamin D (Supplementation vs Placebo; vitamin D3 5000 IU/day; baseline deficiency). Bipolar depression (DSM−IV bipolar I/II/NOS; vitamin D deficient <30 ng/mL). Mixed/Others.	Vitamin D_3 increased serum 25(OH)D more than placebo, but did not yield greater improvement in bipolar depressive symptoms versus placebo; both groups showed symptom reduction (high placebo response), and mean vitamin D status remained deficient on average, leading the authors to suggest that larger and/or longer trials (potentially achieving sufficiency) are needed to clarify clinical benefit.
**27**	Mazahery et al. (2019), New Zealand [70]	RCT (randomized, double−blind, placebo−controlled; 2×2 factorial with omega−3)	N randomized = 117; Male = 100 (85.5%); Female = 17 (14.5%); Age = 5.2 (1.4) years; Children/Adolescents	19 (54.3%) (Vitamin D arm, completers for SRS)	16 (45.7%) (Placebo, completers for SRS)	SRS Total (change, endpoint − baseline): Adjusted MD (Vitamin D − Placebo) = −6.3 (95% CI: −31.0 to 8.2)	Vitamin D (Supplementation vs Placebo; vitamin D3 2000 IU/day). Autism spectrum disorder (ASD; DSM−5 confirmed). Mixed/Others.	Vitamin D supplementation did not show a statistically significant advantage over placebo on the prespecified core ASD symptom outcome (SRS total) in the adjusted completer analysis (CI includes the null), indicating no clear evidence of benefit for vitamin D alone in this trial.
**28**	Misal et al. (2024), India [71]	RCT (prospective randomized trial; open−label, control	N = 62; Male = 46 (74.2%); Female = 16 (25.8%);	32 (51.6%)	. 30 (48.4%)	Remission at day 28 (HAM−D < 11): OR = 4.08 (95% CI:	Vitamin B_{12} (Supplementation vs Control, no	In older adults with low−normal baseline B_{12} receiving standard

		without placebo; 4 weeks)	Age = 66.4 (6.0) years; Adults (low−normal baseline serum B_{12}: 187−350 pg/mL)			1.35−12.30) (computed from 25/32 vs 14/30, as reported in Results/Discussion)	placebo; IM injections as augmentation to antidepressant). Late−life depression (ICD−10; moderate−to−severe; non−psychotic). Serotonergic.	antidepressants, adjunctive parenteral vitamin B_{12} was associated with higher short−term remission rates by 4 weeks versus antidepressant treatment alone; however, the authors note that the open−label design, small sample, and short follow−up limit causal inference and call for larger controlled studies.
**29**	Mohammadpour et al. (2018), Iran [72]	RCT (randomized, double−blind, placebo−controlled; adjunct to methylphenidate; 8 weeks)	N randomized = 62; Male = 46 (74.2%); Female = 16 (25.8%); Age = 7.87 (1.61) years; Children/Adolescents; (primary outcome analyzed: N = 54)	25 (46.3%)	29 (53.7%)	CPRS ADHD Index (Week 8, endpoint): MD (Vitamin D − Placebo) = −0.95 (95% CI: −6.26 to 4.36) (computed from reported Week−8 means ± SD; n=25 vs 29)	Vitamin D (Supplementation vs Placebo; 2000 IU/day) + methylphenidate; serum 25(OH)D monitored. Attention deficit hyperactivity disorder (ADHD; DSM−IV). Mixed/Others.	On the prespecified Conners' parent−rated ADHD symptom outcomes (including the ADHD Index), vitamin D did not demonstrate a clear advantage over placebo (between−group estimate near null; CI includes 0). The authors report the main signal on evening behavior symptoms (WPREMB) rather than core CPRS/ADHD−RS scores.
**30**	Naeini et al. (2019), Iran [73]	RCT (randomized, double−blind, placebo−controlled; 3 months)	N = 71; Male = 59 (83.1%); Female = 12 (16.9%); Age = 9.12 (1.55) years; Children/Adolescents	36 (50.7%)	35 (49.3%)	CPQ total score (post−intervention): MD (Vitamin D − Placebo) = −11.72 (95% CI: −19.13 to −4.31) (computed	Vitamin D (Supplementation vs Placebo; vitamin D_3 1000 IU/day). Attention deficit hyperactivity	Vitamin D supplementation was associated with lower parent−rated ADHD symptom severity versus placebo at 3 months

						from post means ± SD: 65.97±14.50 vs 77.69±16.66)	disorder (ADHD; DSM−IV). Mixed/Others.	(clinically favorable direction). The authors report broader improvements across parent/teacher behavioral scales and reduced impulsivity, while some cognitive indices (e.g., attention/reaction time) did not show clear between−group change.
**31**	Okasha et al. (2020), Egypt [74]	Observational (cross−sectional comparative case−control; 3 groups: MDD vs schizophrenia vs healthy controls)	Total N = 60 (MDD n=20; schizophrenia n=20; controls n=20); Sex: not reported (male/female counts not provided); Age = 29.0 (6.3) years; Adults	20 (50.0%)	20 (50.0%)	Serum 25(OH)D (ng/mL), MDD vs controls: MD (MDD − Control) = −20.60 (95% CI: −33.44 to −7.76) (computed from group means ± SD: 18.2±12.2 vs 38.8±25.2)	Vitamin D (Status vs Outcome; serum 25(OH)D). Major depressive disorder (MDD); Schizophrenia. Mixed/Others.	Patients with MDD showed substantially lower serum vitamin D than healthy controls (CI excludes 0). The authors also report low vitamin D in schizophrenia versus controls, but vitamin D levels did not clearly differentiate MDD from schizophrenia in this sample.
**32**	Okereke et al. (2020), USA [75]	RCT (double−blind, randomized, placebo−controlled; 2×2 factorial; median follow−up 5.3 years)	N=18,353; Male=9,330 (50.8%); Female=9,023 (49.2%); Age=67.5 (7.1) years; Adults	9,181 (50.0%)	9,172 (50.0%)	HR = 0.97 (95% CI: 0.87−1.09) (vitamin D3 2000 IU/day vs placebo; risk of depression or clinically relevant depressive symptoms over follow−up)	Vitamin D (Supplementation vs Placebo). Depression or clinically relevant depressive symptoms (prevention; baseline PHQ−8 <10). Serotonergic.	Long−term vitamin D_3 supplementation did not reduce the incidence/recurrence of depression or clinically relevant depressive symptoms compared with placebo (effect estimate close to null; CI includes 1.0); the authors conclude these results do not support

								vitamin D3 use for depression prevention in this population.
**33**	Omidian et al. (2019), Iran [76]	RCT (randomized, double−blind, placebo−controlled; 12 weeks)	N=66; Male=39 (59.1%); Female=27 (40.9%); Age=50.5 (6.2) years; Adults (computed on N total from group summaries)	32 (48.5%)	34 (51.5%)	BDI−II change (baseline→12 weeks): MD (Vitamin D − Placebo) = −3.60 points (95% CI: −6.61 to −0.59) (derived from reported mean decreases 5.4 vs 1.8 and between−group p=0.02)	Vitamin D (Supplementation vs Placebo; 4000 IU/day; serum 25(OH)D increased). Type 2 diabetes mellitus with mild−to−moderate depressive symptoms (BDI−II Persian). Serotonergic.	Vitamin D supplementation was associated with a greater reduction in depressive symptom severity than placebo over 12 weeks in T2DM patients with mild−moderate depressive symptoms and vitamin D deficiency; the authors additionally note favorable shifts in selected metabolic markers alongside the mood−related outcome.
**34**	Penckofer et al. (2022), USA [77]	RCT (randomized, double−blind, active comparator−controlled; 6 months)	N = 129; Male = 0 (0.0%); Female = 129 (100.0%); Age = 50.6 (11.1) years; Adults	65 (50.4%) (vitamin D_3 50,000 IU/week)	64 (49.6%) (vitamin D_3 5,000 IU/week; no placebo)	CES−D at 6 months (baseline−adjusted mixed model): Adjusted MD (High dose − Low dose) = 1.33 (95% CI: −2.06 to 4.72)	Vitamin D (Supplementation vs Control, no placebo; cholecalciferol 50,000 IU/week vs 5,000 IU/week). Depressive symptoms in women with type 2 diabetes (CES−D screening; 25(OH)D <32 ng/mL). Serotonergic.	There was no evidence of a dose−related advantage of higher−dose versus lower−dose vitamin D_3 for reducing depressive symptoms; both groups showed substantial improvement over time, with between−group differences remaining non−significant.
**35**	Petruzzelli et al. (2020), Italy [78]	Observational (case−control;	N=90;	54	36 (40.0%)	Adjusted OR = 10.31 (95% CI: 1.96–54.22)	Vitamin D (Status vs Outcome; serum	Children with ASD had a higher likelihood of

		cross−sectional; inpatient chart review)	Male=65 (72.2%); Female=25 (27.8%); Age=8.6 (4.7) years; Children/Adolescents	(60.0%) (ASD)	(non−ASD controls)	(vitamin D deficiency <20 ng/mL vs normal 30−100 ng/mL; association with ASD; age− and sex−adjusted logistic regression)	25(OH)D; deficiency cutoff <20 ng/mL). Autism spectrum disorder (ASD; DSM−5; ADI−R/ADOS−2 supported). Mixed/Others.	vitamin D deficiency and lower mean 25(OH)D levels than the non−ASD clinical control group; the authors interpret this as a potentially relevant association (with unclear directionality) and recommend more frequent vitamin D assessment in ASD while calling for prospective studies to clarify causality and therapeutic implications.
**36**	Rahman et al. (2023), Australia [79]	RCT (D−Health Trial; randomized double−blind placebo−controlled; monthly vitamin D3 vs placebo; up to 5 years)	N = 20,487; Male = 11,082 (54.1%); Female = 9,405 (45.9%); Age = 69.3 (SD NR) years; Adults	10,270 (50.1%)	10,217 (49.9%)	PHQ−9 score (overall): MD = 0.02 (95% CI: −0.06 to 0.11) (vitamin D vs placebo)	Vitamin D (Supplementation vs Placebo). Clinically relevant depression / depressive symptoms (PHQ−9). Serotonergic.	In this large, long−term trial, high−dose monthly vitamin D_3 did not improve depressive symptoms overall versus placebo (effect estimate ~0 with CI spanning the null). The authors report only limited subgroup signals, which they interpret cautiously.
**37**	Rouhi et al. (2018), Iran [80]	RCT (randomized, double−blind, placebo−controlled; 6 months)	N=80; Male=0 (0.0%); Female=80 (100.0%); Age=24.7 (3.1) years; Adults	. 40 (50.0%)	40 (50.0%)	Edinburgh Postnatal Depression Scale (EPDS) at 6 months (endpoint): MD (Vitamin D − Placebo) = −4.80 (95% CI: −6.81 to −2.79) (computed from endpoint means ±	Vitamin D (Supplementation vs Placebo; vitamin D_3 1000 IU/day). Postpartum depression / fatigue risk (EPDS ≥13 and FIF ≥20 at baseline; primiparous	In postpartum women with elevated baseline symptom scores (EPDS ≥13; fatigue threshold met), daily vitamin D3 for 6 months was associated with lower depressive symptom scores versus placebo at follow−up, consistent with

						SD: 8.6±4.3 vs 13.4±4.7; n=40/group)	postpartum women). Serotonergic.	a beneficial effect in this high−risk sample; however, the study did not measure baseline serum vitamin D and outcomes were based on self−report scales, so generalizability and mechanistic interpretation remain limited.
**38**	Shahini et al. (2022), Iran [81]	Observational (case−control study; schizophrenia vs healthy controls; 1:1 matched on age and sex)	N = 66; Male = 50 (75.8%); Female = 16 (24.2%); Age: median (IQR) reported − cases 40 (34−47) vs controls 39 (28.5−49.5) years (as reported); Adults	33 (50.0%) (schizophrenia)	33 (50.0%) (controls)	Vitamin D deficiency (<10 μg/dL): OR = 2.30 (95% CI: 0.80−6.61)	Vitamin D (Status vs Outcome; serum vitamin D level/deficiency). Schizophrenia (DSM−5 confirmed by psychiatrist). Dopaminergic.	Serum vitamin D levels were lower in schizophrenia cases than in controls (median difference significant in the study's group comparison), while the odds of categorical deficiency were higher but imprecisely estimated (CI includes 1.0). Overall, the authors interpret hypovitaminosis D as common and potentially clinically relevant in schizophrenia, without supporting causal inference from this design.
**39**	Sourander et al. (2021), Finland [82]	Observational (nationwide register−based nested case−control, 1:1 matched by sex and date of birth)	N=3,116; Male=2,508 (80.5%); Female=608 (19.5%); Age at index/first ASD diagnosis=6.58 (3.21) years; Children/Adolescents	1,558 (50.0%) (ASD cases)	1,558 (50.0%) (matched controls)	Adjusted OR = 1.52 (95% CI: 1.20−1.91) for maternal 25(OH)D deficiency <30 nmol/L vs ≥50 nmol/L (offspring ASD)	Vitamin D (Status vs Outcome; maternal serum 25(OH)D in early pregnancy). Autism spectrum disorder (ASD;	Low maternal vitamin D status in early pregnancy was associated with higher odds of offspring ASD after adjustment, supporting a potentially relevant prenatal risk marker; the

							ICD−9/ICD−10 registry diagnosis). Mixed/Others.	authors emphasize the observational design and the possibility of residual confounding despite extensive covariate control.
**40**	Terock et al. (2020), Germany [83]	Observational (population−based cross−sectional analysis; SHIP−1; logistic regression in trauma−exposed subsample)	N = 1,653; Male = 831 (50.3%); Female = 822 (49.7%); Age: NR as mean (SD) (reported as median [IQR]: 59.0 [45.0–70.0] years in non−PTSD vs 56.0 [45.0–71.0] years in PTSD); Adults	63 (3.8%) (PTSD)	1,590 (96.2%) (no PTSD)	OR = 0.96 (95% CI: 0.93–0.99) per 1 ng/mL increase in serum 25(OH)D (PTSD; fully adjusted model).	Vitamin D (Status vs Outcome; serum total 25(OH)D). Posttraumatic stress disorder (PTSD; DSM−IV SCID module). Mixed/Others.	Higher serum 25(OH)D levels were inversely associated with PTSD odds after multivariable adjustment (small effect size; CI excludes 1.0). The authors emphasize that causality/direction cannot be inferred from the cross−sectional design and note attenuation after additional adjustment for trauma load, suggesting potential residual confounding or trauma−related mediation.
**41**	Van der Leeuw et al. (2020), Netherlands [84]	Observational (cross−sectional case−control; GROUP cohort: psychotic disorder vs controls)	N=629; Male=386 (61.4%); Female=243 (38.6%); Age=32.2 (8.9) years; Adults	347 (55.2%) (psychotic disorder)	282 (44.8%) (controls)	Adjusted β = −8.05 nmol/L (95% CI: −13.68 to −2.42) for plasma 25(OH)D3 concentration in patients vs controls	Vitamin D (Status vs Outcome; plasma 25(OH)D3). Psychotic disorder (non−affective psychosis; DSM−IV CASH; schizophrenia/ schizoaffective/ schizophreniform). Dopaminergic.	Patients with psychotic disorder had significantly lower vitamin D concentrations than controls after multivariable adjustment, supporting an inverse association between vitamin D status and psychotic disorder; given the cross−sectional design, the authors emphasize that

								directionality/causality cannot be established.
**42**	Vaziri et al. (2016), Iran [85]	RCT (double−blind, placebo−controlled trial)	N = 153 pregnant women (26−28 weeks' gestation; baseline 25(OH)D measured); Male = 0 (0.0%); Female = 153 (100.0%); Age = 26.3 (4.6) years; Adults	64 (47.1%)	72 (52.9%)	EPDS score at 8 weeks postpartum (Vitamin D − Placebo): MD = −2.99 (95% CI: −4.29 to −1.69) (computed from reported group means/SDs)	Vitamin D (Supplementation vs Placebo; serum 25(OH)D context). Perinatal depressive symptoms prevention (pregnant women EPDS baseline 0−13). Serotonergic.	Compared with placebo, vitamin D3 (2,000 IU/day in late pregnancy) was associated with lower postpartum depressive symptom scores at the 8−week follow−up, consistent with a clinically meaningful reduction in EPDS levels in the supplemented group, while acknowledging outcome−specific attrition at follow−up.
**43**	Vellekkatt et al. (2020), India [86]	RCT (double−blind, randomized, placebo−controlled; adjunct to TAU; 12 weeks; single IM dose at baseline)	N=46; Male=15 (32.6%); Female=31 (67.4%); Age=35.9 (11.6) years; Adults; all had vitamin D deficiency at baseline (serum 25(OH)D <20 ng/mL)	23 (50.0%)	23 (50.0%)	HDRS−17 change (baseline→12 weeks; ANCOVA adjusted for baseline): F(1,44)=11.55, p=0.001; partial η^2=0.21	Vitamin D (Supplementation vs Placebo; single parenteral cholecalciferol 300,000 IU + TAU vs saline placebo + TAU). Major depressive disorder (MDD; DSM−5; MINI−Plus confirmed) with concurrent vitamin D deficiency (25(OH)D <20 ng/mL). Serotonergic.	Adjunctive single−dose parenteral vitamin D given at baseline was associated with greater improvement in clinician−rated depressive symptoms over 12 weeks versus placebo (primary outcome), with additional reported gains in quality of life and global clinical severity measures; findings are framed as supportive of targeted supplementation in MDD with confirmed vitamin D deficiency.

**44**	Yazici et al. (2019), Turkey [87]	Observational (retrospective, cross−sectional; schizophrenia vs substance use disorder vs healthy controls)	N=412; Male=282 (68.4%); Female=130 (31.6%); Age=37.55 (13.81) years; Adults	189 (64.5%)	104 (35.5%)	OR = 6.40 (95% CI: 3.29−12.45) for vitamin B_{12} deficiency (≤200 ng/mL) in schizophrenia vs healthy controls	Vitamin B_{12} (Status vs Outcome). Schizophrenia. Dopaminergic.	Vitamin B_{12} deficiency was substantially more frequent in schizophrenia than in healthy controls, indicating a strong cross−sectional association between low B_{12} status and schizophrenia in this sample; the authors emphasize that, given the retrospective design and clinical database sourcing, causality and directionality cannot be inferred.
**45**	Yee et al. (2016), Singapore [88]	Observational (case−control; first−episode psychosis vs matched healthy controls)	N=62; Male=29 (46.8%); Female=33 (53.2%); Age=29.0 (9.7) years; Adults	31 (50.0%) (FEP)	31 (50.0%) (controls)	MD = −2.63 nmol/L (95% CI: −5.76 to 0.50) for bioavailable vitamin D (FEP − controls), computed from reported group means/SDs	Vitamin D (Status vs Outcome; bioavailable vitamin D, with DBP context). First−episode psychosis (FEP; DSM−IV Axis I; SCID−I). Dopaminergic.	The FEP group showed lower bioavailable vitamin D than controls (while total vitamin D did not differ materially), suggesting that bioavailable vitamin D may be a more sensitive status marker in early psychosis; the authors additionally report an inverse relationship between bioavailable vitamin D and negative symptom severity in adjusted models.
**46**	Yektaş et al. (2019), Turkey [89]	Observational (single−center case−control; 3 groups: ADHD vs ASD vs healthy controls)	N=118; Male=96 (81.4%); Female=22 (18.6%); Age: median (IQR) reported − overall median 8.0 (3.0) years	35 (50.0%) (ASD)	35 (50.0%) (healthy controls)	Serum vitamin B_{12} (median (IQR), pg/mL): ASD 268 (407) vs controls 1611 (357); $p < 0.001$ (group effect and	Vitamin B_{12} (Status vs Outcome). ADHD; ASD. Mixed/Others.	Children with ASD (and ADHD) showed markedly lower vitamin B_{12} (and higher homocysteine) than controls, supporting an association between altered

	(as reported); Children/Adolescents	post–hoc comparisons as reported)	one–carbon–related biomarkers and neurodevelopmental disorders; however, the cross–sectional design precludes causal inference, and symptom–biomarker correlations were observed mainly within ADHD (not ASD), so the authors call for larger studies to clarify etiologic and clinical relevance.

Notes:

- Age is reported as mean (SD) when available; otherwise as reported in the original study (e.g., median (IQR) or age categories).
- When biomarker measurements were not available for the full study sample, Exposed/Comparator counts and percentages were calculated using the biomarker–available subsample (N_available), and this denominator is explicitly reported in the Population cell (e.g., “B_{12} available: N = …”) to ensure transparency and avoid misinterpretation.
- 4–PA = 4–pyridoxic acid;
- 25(OH)D = 25–hydroxyvitamin D;
- ABC = Aberrant Behavior Checklist;
- ADHD = Attention Deficit Hyperactivity Disorder;
- ADHD–RS = ADHD Rating Scale–IV;
- ADI–R = Autism Diagnostic Interview–Revised;
- ADOS–2 = Autism Diagnostic Observation Schedule, Second Edition;
- ALSPAC = Avon Longitudinal Study of Parents and Children;
- AMDP = Arbeitsgemeinschaft für Methodik und Dokumentation in der Psychiatrie;
- ANCOVA = Analysis of covariance;
- ARS = Childhood Autism Rating Scale;
- ASD = Autism Spectrum Disorder;
- ATC = Anatomical Therapeutic Chemical;
- BDI–II = Beck Depression Inventory–II;

- BDI−II−PERSIAN = Beck Depression Inventory−II Persian version;
- BMI = Body Mass Index;
- bpm = Beats per Minute;
- CAGE = Cut down, Annoyed, Guilty, Eye−opener questionnaire;
- CASH = Comprehensive Assessment of Symptoms and History;
- cB_{12} = Combined B_{12} Score;
- CES−D−8 = 8−item Center for Epidemiological Studies Depression Scale;
- CES−D−20 = 20−item Center for Epidemiological Studies Depression Scale;
- CDS = Calgary Depression Scale;
- CGI−I = Clinical Global Impressions−Improvement;
- CGI−S = Clinical Global Impression−Severity;
- CLIA = Chemiluminescence Immunoassay;
- CPQ = Conners Parent Questionnaire;
- CPT = Continuous Performance Test;
- DHA = Docosahexaenoic Acid;
- DISYPS−DES = Diagnostic System for Mental Disorders in Childhood and Adolescence, Depression Scale;
- DSM−IV−TR = Diagnostic and Statistical Manual of Mental Disorders, Fourth Edition, Text Revision;
- eGFR = Estimated Glomerular Filtration Rate;
- EQ−5D = EuroQol 5 Dimensions;
- ELISA = Enzyme−linked Immunosorbent Assay;
- EPA = Eicosapentaenoic Acid;
- EPDS = Edinburgh Postnatal Depression Scale;
- FA = Folic Acid;
- FBS = Fasting Blood Sugar;
- fGSH = Free Glutathione;
- FIF = Fatigue Identification Form;
- FiPS−A = Finnish Prenatal Study of Autism and Autism Spectrum Disorder;
- FMC = Finnish Maternity Cohort;
- FR = Food Record;
- GDS−15 = Geriatric Depression Scale (15−item);
- GDS−H = Geriatric Depression Scale Hindi version;
- GEE = Generalized Estimating Equations;

- G.R.O.U.P. = Genetic Risk and Outcome of Psychosis;
- GSH = Glutathione;
- GSSG = Oxidized Glutathione Disulfide;
- GWAS = Genome−wide Association Study;
- HAM−A = Hamilton Anxiety Rating Scale;
- HAM−D = Hamilton Rating Scale for Depression;
- HbA1c = Glycated Haemoglobin;
- Hcy = Homocysteine;
- HDL = High−Density Lipoprotein;
- HDRS−17 = Hamilton Depression Rating Scale−17;
- H/I = Hyperactivity/Impulsivity;
- HMSE = Hindi Mental Status Examination;
- HoloTC = Holotranscobalamin;
- HPLC = High−Performance Liquid Chromatography;
- HR−QoL = Health−Related Quality of Life;
- hs−CRP = High−Sensitivity C−Reactive Protein;
- IA = Inattention;
- ICD−10 = International Statistical Classification of Diseases and Related Health Problems, 10th Revision;
- IL−1β = Interleukin−1β;
- IL−6 = Interleukin−6;
- IPAQ = International Physical Activity Questionnaire;
- iPTH = Intact Parathyroid Hormone;
- IQ = Intelligence Quotient;
- IU = International Units;
- JIPMER = Jawaharlal Institute of Post Graduate Medical Education and Research;
- K−SADS−PL = Kiddie Schedule for Affective Disorders and Schizophrenia for School−Aged Children − Present and Lifetime Version;
- LC−MS/MS = Liquid Chromatography−Tandem Mass Spectrometry;
- LDL = Low−Density Lipoprotein;
- LCPUFA = Long Chain Polyunsaturated Fatty Acids;
- LLD = Late−Life Depression;
- lowerOR = Lower Bound of the 95% Confidence Interval for the Odds Ratio;
- MADRS = Montgomery−Åsberg Depression Rating Scale;

- MAO−A = Monoamine Oxidase A;
- MET = Metabolic Equivalent Task;
- MDD = Major Depressive Disorder;
- mg/dL = Milligrams per Decilitre;
- M.I.N.I. = Mini−International Neuropsychiatric Interview;
- MMA = Methylmalonic Acid;
- MMSE = Mini−Mental State Examination;
- MoCA = Montreal Cognitive Assessment;
- MR = Mendelian Randomization;
- ng/mL = Nanograms per Millilitre;
- NHANES = National Health and Nutrition Examination Survey;
- nmol/L = Nanomoles per Litre;
- NNT = Number Needed to Treat;
- OM = Omega−3 Long Chain Polyunsaturated Fatty Acids Arm;
- OR = Odds Ratio;
- PANSS = Positive and Negative Syndrome Scale;
- PBS = Pharmaceutical Benefits Scheme;
- PDD = Pervasive Developmental Disorder;
- PDD−NOS = Pervasive Developmental Disorder−not Otherwise Specified;
- PHQ−8−9 = 8−9−item Patient Health Questionnaire;
- PLP = Pyridoxal 5′−phosphate;
- PTH = Parathyroid Hormone;
- PTSD = Posttraumatic Stress Disorder;
- Q−LES−Q−SF = Quality of Life Enjoyment and Satisfaction Questionnaire−Short Form;
- RAAS = Renin−Angiotensin−Aldosterone System;
- RCT = Randomized Controlled Trial;
- ROC = Receiver−Operating Characteristic;
- SAH = S−adenosyl−L−homocysteine;
- SAM = S−adenosylmethionine;
- SAS = Simpson−Angus Extrapyramidal Side Effects Scale;
- SCID = Structured Clinical Interview for the Diagnostic and Statistical Manual of Mental Disorders;
- SCQ = Social Communication Questionnaire;

- SD = Standard Deviation;
- SDQ = Strengths and Difficulties Questionnaire;
- SDQP = Strengths and Difficulties Questionnaire Parent Version;
- SDQT = Strengths and Difficulties Questionnaire Teacher Version;
- SHIP = Study of Health in Pomerania;
- SNP = Single Nucleotide Polymorphism;
- SNRI = Serotonin Norepinephrine Reuptake Inhibitor;
- SPM = Sensory Processing Measure;
- SRS = Social Responsiveness Scale;
- SSRI = Selective Serotonin Reuptake Inhibitor:
- tGSH = Total Glutathione;
- T2DM = Type 2 Diabetes Mellitus;
- TAU = Treatment as Usual;
- TILDA = The Irish Longitudinal Study on Ageing;
- TG = Triglycerides;
- TPH2 = Tryptophan Hydroxylase 2;
- upperOR = Upper Bound of the 95% Confidence Interval for the Odds Ratio;
- VDBP = Vitamin D−Binding Protein;
- VDR = Vitamin D Receptor;
- VID = Vitamin D_3 Arm;
- VIDOM = Combined Vitamin D_3 plus omega−3 LCPUFA Arm;
- VITAL = Vitamin D and Omega−3 Trial;
- VITAL−DEP = Vitamin D and Omega−3 Trial−Depression Endpoint Prevention;
- WPREMB = Weekly Parent Ratings of Evening and Morning Behavior;
- YMRS = Young Mania Rating Scale;

Table S5. Risk–of–Bias (RoB 2) appraisal for randomized controlled trials and Certainty–of–Evidence ratings under the GRADE approach.

ID	Study	Domain 1 Randomization Process (Judgment & Justification)	Domain 2 Deviations from Intended Interventions (Judgment & Justification)	Domain 3 Missing Outcome Data (Judgment & Justification)	Domain 4 Measurement of the Outcome (Judgment & Justification)	Domain 5 Selection of the Reported Result (Judgment & Justification)	Overall Risk of Bias 2 (Judgment & Justification)	GRADE Certainty of Evidence (Rating & Rationale)
**1**	Allott et al., (2019) [45]	Low (Adequate randomization and allocation concealment; baseline balance acceptable)	Low (Double–blind; adherence monitored; no systematic deviations reported)	Some concerns (Incomplete availability for certain secondary domains; limited intention to treat handling for all cognitive subtests)	Low (Validated instruments; masked assessors; consistent procedures)	Low (Prespecified outcomes; comprehensive tables; no selective reporting detected)	Some concerns (Minor attrition/availability issues in secondary domains)	Moderate (RCT evidence with imprecision across several secondary endpoints; domain–specific signal in attention/ vigilance)
**2**	Chen et al., (2024) [49]	Low (Computer–generated allocation in blocks; allocation concealment via research pharmacist; double–blind)	Low (Masked participants, caregivers, and assessors; identical capsules; no systematic deviations; biomarker changes	Low (CONSORT flow indicates analyzed *n* = 36 vs. *n* = 19 with GEE on repeated measures; missingness limited and handled)	Low (PANSS administered by trained, masked assessor; Cogstate validated in schizophrenia; standardized protocols)	Low (Trial registered; prespecified outcomes reported consistently; figures/tables align)	Low (Robust randomization/ blinding, appropriate analysis; minor imprecision due to small sample size)	Moderate (Downgraded one level for imprecision (n = 55, wide CIs around null); otherwise direct RCT evidence with appropriate conduct)

			corroborate adherence)					
3	De Koning et al., (2016) [50]	Low (Computer−generated allocation; stratified randomization; allocation concealment described)	Low (Double−blind; identical tablets; adherence high)	Low (Dropout ≈ 14–15%; similar by arm; outcomes analyzed with appropriate models; ITT and per−protocol consistent)	Low (Validated instruments − GDS−15, SF−12, EQ−5D; blinded assessment)	Low (Pre−specified outcomes; full reporting; statistical plan aligned with trial design)	Low (Low risk across domains in a large multicenter RCT)	Moderate (Downgraded one level for imprecision/ indirectness on depressive symptoms (low event rate; secondary outcome); otherwise robust trial methods)
4	Dehbokri et al., (2019) [51]	Some concerns (Block randomization reported; allocation concealment not fully detailed; baseline age imbalance noted)	Low (Double−blind; indistinguishable placebo; concomitant methylphenidate unchanged; adherence inferred from biomarker rise)	Low (96/102 completed; similar retention by arm; outcomes analyzed on completers with prespecified tests)	Low (CPRS administered under blinded conditions; validated instrument; consistent timing)	Low (Trial registered; outcomes and analyses reported as planned)	Some concerns (Randomization reporting limited; otherwise low risk across remaining domains)	Low (Downgraded for imprecision − small sample, wide Cis − and some concerns in randomization reporting; direct, randomized evidence otherwise consistent with outcome direction)
5	Elshorbagy et al., (2018) [53]	Some concerns (Patients with vitamin D−deficient ADHD were stratified by gender and randomized 1:1 to vitamin D vs placebo using permuted−block randomization, but	Low (Double−blind design; vitamin D and placebo identical in appearance; all participants received the same co−intervention	Some concerns (Attrition was 20% in the vitamin D group vs 5% in placebo; reasons for drop−out are partly	Low (Outcomes − Conners' Parent Rating Scale, WCST − are validated instruments applied	Some concerns (Primary and key secondary outcomes are reported consistently with stated aims, but no pre−registered protocol is	Some concerns (Overall, a small underpowered RCT with differential attrition and limited reporting of some details, but no major bias from	Low (RCT evidence, but downgraded for risk of bias − some concerns in randomization and missing data − imprecision − small sample, no formal sample size calculation,

		details on sequence generation and allocation concealment are not fully described)	(methylphenidate and behavioral therapy); adherence monitored by pill counts and 25(OH)D levels, with no evidence of differential protocol deviations)	reported and attrition is modest, but the small sample and imbalance between arms may bias effect estimates; no explicit ITT analysis is presented)	similarly in both groups)	available and selective reporting of some secondary or safety endpoints cannot be excluded)	deviations from intended interventions or outcome measurement)	wide Cis – and indirectness – vitamin D tested only as add–on to methylphenidate rather than monotherapy; findings considered preliminary)
**6**	Ghaderi et al., (2017) [59]	Some concerns (Randomization using computer–generated random numbers is described, but detailed information on allocation concealment procedures is not reported; baseline anthropometric and clinical characteristics (for example, age, body mass index and methadone dosage) appear broadly similar between groups, which partially mitigates but does not fully eliminate concerns about the	Low (The study used a double–blind design with matching placebo capsules, and participants, clinicians and outcome assessors were kept unaware of allocation; adherence to supplements was high (> 90% of capsules taken) and verified through changes in serum 25–hydroxyvitamin D [25(OH)D],	Low (Eight participants – four in the vitamin D_3 group and four in the placebo group – were lost to follow–up for personal reasons, but all sixty–eight randomized patients were included in the intention–to–treat – ITT – analyses; attrition was balanced	Low (Psychological outcomes were measured with validated instruments – Beck Depression Inventory, BDI – Beck Anxiety Inventory, BAI and Pittsburgh Sleep Quality Index, PSQI, and biochemical markers were assessed using standardized laboratory assays; participants and assessors were	Low (The Iranian Registry of Clinical Trials protocol prespecified psychological and metabolic outcomes at 12 weeks, and the published article reports the main endpoints – depressive symptoms, anxiety, sleep quality, glycaemic indices, lipid profile, inflammatory and oxidative stress	Some concerns (Overall risk of bias is judged as having some concerns, driven primarily by incomplete reporting of allocation concealment within the randomization process; other domains, including deviations from intended interventions, missing outcome data, outcome measurement and selection of reported results,	Moderate (Evidence for the effect of vitamin D_3 supplementation on depressive symptoms in adults receiving maintenance methadone treatment, MMT, derives from a randomized, double–blind, placebo–controlled clinical trial with intention–to–treat, ITT, analysis showing an odds ratio of approximately 2.4 for improvement on the Beck Depression Inventory. Certainty is downgraded one level

		randomization process)	with no evidence that deviations from intended interventions were related to prognostic factors or to knowledge of the assigned intervention)	across arms and unlikely to introduce clinically important bias in estimates of treatment effect)	blinded to group allocation, reducing the risk of differential outcome assessment)	markers – at the planned time point with appropriate statistical analyses; there is no indication of selective outcome reporting or data–driven selective emphasis.	are at low risk of bias)	for imprecision – modest total sample size and relatively wide confidence intervals around the odds ratio, with the lower bound close to 1.0 – and one level for some concerns regarding the randomization process, while other domains are robust)
**7**	Hemamy et al., (2021) [60]	Low (Children with attention–deficit/hyper activity disorder (ADHD) were randomly allocated to intervention or placebo using a computer–generated sequence with stratification by gender and a double–block method; allocation concealment was maintained through sequentially numbered, sealed, opaque envelopes prepared by an independent researcher, and	Low (The trial was explicitly double–blind, with participants, parents, clinicians and outcome assessors unaware of group assignment; vitamin D and magnesium supplements and their respective placebos were identical in appearance, taste and administration schedule; adherence	Low (Of the 74 children screened, 66 were randomized – 33 per group – and all randomized participants completed the 8–week follow–up with outcome data available for strengths and difficulties questionnaire scores and biomarkers; there were no	Low (Mental health outcomes were assessed using the validated strengths and difficulties questionnaire completed by parents, and biochemical endpoints, including serum 25–hydroxyvita min D3 [25(OH)D3] and magnesium, were measured with standard laboratory methods;	Some concerns (The clinical trial was registered with the Iranian Registry of Clinical Trials, and the published papers report the main behavioural and mental health outcomes – Conners' Parent Rating Scale and strengths and difficulties questionnaire – and key biochemical markers as planned; however, mental	Some concerns (Overall, the randomized, double–blind, placebo–controlled design, robust allocation concealment, high adherence, complete follow–up and validated outcome measures support a low risk of bias in most domains, but the dissemination of outcomes across multiple publications introduces some concerns about	Moderate (Evidence that vitamin D and magnesium co–supplementation improves total difficulties scores on the strengths and difficulties questionnaire in children with attention–deficit/hypera ctivity disorder comes from a single, well–conducted randomized controlled trial with an odds ratio of approximately 3.30 – 95% confidence interval 1.34–8.11 – favouring supplementation; certainty is downgraded

		baseline characteristics (age, anthropometry, methylphenidate dose and baseline biomarkers) were well balanced between groups, reducing concerns about selection bias)	exceeded 95% based on capsule counts and biomarker changes, and no systematic deviations from intended interventions related to prognosis or knowledge of assignment were reported)	losses to follow−up or differential attrition, making missing data an unlikely source of bias)	blinding of participants and assessors minimized differential measurement error, and the same instruments and procedures were applied across intervention and placebo groups)	health and behavioural outcomes were disseminated in more than one manuscript, and selective emphasis of particular SDQ domains versus others cannot be fully excluded from the available reports, leading to some concerns regarding the selection of the reported result)	selective reporting of specific strengths and difficulties questionnaire components; therefore, the overall risk of bias is judged as having some concerns)	one level for imprecision due to the modest sample size and wide confidence interval and one level for some concerns about selective outcome reporting, while other domains − randomization, adherence, outcome measurement and completeness of follow−up − are robust)
**8**	Hendren et al., (2016) [61]	Low (Children with autism spectrum disorder were randomized in equal proportions to methylcobalamin or saline placebo using a computer−generated allocation sequence held and implemented by an independent compounding pharmacy; sequential assignment to the next position on the	Low (The methylcobalamin and saline placebo injections were identical in volume and were masked by opaque tape around the syringes; families, outcome assessors and study staff were blinded to treatment	Some concerns (Of 57 randomized children, 50 − 27 methyl B_{12}; 23 placebo − contributed post−baseline Clinical Global Impressions−I mprovement data; three placebo participants were lost to	Low (The primary outcome, Clinical Global Impressions−Im provement was rated by a single expert child and adolescent psychiatrist blinded to treatment allocation, based on direct observation and	Low (The trial was prospectively registered with prespecified primary and secondary outcomes; the published report presents analyses for the primary Clinical Global Impressions−Impr ovement endpoint,	Some concerns (Overall risk of bias is judged as having some concerns, driven primarily by incomplete outcome data for a small subset of randomized participants and limited inclusion of fasting laboratory measures, while the randomization process, blinding,	Moderate (Evidence that subcutaneous methylcobalamin improves clinician−rated global symptoms of autism spectrum disorder is derived from a single, well−conducted randomized, double−blind, placebo−controlled trial with an odds ratio of approximately 4.77 (95% confidence interval

	randomization list and concealment of the list from investigators and families, together with balanced baseline characteristics – age, sex, intelligence quotient and baseline Aberrant Behavior Checklist and Social Responsiveness Scale scores – across groups, support an overall low risk of bias from the randomization process)	allocation; other behavioural and medical treatments were required to remain stable during the 8–week trial, and reported adherence to injections was high with no evidence of systematic deviations related to prognosis or knowledge of assignment, indicating a low risk of bias from deviations from intended interventions)	follow–up and four participants – one methyl B_{12}; three placebo – were excluded from all analyses due to incomplete Health Insurance Portability and Accountability Act documentation. Although attrition was modest and balanced with respect to group and no clear association with outcome was reported, the exclusion of these participants and incomplete outcome data for a subset of laboratory	structured discussion with parents; secondary outcomes, Aberrant Behavior Checklist and Social Responsiveness Scale, were validated parent–report instruments; laboratory biomarkers were measured using established high–performance liquid chromatography and electrochemical detection protocols in a specialized reference laboratory, and the same procedures were applied across groups,	Aberrant Behavior Checklist, Social Responsiveness Scale and key transmethylation/ transsulfuration biomarkers at 8 weeks using appropriate statistical tests; there is no evidence of selective reporting of favourable outcomes or unexplained omission of prespecified endpoints, suggesting low risk of bias from selection of the reported result)	adherence, outcome measurement and prespecification and reporting of outcomes are otherwise robust and at low risk of bias)	1.68–13.51) for better Clinical Global Impressions–Improvement scores compared with placebo; certainty is downgraded for imprecision due to the modest sample size and relatively wide confidence interval and for some concerns about missing outcome data, whereas strengths include rigorous randomization and blinding, validated outcomes and mechanistic biomarker correlations supporting biological plausibility)

				measures introduce some uncertainty, leading to some concerns regarding bias due to missing outcome data)	indicating low risk of bias in outcome measurement)			
**9**	Kaviani et al., (2022) [63]	Some concerns (Participants with mild to moderate depression were randomly assigned in equal numbers to vitamin D_3 or placebo following “simple randomization procedures according to entrance code” executed by the head of the project. Although this description implies a random allocation sequence, details on the exact method for sequence generation – for example, computer−generated random numbers – and on allocation concealment – for	Low (Vitamin D_3 and placebo soft−gel capsules were identical in appearance and packaging and were labelled with entrance codes, ensuring that participants, clinicians and investigators remained blinded to group allocation; only the project head responsible for randomization and labelling knew the assignment. Participants in both groups were	Low (Of 69 initially enrolled participants, 56 – 28 per group – met inclusion criteria and were randomized; all 56 completed the 8−week intervention and were included in the analysis according to the stated intention−to−treat approach. There were no reported dropouts,	Low (Depression severity was measured using the Beck Depression Inventory−II, a validated self−report instrument with established psychometric properties and a culturally adapted Persian version; assessments were performed at baseline and 8 weeks under standardized procedures. Laboratory biomarkers	Low (The trial had predefined primary and secondary outcomes (serum 25−hydroxyvitamin D [25(OH)D] concentration as primary; depression severity and inflammatory biomarkers as secondary), and the published report presents analyses and results for all prespecified outcomes at baseline and 8 weeks using appropriate	Some concerns (Overall risk of bias is judged as having some concerns, mainly due to incomplete reporting of the randomization sequence generation and allocation concealment procedures, despite balanced baseline characteristics. Other domains, including adherence to the assigned treatments, completeness and blinding of outcome assessments and	Moderate (The certainty of evidence that intermittent high−dose vitamin D_3 supplementation improves depression severity in adults with mild to moderate depression is rated as moderate. The rating reflects evidence from a single, relatively small, double−blind randomized clinical trial with an odds ratio of approximately 5.40 (95% confidence interval 2.01−14.52) for greater improvement in Beck Depression Inventory−II scores with vitamin D compared with placebo. Certainty is

	example, use of sealed, opaque envelopes or centralized randomization − are limited. Baseline characteristics − for example, age, sex, anthropometric indices, blood pressure and vitamin D status − were similar between groups, which supports successful randomization, but the lack of detailed reporting on sequence generation and concealment leads to some concerns about potential predictability or selection bias in the randomization process)	instructed to maintain their usual diet, physical activity level and medications, and adherence was actively monitored through pill counts, reminder calls and return of unused capsules. There were no reports of protocol deviations related to knowledge of the assigned intervention or differential co−interventions between groups, indicating low risk of bias due to deviations from intended interventions)	serious adverse events or differential loss to follow−up, and all randomized participants contributed outcome data for Beck Depression Inventory−II scores and inflammatory biomarkers at both time points, resulting in low risk of bias due to missing outcome data)	(25−hydroxyvitamin D [25(OH)D], intact parathyroid hormone (iPTH), interleukin−1β, interleukin−6 and high−sensitivity C−reactive protein were measured using established enzyme immunoassay kits in a research laboratory with quality control procedures, and the same methods were applied to both groups. Outcome assessors and laboratory personnel were unaware of group allocation. These features support	statistical tests. There is no evidence of selective reporting of favourable outcomes or omission of measured endpoints, and the statistical methods − for example, paired and independent tests with intention−to−treat analysis − match the described protocol. Therefore, the risk of bias from selection of the reported result is judged to be low)	reporting of outcomes, are at low risk of bias)	downgraded for some concerns regarding the randomization process and for imprecision related to the modest sample size and wide confidence interval, while being supported by rigorous blinding, complete follow−up, validated outcome measures and biologically plausible mechanisms consistent with vitamin D's neuroendocrine and anti−inflammatory roles)

					a low risk of bias in outcome measurement)			
**10**	Kerley et al., (2017) [64]	Low (Children with autism spectrum disorder were randomized to vitamin D_3 or placebo using an online computer−generated allocation program implemented by a physician who was not involved in recruitment or outcome assessment, while a separate nutrition researcher enrolled participants and dispensed coded bottles. Randomization was described as age− and gender−balanced, and baseline characteristics (age, sex distribution, body mass index, baseline Aberrant Behaviour Checklist scores and serum 25−hydroxyvitamin D [25(OH)D]) were similar between	Low (The trial was double−blind with identical−appearing vitamin D_3 and placebo drops dispensed in indistinguishable bottles; neither participants, parents, clinicians nor investigators knew allocation. Families were asked not to change diet, supplement use or therapies during the trial, and vitamin D−related behaviours were monitored with the VIDSun questionnaire. Compliance with study supplements exceeded 95% in	Some concerns (Of 42 randomized children, four – all allocated to vitamin D_3 – withdrew due to inconvenience of scheduled visits, leaving 38 completers – 18 vitamin D_3; 20 placebo – for analysis. Outcomes were analysed only in completers with no explicit intention−to−treat or sensitivity analysis including all randomized participants, and the differential	Low (Behavioural outcomes were measured using validated instruments widely used in autism spectrum disorder research: the Aberrant Behaviour Checklist, Social Responsiveness Scale and Developmental Disabilities−Children's Global Assessment Scale. Parent ratings and clinician assessments were conducted under standardized procedures at baseline and after 20 weeks,	Low (The primary endpoint (stereotypical behaviour subscale of the Aberrant Behaviour Checklist and secondary endpoints – additional ABC subscales, Social Responsiveness Scale, Developmental Disabilities−Children's Global Assessment Scale domains and biochemical markers – were prespecified and clearly reported. The published article presents results for all planned behavioural and biochemical	Some concerns (Overall risk of bias is judged as having some concerns, driven mainly by incomplete outcome data due to differential attrition in the vitamin D3 arm and the lack of explicit intention−to−treat analyses. The randomization process, blinding, adherence to interventions, outcome measurement and reporting appear robust and at low risk of bias)	Moderate (Certainty of evidence that daily oral vitamin D_3 supplementation at 2,000 international units for 20 weeks does not meaningfully improve core autistic behaviours as measured by the stereotypical behaviour subscale of the Aberrant Behaviour Checklist in children with autism spectrum disorder is rated moderate. The estimate is based on a single, relatively small randomized, double−blind, placebo−controlled trial with an odds ratio for greater improvement with vitamin D_3 versus placebo of approximately 0.49 (95% confidence interval 0.15−1.58) derived from standardized mean differences; certainty is downgraded for some

		groups, supporting an adequately randomized and concealed allocation process, even though specific details of sequence concealment − for example, sealed opaque envelopes − were not fully described)	both groups based on diaries and bottle checks, and there were no reported protocol deviations or differential co−interventions related to knowledge of treatment assignment, indicating low risk of bias from deviations from intended interventions)	attrition across groups − 0% placebo vs 18% vitamin D_3 − may have introduced some bias if dropouts differed systematically in prognosis or response. Although the reasons given suggest non−outcome−related withdrawal and baseline characteristics were similar, the absence of explicit analyses addressing missing data leads to some concerns in this domain)	and outcome assessors were blinded to treatment allocation. Biochemical markers, including serum 25−hydroxyvitamin D [25(OH)D] and C−reactive protein, were analysed in a local laboratory using established assays. There is no indication that measurement methods or thresholds differed between groups, supporting a low risk of bias in outcome measurement)	outcomes at baseline and follow−up using appropriate paired and independent statistical tests, with no evidence of selective outcome reporting or data−driven analytic choices that would favour particular results)		concerns about missing outcome data and for imprecision due to limited sample size and wide confidence intervals, but supported by otherwise rigorous trial conduct, validated outcome measures and a clear null behavioural signal despite substantial increases in serum 25−hydroxyvitamin D [25(OH)D])
**11**	Krivoy et al., (2017) [65]	Some concerns (Eligible schizophrenia patients maintained on clozapine were	Low (The trial was double−blind: vitamin D_3 and	Low (Of 47 randomized participants, 4	Low (Psychotic symptoms were measured using	Low (The registered protocol and methods section	Some concerns (Overall, the risk of bias is judged as having some	Moderate (Certainty of evidence that weekly vitamin D_3 supplementation at

	randomly assigned 1:1 to vitamin D_3 or placebo using a pre−prepared randomization code generated and held by the study pharmacist, who was the only team member with access to the allocation list; investigational bottles were sealed, opaque and identically labelled with study codes, and baseline demographic, clinical and metabolic characteristics were similar between groups, suggesting adequate sequence generation and allocation concealment. However, the method used to generate the random sequence − for example, computer−generated random numbers− and specific concealment procedures − for example, block size,	placebo oral drops were indistinguishable in appearance and packaging, and bottles were dispensed according to randomization codes without revealing allocation to participants, clinicians, investigators or outcome assessors; weekly dosing was administered directly by a study nurse, ensuring high adherence and minimizing protocol deviations. Participants were asked to maintain their existing clozapine regimen, concomitant medications and	− 2 per group − did not complete all follow−up visits, but all 47 were included in the primary analyses under an intention−to−treat framework using last observation carried forward for missing week−8 values. The small, balanced attrition, clearly documented reasons for loss to follow−up and inclusion of all randomized patients in outcome analyses support a low risk of bias	the Positive and Negative Syndrome Scale, depressive symptoms with the Calgary Depression Scale, cognition with the Montreal Cognitive Assessment and metabolic parameters with standard anthropometric and laboratory measures; all instruments are validated, were applied according to published procedures at baseline and week 8 and were administered by trained raters or laboratory staff blinded to treatment allocation. There	pre−specified PANSS total as the primary outcome and PANSS subscales, Calgary Depression Scale, Montreal Cognitive Assessment and metabolic parameters as secondary outcomes; the published report presents results for all these endpoints at the planned time points using appropriate repeated−measures analyses without evidence of selective omission or emphasis on particular outcomes. Although exploratory cognitive and subgroup	concerns, primarily due to incomplete reporting of the randomization sequence generation and allocation concealment, despite otherwise well−conducted blinding, adherence monitoring and outcome assessment, and balanced attrition with intention−to−treat analyses)	14,000 international units for eight weeks does not produce a clinically important improvement in global psychotic symptoms − Positive and Negative Syndrome Scale − total) in chronic clozapine−treated schizophrenia patients is rated moderate. The rating reflects evidence from a single, well−conducted randomized, double−blind, placebo−controlled clinical trial with low risk of bias in most domains but some concerns about the randomization process and imprecision due to the modest sample size and wide confidence intervals around the odds ratio − OR ≈ 0.80; 95% confidence interval ≈ 0.28–2.26. The direction and magnitude of effect, lack of benefit across PANSS

		stratification − were not fully described, leading to some concerns about the transparency of the randomization process despite balanced baseline characteristics)	lifestyle factors, and there were no reports of differential co−interventions or contamination between arms, indicating low risk of bias due to deviations from intended interventions)	from missing outcome data, although last observation carried forward is a conservative method that assumes stability after dropout)	is no indication that outcome assessment differed between groups or was influenced by knowledge of intervention status, supporting a low risk of bias in measurement of outcomes)	analyses were conducted, they are clearly labelled as post hoc and do not appear to drive selective reporting of favourable results, leading to a low risk of bias from selection of the reported result)		subscales and depressive symptoms and consistent null findings for metabolic parameters support downgrading for imprecision but not for inconsistency or indirectness in this population of treatment−resistant schizophrenia patients)
**12**	Libuda et al., (2020) [67]	Low<br>(Randomization was performed by an independent Institute of Medical Biometry using computer−generated random number lists with variable block sizes and stratification by Beck Depression Inventory−II severity and 25−hydroxyvitamin D [25(OH)D] baseline strata. Allocation concealment was maintained via pre−labelled identical	Low<br>(The trial was double−blind with participants, parents, clinicians and outcome assessors masked to allocation; both groups received the same treatment as usual − treatment as usual, TAU − and identical−appearing capsules. There is no evidence of systematic	Some concerns<br>(Of 113 randomized participants, 13 − approximately 11.5% − were lost to follow−up, resulting in 100 participants in the modified intention−to−treat analysis. While the primary analysis appropriately	Low<br>(Depressive symptoms were assessed using validated instruments with good internal consistency in adolescents, including Beck Depression Inventory−II and the Diagnostic System for Mental Disorders in Childhood and	Low<br>(The primary outcome − change in Beck Depression Inventory−II− and secondary outcomes − serum 25−hydroxyvitamin D [25(OH)D], parent and self−rated Diagnostic System for Mental Disorders in Childhood and Adolescence depression scale scores − were	Some concerns<br>(Overall risk of bias is judged as some concerns, primarily due to incomplete follow−up and the reliance on a modified intention−to−treat dataset despite appropriate imputation sensitivity analyses. All other domains are low risk, but the moderate level of attrition and limited detail on reasons for dropout	Low<br>(Under the GRADE framework, certainty of evidence for the effect of short−term vitamin D_3 supplementation on self−rated depressive symptoms − Beck Depression Inventory− in this population is rated low. Randomized controlled trials start at high certainty but are downgraded one level for imprecision, as the OR ≈ 0.83; 95% confidence interval 0.41−1.70, encompasses both potentially

	pill dispensers prepared by the manufacturer, and baseline characteristics were well balanced across groups, supporting an adequate randomization process)	deviations from intended interventions related to knowledge of the assigned intervention, and adherence was monitored through clinical follow–up during the 28–day period, supporting a low risk of bias for deviations from intended interventions)	included participants within their randomized group and a sensitivity analysis imputed missing Beck Depression Inventory–II values via linear regression using baseline predictors, attrition was slightly unbalanced – 9 versus 4 and reasons for loss to follow–up are not exhaustively detailed, leaving some risk that missingness may be related to outcomes)	Adolescence depression scale, DISYPS–DES, administered under standardized conditions at baseline and day 28. Outcome assessors and participants were blinded to allocation, and there is no indication of differential measurement or inappropriate outcome tools, supporting a low risk of bias in outcome measurement)	prespecified in the protocol and trial registration, and results are reported comprehensively, including sensitivity and exploratory analyses. There is no evidence of selective reporting or outcome switching, so the risk of bias from selective reporting is judged low)	preclude a global low risk judgment)	meaningful benefit and harm, and one level for indirectness related to the short intervention duration – 28 days– adjunctive use with treatment as usual only, and restriction to child and adolescent in–/day–patients in a single country, limiting generalizability and precluding conclusions regarding longer–term serotonergic and dopaminergic modulation. No upgrading is applied because there is no large effect signal or consistent dose–response pattern)

**13**	Marsh et al., (2017) [69]	Low (Participants were randomized 1:1 to vitamin D_3 or placebo using blocked allocation implemented by the institutional investigational drug service, with allocation concealed from investigators, coordinators and participants. Baseline demographic and clinical characteristics, including Montgomery–Åsberg Depression Rating Scale, Young Mania Rating Scale, Hamilton Anxiety Rating Scale and serum 25–hydroxyvitamin D [25(OH)D], were similar between groups, supporting an adequate randomization process)	Low (The trial was double–blind, with vitamin D_3 and placebo capsules identical in appearance and packaging; participants, clinicians, assessors and analysts were masked to treatment assignment until data lock. Psychotropic regimens were required to be stable prior to entry and remained largely unchanged, with only one recorded medication adjustment for depression during follow–up; there is no evidence that knowledge of intervention influenced care or adherence.	Some concerns (Of 33 randomized participants, 8, approximately 24%, withdrew after randomization – 5 in the vitamin D_3 group and 3 in the placebo group– before the first post–baseline assessment, leading to 25 completers contributing to endpoint analyses. Although reasons for withdrawal are not clearly detailed and attrition is moderately high, dropouts occurred early and there is no strong indication that	Low (Depressive, anxiety and mood elevation symptoms were measured with validated clinician–rated instruments administered at baseline and every two weeks by trained raters who were blinded to treatment allocation. There is no suggestion of differential measurement, instrument instability or outcome assessment influenced by knowledge of group assignment)	Low (Primary – change in Montgomery–Åsberg Depression Rating Scale, MADRS– and secondary, Young Mania Rating Scale, YMRS; Hamilton Anxiety Rating Scale, HAM–A; serum 25–hydroxyvitamin D [25(OH)D]; adverse events outcomes were pre–specified and are reported in the manuscript, with figures and tables consistent with the stated aims. No selective reporting, unacknowledged outcome switching or unexplained omission of key outcomes is apparent)	Some concerns (Taking all domains together, the trial is judged to have some concerns overall, driven mainly by the moderate, slightly imbalanced attrition before the first follow–up and the reliance on completer data rather than a fully conservative intention–to–treat framework. Randomization, blinding, outcome measurement and reporting are otherwise robust)	Low (Under the GRADE framework, the certainty of evidence for the effect of adjunctive vitamin D_3 on depressive symptoms in bipolar depression is rated low. Randomized controlled trials begin at high certainty but are downgraded one level for risk of bias – attrition and use of completer analyses– and one level for serious imprecision, as the OR ≈ 0.53; 95% confidence interval 0.12–2.21, is extremely wide and comfortably includes both clinically relevant benefit and harm. Indirectness and inconsistency are not major concerns, but the small single–centre sample and short duration – 12 weeks– further limit generalizability and confidence in the magnitude of effect)

			Deviations from intended interventions, if present, are unlikely to be related to the true outcome and appear balanced across groups)	missingness differed by outcome; however, the absence of a strict intention−to−treat analysis and limited information on the missing data mechanism justify a judgment of some concerns)				
**14**	Mazahery et al. (2019) [70]	Some concerns (Randomisation was stratified by age and autism spectrum disorder severity with four parallel arms − VID, OM, VIDOM, placebo − and baseline characteristics were generally well balanced, but the main report refers to a separate published protocol for full details of sequence generation and allocation	Low (The trial was described as double−blind with identical capsules and dispensers; participants, caregivers, investigators, and outcome assessors were masked, and compliance was high across groups. Reported deviations from	High (Of 117 randomised children, only 73, ≈62%, completed the 12−month follow−up for primary behavioural outcomes, with differential non−completion by ethnicity − higher	Low (Core symptoms were assessed with validated caregiver−reported instruments −Social Responsiveness Scale and Sensory Processing Measure − administered using standardised procedures;	Some concerns (Multiple behavioural and sensory outcomes and subdomains were analysed with emphasis on nominal P−values and trends, P < 0.1, without adjustment for multiplicity. Although main tables appear comprehensive and there is no	High (Overall risk of bias is judged high, driven primarily by substantial attrition with imbalanced non−completion and complete−case analyses, together with some concerns about the randomisation reporting and selective emphasis on nominally significant or	Low (As a single, relatively small RCT with high attrition, sparse events for "positive response" in SRS−total, and very wide confidence intervals around the odds ratio for clinically meaningful improvement, the certainty of evidence for an effect of vitamin D_3 and/or omega−3 LCPUFA on core autism spectrum disorder

		concealment, leaving minor uncertainty about the randomisation process).	intended interventions –e.g., dislike of supplements, time constraints – were symmetric and primarily related to burden rather than knowledge of allocation)	drop–out in Pacific children – and autism spectrum disorder severity. Analyses were performed on completers only without robust handling of missing data, and attrition is plausibly related to prognosis and treatment tolerability, introducing a serious risk of bias)	outcome assessors – parents and clinicians – were blinded to group allocation, and the same tools were applied consistently across arms and timepoints)	clear evidence of selective omission of unfavourable outcomes, the flexible analytic approach and numerous comparisons raise some concern for selective emphasis)	trend–level findings across many correlated endpoints)	symptoms is rated low due to serious risk of bias and serious imprecision)
**15**	Misal et al., (2024) [71]	Some concerns (Allocation to case – vitamin B_{12} plus antidepressant – or control – antidepressant alone – was carried out using a computer–generated random table, but the article does not specify	High (The design was explicitly open–label with no placebo injections; participants, treating clinicians and raters were aware of whether	Some concerns (The CONSORT flow diagram indicates some loss to follow–up, but primary analyses for Hamilton	Some concerns (Depression severity was assessed with validated clinician–rated – Hamilton Rating Scale for Depression, HAM–D – and	Some concerns (Response, ≥ 50% reduction in HAM–D, and remission, HAM–D ≤ 7, were defined a priori, consistent with standard major depressive	High (Considering the open–label nature of the trial, absence of placebo control, potential baseline imbalances, and incomplete protection against bias from	Low (Under the GRADE framework, this randomized controlled trial starts at high certainty but is downgraded one level for serious risk of bias – open–label design, lack of placebo, some

	details of allocation concealment procedures − for example, sealed envelopes or central allocation. Baseline demographic and clinical variables were mostly comparable except for longer total duration of illness and a higher number of past episodes in the case group, raising some concern that randomization may not have fully balanced prognostic factors)	vitamin B_{12} injections were administered. Co−interventions, expectations and adherence could therefore have been influenced by knowledge of treatment, and these deviations are plausibly related to outcomes, particularly given the subjective nature of depression ratings in an elderly population. There is no indication that analyses appropriately adjusted for such deviations, leading to a high risk of bias in this domain)	Rating Scale for Depression and Geriatric Depression Scale used an intention−to−treat framework with all 62 participants included in repeated−measures models. Nevertheless, the handling of missing item−level or visit−level data is not fully described, and denominators for binary response and remission outcomes assume complete data − 32 and 30 − which may mask informative missingness. Because it is unclear	self−report − Geriatric Depression Scale, GDS−H Hindi − instruments at three time points, but the absence of blinding may have influenced ratings, especially for clinician−administered HAM−D in an open−label context. There is no evidence that raters were separate from treating clinicians or shielded from knowledge of group assignment, so measurement of the outcome may have been affected by awareness of intervention)	disorder trial conventions, but the article also reports an additional remission cut−off, HAM−D ≤ 11, post hoc to align with geriatric literature, with emphasis on number needed to treat. Although main continuous and binary outcomes are presented transparently, the introduction of alternative thresholds and multiple time points − days 14 and 28 − without adjustment for multiplicity introduces some concern about selective emphasis)	deviations and outcome measurement, the overall risk of bias is judged high, despite the use of randomization and standardized instruments)	baseline imbalance and possible outcome−assessment bias − and one level for serious imprecision (small sample size; wide confidence interval for remission OR ≈ 2.25; 95% confidence interval 0.75–6.76. Indirectness is limited because the population and setting are clinically relevant for late−life depression, LLD, but the very short follow−up − 4 weeks − further constrains generalizability. No upgrading factors are present; therefore, overall certainty is rated low)

				whether missing data are completely at random, some concerns are warranted)				
**16**	Mohammadpour et al., (2018) [72]	Some concerns (Participants were stratified by gender and randomized to vitamin D_3 or placebo using permuted−block randomization; tablets were identical in appearance. Baseline demographic and clinical characteristics were similar between groups. However, specific details on allocation concealment mechanisms − for example, central randomization or use of opaque sealed envelopes − were not provided, leaving some residual uncertainty about the robustness of the randomization process)	Low (The trial was explicitly double−blind: vitamin D_3 and placebo tablets were visually identical, and participants, parents, clinicians and outcome assessors were masked to group allocation. All children received methylphenidate titrated according to a standardized protocol, and there is no evidence of systematic deviations from intended interventions that were related to knowledge of	Some concerns (Of 62 randomized participants, 54 completed the 8−week intervention, 13% attrition, and for some scales − for example, Weekly Parent Ratings of Evening and Morning Behavior, WPREMB − the effective sample sizes at week 8 were 25 versus 29, indicating additional item− or visit−level missingness. Reasons for	Low ADHD symptoms and behavioural outcomes were assessed using validated instruments − Conners' Parent Rating Scale−Revised [S], ADHD Rating Scale−IV, Weekly Parent Ratings of Evening and Morning Behavior − administered in a standardized fashion at three time points, with raters blinded to treatment assignment. The constructs	Low (The primary and secondary outcomes − Conners' Parent Rating Scale−Revised [S], ADHD Rating Scale−IV, Weekly Parent Ratings of Evening and Morning Behavior, serum 25−hydroxyvitamin D [25(OH)D] − were pre−specified and fully reported across time points, with corresponding tables and P−values. Although greater emphasis is placed in the discussion on the	Some concerns (Overall risk of bias is judged as some concerns, mainly due to incomplete reporting of allocation concealment and the use of completer−based analyses in the presence of modest attrition and questionnaire−level missing data. Randomization, blinding, and outcome measurement are otherwise sound, and selective reporting does not appear to be a major issue)	Moderate (Under the GRADE framework, this randomized controlled trial starts at high certainty. It is downgraded one level for imprecision because of the relatively small sample size and the wide confidence interval around the OR ≈ 3.53; 95% confidence interval 1.30–9.58 − for reduction in evening symptoms, which, while excluding no effect, still spans a broad range of plausible effect sizes. The risk of bias is rated as some concerns but not high, and indirectness and inconsistency are limited because the population and setting are directly relevant to the target ADHD

			allocation. Compliance was high and monitored by pill counts and biochemically via serum 25−hydroxyvitamin D [25(OH)D] levels)	dropout and missing questionnaires are not fully detailed, and analyses of symptom trajectories were conducted on completers rather than under a strict intention−to−treat framework with appropriate imputation, so the risk that missing data are related to both intervention and outcome cannot be excluded)	measured are directly relevant to ADHD severity and daily functioning, and there is no indication of differential measurement errors between groups)	statistically significant group differences in evening and total WPREMB scores, there is no evidence of non−reporting of unfavourable outcomes or post hoc outcome switching)		subgroup. The resulting certainty of evidence for vitamin D_3 augmentation improving evening behavioural symptoms in methylphenidate−treated children with ADHD is therefore moderate)
**17**	Naeini et al., (2019) [73]	Some concerns (The article describes a randomized, double−blind, placebo−controlled design with parallel groups of similar size	Low (Vitamin D_3 and placebo tablets were identical in appearance and given once daily after lunch;	Some concerns (Of 84 initially recruited students, 71 − 36 in the vitamin D_3 group, 35 in	Low (Psychiatric and behavioural outcomes were assessed with validated, translated	Low (The primary tools − Conners Parent Questionnaire, CPQ; Strengths and Difficulties	Some concerns (The main limitations are incomplete reporting of randomization and allocation	Moderate (Under the GRADE framework, this randomized controlled trial starts at high certainty. It is downgraded one level

	and comparable baseline characteristics for age, sex, body mass index, BMI, physical activity, dietary vitamin D intake and most psychiatric scores. However, the method of sequence generation and allocation concealment – for example, central randomization, sealed opaque envelopes – is not fully detailed, and SDQP baseline scores differed significantly between groups, suggesting that randomization may not have perfectly balanced all prognostic factors)	parents, children, teachers and investigators were masked with respect to allocation. All participants were maintained on methylphenidate with similar dosing schedules, and there is no evidence of systematic deviations from the intended interventions related to knowledge of assignment. Tablet adherence was monitored, and both arms were followed with the same schedule and co−intervention restrictions)	the placebo group – completed the study; attrition was 6 of 42 in the intervention arm – poor compliance or withdrawal for personal reasons – and 7 of 42 in controls – needle phobia or refusal of the second blood draw. Analyses were based on the 71 completers using paired and independent t−tests and analysis of covariance −analysis of covariance, ANCOVA− , without a clearly described	instruments −Conners Parent Questionnaire, CPQ; Strengths and Difficulties Questionnaire Parent Version, SDQP; Strengths and Difficulties Questionnaire Teacher Version, SDQT; Continuous Performance Test, CPT – implemented with standardized procedures. Outcome raters −parents, teachers, computer−based CPT− were not informed of group allocation, and the constructs measured directly reflect ADHD symptom burden and	Questionnaire, SDQ; Continuous Performance Test, CPT; serum 25−hydroxyvitamin D [25(OH)D] – were prespecified and reported with pre− and post−intervention values and P−values. Subscale analyses for SDQ and CPT are reported in detail, and there is no evidence of selective omission of unfavourable outcomes, although the focus in the discussion is understandably placed on domains that showed statistically significant effects)	concealment and the use of completer−based analyses in the presence of moderate attrition, which could bias effect estimates if dropouts differed systematically in symptom trajectory. Blinding, measurement, and outcome reporting are otherwise appropriate. Overall, the risk of bias is judged as some concerns rather than low or high)	for some concerns regarding risk of bias – limited information on allocation concealment, baseline imbalance in SDQP, completer analyses without robust handling of missing data – and one level for imprecision due to relatively small sample size and wide confidence intervals around the OR ≈ 3.85; 95% confidence interval 1.62–9.13. Indirectness and inconsistency are minimal because the population – school−aged children with attention deficit hyperactivity disorder, ADHD on methylphenidate – and outcomes – parent−rated symptoms – are directly relevant to the target subgroup, and no serious publication bias is suspected. Overall certainty of evidence for vitamin D_3 improving parent−rated ADHD

				intention−to−treat approach or imputation for missing questionnaire data, so missingness may be related to both adherence and outcomes)	functioning. There is no indication of differential measurement error between vitamin D_3 and placebo groups)			symptoms in this context is rated moderate)
**18**	Okereke et al., (2020) [75]	Low (Randomization was computer−generated within sex, race, and 5−year age strata in block sizes of eight as part of the Vitamin D and Omega−3 Trial, VITAL, infrastructure, with central allocation and no evidence of compromised concealment. Baseline characteristics − age, sex, race/ethnicity, body mass index, comorbidity burden, baseline serum 25−hydroxyvitamin D [25(OH)D], supplemental vitamin D use, physical	Low (The trial maintained double blinding of participants, clinicians, and outcome assessors with identical−appearing vitamin D_3 and placebo capsules and factorial co−randomization with fish oil. Adherence to study medication was high, ≥ 90% taking at least two−thirds of study pills in both arms throughout follow−up. There	Low (Overall completion rate was 90.5% for follow−up − 93.5% among those alive at the end of the trial − with similar retention across treatment arms. Longitudinal mood outcomes were analysed using repeated−measures models − general linear models of	Low (Depression events were defined using validated criteria, including clinician−diagnosed depression, initiation of depression treatment, or clinically relevant depressive symptoms based on 8−item Patient Health Questionnaire depression scale scores ≥ 10 points, obtained	Low (The trial was prospectively registered and conducted according to a pre−specified protocol and statistical analysis plan. Primary and secondary outcomes − composite depression events, incident and recurrent depression, and change in 8−item Patient Health Questionnaire depression scale scores − were	Low (All five domains under the Cochrane Risk of Bias 2 tool were judged to be at low risk of bias, based on robust randomization procedures, strong adherence and blinding, minimal and balanced missing data, valid and consistent outcome ascertainment, and transparent, protocol−driven reporting. Overall, the risk of bias for the primary depression	Moderate (Under the GRADE framework, randomized controlled trials start at high certainty. For this question, certainty is downgraded one level to moderate due to indirectness: participants were generally vitamin D−replete older adults without current depressive symptoms, so results pertain to universal prevention in largely sufficient populations and may not fully generalize to individuals with frank vitamin D deficiency, active depressive

	activity, and geographic region – were well balanced between the vitamin D_3 and placebo groups, suggesting successful randomization without significant prognostic imbalances)	is no indication of systematic deviations from intended interventions related to knowledge of assignment, and the authors performed sensitivity analyses censoring at nonadherence that did not materially change results, supporting low risk of bias from deviations)	response profiles – that accommodate missing 8−item Patient Health Questionnaire depression scale observations under a missing−at−random assumption, and depression events were ascertained annually with clear definitions. There is no evidence that loss to follow−up or missing PHQ−8 data was differential by group in a way likely to substantially bias effect estimates for	via standardized annual questionnaires. The 8−item Patient Health Questionnaire depression scale is a widely validated screening instrument for current depression in population studies, and a subset of participants underwent in−person diagnostic interviews for validation. Outcome assessment was identical in both arms and masked to treatment allocation, and misclassification of depression status is likely	reported comprehensively with hazard ratios – hazard ratios, confidence intervals, and subgroup analyses. Any protocol modifications were transparently documented in supplementary materials, and there is no evidence of selective reporting of favourable results or suppression of null or adverse findings)	outcomes is considered low)	disorders, or specific psychiatric diagnoses of interest such as major depressive disorder in younger adults or treatment−resistant depression. Imprecision is limited for the primary composite outcome, OR ≈ 0.97; 95% confidence interval, 0.87−1.09, which effectively rules out moderate beneficial or harmful effects at the population level, and inconsistency and publication bias are unlikely within this large, well−conducted trial)

				depression outcomes)	nondifferential, leading at most to bias toward the null rather than spurious benefit or harm)			
**19**	Omidian et al., (2019) [76]	Some concerns (Randomization was stratified by sex and body mass index and implemented via random permuted blocks using computer−generated sequences, with allocation to "vitamin D" or "placebo" coded as A and B for investigators and participants. Baseline characteristics − age, sex distribution, BMI, blood pressure, fasting blood sugar, hemoglobin A1c, insulin, lipid profile, Beck Depression Inventory−II scores− were similar between groups, supporting successful randomization. However, details on	Low (The trial is described as double−blind for specialists, staff, and patients, with vitamin D_3 and placebo tablets identical in appearance −shape, size, colour − and packaging. Both groups continued their usual antidiabetic medications and were instructed to maintain stable diet and physical activity, and adherence to the trial tablets was monitored by weekly telephone calls and pill counts, with	Some concerns (Of 68 randomized participants, 64 −32 vitamin D, 34 placebo − completed the 12−week trial; two subjects in each group withdrew due to personal reasons. Analyses were conducted on completers using independent t−tests for changes in outcomes, without explicit intention−to−treat analyses or imputation for missing data.	Low (Depressive symptoms were assessed with the validated Beck Depression Inventory−II Persian version, administered before and after the intervention, and metabolic outcomes were measured with standardized laboratory methods. Outcome assessment procedures were applied identically in both arms, and the double−blind design means outcome	Low (The primary outcome − change in Beck Depression Inventory−II score − and secondary metabolic outcomes − hemoglobin A1c, insulin, triglycerides, fasting blood sugar, lipid profile, blood pressure, serum 25−hydroxyvitamin D [25(OH)D] − were clearly pre−specified and fully reported with baseline and post−intervention values and between−group comparisons. P−values are	Some concerns (Overall, the trial is reasonably well conducted with appropriate randomization, blinding, and outcome measurement, but lack of detailed allocation concealment procedures and reliance on completer analyses without formal intention−to−treat methods result in an overall judgment of some concerns rather than unequivocally low risk of bias)	Moderate (Under the GRADE framework, randomized controlled trials begin at high certainty. The certainty is downgraded one level for some concerns regarding risk of bias − incomplete reporting of allocation concealment, use of completer analyses − and one level for imprecision due to small sample size and a wide confidence interval around the OR ≈ 2.06; 95% confidence interval 0.85–4.99, which includes the possibility of no effect and a fairly broad range of plausible benefit. Indirectness and inconsistency are limited within this specific population of type 2 diabetes mellitus

		the specific allocation concealment mechanism – for example, central randomization, sealed opaque envelopes – are not fully described, so some uncertainty remains)	non–adherence defined a priori. There is no evidence that knowledge of allocation influenced co–interventions or behaviours, making deviations from intended interventions unlikely to introduce substantial bias)	Given the small and balanced attrition and lack of evidence that dropouts were related to both intervention and outcome, the risk of bias from missing outcome data is probably low to modest, but cannot be completely excluded)	assessors and participants did not know allocation. There is no indication of differential measurement error or systematic bias in outcome ascertainment)	presented for all key outcomes, and there is no evidence of selective omission of null or negative results or post hoc outcome switching)		patients with mild to moderate depressive symptoms and vitamin D deficiency, and there is no clear signal of publication bias. Overall certainty of evidence is therefore rated moderate for the effect of vitamin D_3 on depressive symptoms in this setting)
**20**	Penckofer et al., (2022) [77]	Low (Randomization was computer–generated by an independent statistician with stratified block randomization based on baseline Center for Epidemiologic Studies Depression severity; allocation was implemented by a research pharmacist preparing identically appearing capsules.	Low (The trial was double–blind; participants, investigators and outcome assessors were unaware of assignment to 50,000 or 5,000 international units weekly. Both groups received active vitamin D_3 capsules with	Low (Of 129 randomized women, 122, 95%, completed the three–month and 119, 92%, the six–month follow–up assessments. Attrition was low and similar across arms –	Low Depressive symptoms were measured with validated self–report instruments – Center for Epidemiologic Studies Depression; Patient Health Questionnaire–9 , with good internal	Low (The protocol pre–specified primary and secondary outcomes and analytic approaches; the published report presents CES–D and Patient Health Questionnaire–9 trajectories, 25–hydroxyvitami	Low (Across RoB 2 domains, risks of bias are consistently low: randomization and blinding were appropriate, attrition was low and balanced, and outcome measurement and reporting were rigorous. The main limitation relates to	Moderate (Under the GRADE framework, this randomized controlled trial starts as high–certainty evidence for the effect of vitamin D_3 dosing on depressive symptoms in women with type 2 diabetes and low 25–hydroxyvitamin D [25(OH)D]. One level is downgraded for imprecision, as the

	Baseline characteristics, including age, race, diabetes duration, vitamin D status, depression scores and cardiometabolic measures, were well balanced between groups, supporting adequate randomization and allocation concealment)	similar appearance and dosing schedule, reducing risk of performance bias. Adherence was monitored through follow−up visits and adverse event reporting; no systematic deviations from the intended interventions related to the trial context were reported)	primarily loss to follow−up or unrelated medical issues − and analyses used linear mixed−effects models approximating intention−to−treat by including all available observations. There is no evidence that missing outcome data were related to both intervention and outcome in a way that would substantially bias the effect estimate)	consistency − Cronbach's alpha ≥ 0.8 across visits−. Laboratory measurements, including 25−hydroxyvitamin D [25(OH)D], were performed in a Clinical Laboratory Improvement Amendments −certified laboratory using standardized assays. Outcome assessments were applied consistently at baseline, 3 and 6 months, and blinding of participants and investigators minimizes risk of differential outcome assessment)	n D [25(OH)D] changes and adverse events comprehensively. There is no indication of selective reporting of favourable time points or outcome metrics beyond exploratory stratified analyses that are clearly labelled as such)	feasibility−driven under−recruitment relative to the original power calculation, which affects precision rather than internal validity. Overall, the risk of bias is judged low)	sample size − N = 129 versus a planned 150 − and wide confidence intervals around the OR ≈ 0.77; lowerOR ≈ 0.37; upperOR ≈ 1.59, do not exclude either a modest benefit or harm of high−dose versus low−dose supplementation. An additional concern is some indirectness for our main contrast of interest − very high−dose versus physiological replacement vitamin D_3 rather than vitamin D_3 versus placebo or no supplementation. Taken together, the evidence is rated as moderate certainty for the comparative effect of high−dose versus low−dose vitamin D_3 on depressive symptom outcomes in this comorbid metabolic−psychiatric population)

**21**	Rahman et al. (2023) [79]	Low (Central computer−generated permuted block randomization stratified by age, sex, and state of residence ensured adequate allocation sequence and concealment; baseline sociodemographic and clinical characteristics were well balanced between vitamin D_3 and placebo groups, and there is no indication of inadequate randomization)	Low (Double−blind design with identical monthly gel capsules for vitamin D_3 and placebo; adherence was high – over 80% of participants reported taking at least 80% of dispensed tablets – off−trial vitamin D use was monitored, and there is no evidence of differential co−interventions or systematic deviations from intended assignments that would bias the comparison)	Low (Analyses of Patient Health Questionnaire −9 scores included 96% of randomized participants with at least one post−randomiz ation assessment, and analyses of incident antidepressant use excluded only participants without Pharmaceutica l Benefits Scheme linkage or prevalent antidepressant use; loss to follow−up and mortality were low relative to sample size and similar across arms,	Low (Depressive symptoms were measured with the validated Patient Health Questionnaire−9 at years 1, 2, and 5 using standardized self−administere d questionnaires; incident antidepressant use was captured objectively from Pharmaceutical Benefits Scheme dispensing records; outcome assessors and data analysts remained blinded to allocation until after primary trial analyses, minimizing measurement bias)	Low (The trial was prospectively registered with depression defined as a pre−specified tertiary outcome, and a detailed statistical analysis plan was finalized prior to unblinding; planned analyses used mixed models for repeated measures and flexible parametric survival models, and there is no evidence of selective reporting of favourable results or outcome switching for the depression endpoints)	Low (Across all domains, the randomized controlled trial exhibits robust methodology with adequate randomization, blinding, high adherence, minimal and balanced missing outcome data, and prespecified analytic strategies; the main limitation is that depression was a tertiary rather than primary outcome, but this does not materially increase bias risk given the large sample and stable estimates)	High (Large−scale randomized controlled trial with low risk−of−bias, long follow−up – up to five years – and precise effect estimates for clinically relevant depression – OR ≈ 0.99, with narrow 95% confidence interval, 0.90−1.08, directly applicable to community−dwelling older adults; downgraded neither for risk−of−bias nor for imprecision, providing high certainty that routine monthly high−dose vitamin D_3 supplementation does not confer a clinically important reduction in late−life depression in predominantly vitamin D−replete populations)

				making informative missingness unlikely for the depression outcomes)				
**22**	Rouhi et al. (2018) [80]	Low (The allocation sequence was generated by computer–based block randomization with a 1:1 ratio, and participants were assigned using opaque, sealed, consecutively numbered envelopes prepared by a person outside the study. Baseline sociodemographic and clinical characteristics, including age, education, and baseline Edinburgh Postnatal Depression Scale and Fatigue Identification Form scores, were similar between groups, supporting adequate	Low (The trial was double–blind: vitamin D_3 and placebo capsules were identical in appearance and supplied in coded form by a pharmaceutical company; participants and investigators were unaware of allocation. Participants were instructed not to use other vitamin D supplements and were contacted monthly by telephone to support adherence. There is no evidence of systematic	Low (Of 95 eligible women, 83 consented and 80 were randomized – 40 per arm. All 80 randomized participants completed the six–month follow–up assessments for Edinburgh Postnatal Depression Scale and Fatigue Identification Form scores, with no post–randomization losses. Missing outcome data are therefore negligible and	Some concerns (Depressive symptoms and fatigue were measured with validated self–report instruments – EPDS and FIF – but there is no explicit statement that outcome assessors were blinded to allocation when administering questionnaires, although participants themselves were blinded. Self–report scales are appropriate for postpartum mood and	Some concerns (The study does not reference a pre–registered protocol, and depression and fatigue are reported as primary outcomes, but the statistical analysis focuses predominantly on within–group changes, before–after, rather than pre–specified between–group comparisons. Only summary statistics – means, standard deviations, and p–values – are provided for Edinburgh	Some concerns (Domains related to randomization, deviations from intended interventions, and missing data are at low risk of bias, but there are some concerns regarding the exclusive use of self–report outcomes and lack of a pre–registered protocol or fully prespecified analysis plan. Taken together, the overall risk of bias is judged as some concerns rather than low)	Low (Under the GRADE framework, this randomized controlled trial starts as high–certainty evidence for the effect of vitamin D_3 on postpartum depressive symptoms. One level is downgraded for risk of bias because of reliance on self–report scales, absent registration, and incomplete detail on pre–specified analyses. A second level is downgraded for imprecision, as the trial is small, N = 80, and the OR, for depression improvement, ≈ 6.78, has a wide 95% confidence interval 2.90–15.86. In addition, indirectness is present because serum

		randomization and concealment)	deviations from the intended interventions related to the trial context)	unlikely to bias the estimated intervention effect)	fatigue screening, but they may be influenced by social desirability and unmeasured contextual factors − for example, social support, life stress. No clinician−rated diagnostic interview was used to confirm major depressive disorder. Overall, the risk related to outcome measurement is judged as some concerns due to exclusive reliance on self−report)	Postnatal Depression Scale and Fatigue Identification Form scores at baseline and follow−up; no confidence intervals for between−group differences or adjusted analyses are reported. This raises some concerns about selective emphasis on favourable results and incomplete reporting of all planned analyses)		25−hydroxyvitamin D [25(OH)D] levels were not measured, so true vitamin D status and change cannot be confirmed. Overall, the certainty of evidence for a causal antidepressant effect of vitamin D_3 in this specific postpartum population is rated as low)
**23**	Vaziri et al. (2016) [85]	Some concerns (Block randomization stratified by parity was used and described, but	Some concerns (The trial used a single−blind design: the midwife	Some concerns (Of 169 randomized participants, 136 completed	Low (Depression was measured with a validated, culturally	Some concerns (The trial was prospectively registered and depression was	Some concerns (The study benefits from randomization, blinded outcome	Moderate (Starting from high certainty for a randomized controlled trial, we downgraded

	allocation concealment procedures − for example, central randomization or use of sequentially numbered, opaque, sealed envelopes − were not fully detailed, leaving some uncertainty about protection against selection bias)	collecting depression outcomes and laboratory staff were blinded, but it is unclear whether participants and prescribing clinicians could distinguish active vitamin D_3 from placebo, and concomitant use of other supplements − for example, multivitamins containing vitamin D − was allowed, which could introduce non−differential contamination or deviations from the intended intervention, although such use was similar across arms)	the Edinburgh Postnatal Depression Scale at 4 and 8 weeks postpartum; attrition was moderate and appeared broadly balanced between groups, but the authors did not present a detailed comparison of baseline characteristics for completers versus non−completers, and no multiple−imputation or other sensitivity analyses for missing depression scores were reported)	adapted Persian version of the Edinburgh Postnatal Depression Scale, with good internal consistency and test−retest reliability, and outcome assessors were blinded to treatment allocation, making differential outcome assessment unlikely)	clearly a primary target, but no formal pre−specified statistical analysis plan for depression outcomes was provided, and some analytic choices − for example, categorisation of EPDS scores and repeated measures analysis − could not be fully checked against a protocol, so selective reporting of analyses cannot be entirely excluded, even though the main time points and comparisons were reported transparently)	assessment and use of a validated depression measure, but moderate attrition, limited detail on allocation concealment, potential deviations from intended interventions and incomplete transparency of the analysis plan justify an overall RoB 2 judgement of “some concerns” for the perinatal depression outcome)	one level for risk of bias − overall “some concerns” primarily due to incomplete reporting of allocation concealment, permissive concomitant supplementation and missing data handling − and one level for indirectness of the depression construct − screening scores rather than diagnostic assessment of major depressive disorder in a single−centre Iranian sample of pregnant women with baseline EPDS scores 0−13 − but we considered upgrading by one level because of the moderate−to−large and consistent effect on postpartum Edinburgh Postnatal Depression Scale scores − odds ratio approximately 4.01, 95% confidence interval 2.13−7.55 − and biologically plausible mechanism via

								correction of vitamin D deficiency; overall, this yields a “moderate” GRADE rating for the effect of vitamin D_3 on perinatal depressive symptoms)
**24**	Vellekkatt et al. (2020) [86]	Low (Randomization used a computer−generated stratified permuted block design − block size 4 − to balance inpatient and outpatient status across groups, and allocation concealment was implemented through sequentially numbered opaque sealed envelopes held by an investigator not otherwise involved in enrolment or assessment; baseline demographic and clinical characteristics, including depression scores, did not differ significantly between arms, suggesting successful	Low (The trial was described as double−blind: participants, treating clinicians and outcome raters were unaware of treatment allocation, and both groups received identical−appearing intramuscular injections − vitamin D_3 versus 1 mL normal saline − plus antidepressant treatment as usual. There is no evidence of systematic differences in co−interventions,	Some concerns (Of the 46 randomized participants, 4 or 9% were lost to 12−week follow−up − 1 in the vitamin D_3 arm, 3 in the placebo arm. The primary analysis followed the intention−to−treat principle with last observation carried forward − last observation carried forward, LOCF − imputation for	Low (Depression severity was assessed with the Hamilton Depression Rating Scale−17, a widely validated clinician−rated instrument, administered by trained raters blinded to treatment allocation; quality of life and clinical severity were measured with established scales − Quality of Life Enjoyment and Satisfaction Questionnaire−S	Some concerns (The study reports that analyses were planned a priori and sample size calculations were based on expected change in Hamilton Depression Rating Scale−17, but the publicly accessible materials do not include a detailed, time−stamped statistical analysis plan specifying all primary and secondary endpoints and handling of missing data. Results are	Some concerns (Taking all domains together, the trial shows robust randomization and blinding with validated outcome measures, but the modest attrition managed via last observation carried forward − last observation carried forward, LOCF− and limited transparency regarding a pre−specified analysis plan justify an overall “some concerns” judgment under the Risk of Bias 2 tool for the depression outcome)	Moderate (Under the GRADE framework, randomized controlled trials start at high certainty. We downgraded one level for risk of bias − overall “some concerns” due to missing data handling and incomplete specification of the analytic plan − and one level for imprecision, given the relatively small sample size, n = 46, and wide 95% confidence interval around the pooled OR ≈ 3.70; 95% CI 1.25–10.94. However, the effect size is clinically meaningful, consistent across intention−to−treat and sensitivity analyses, and biologically plausible in the context of vitamin D

	randomization and concealed allocation)	and adherence to oral antidepressants, assessed with the Morisky Green Levine Medication Adherence Scale, was similar between groups at follow−up, making major deviations from intended interventions unlikely)	missing outcome data, and sensitivity analyses were conducted on completers and after excluding telephonic assessments; however, detailed comparisons of baseline characteristics between completers and dropouts are not fully reported)	hort Form and Clinical Global Impression−Sev erity − and although some 12−week assessments were obtained via telephone, there is no indication that measurement properties or blinding were compromised in a way that would differentially affect groups)	presented for the key outcomes described in the methods, yet the absence of a fully pre−specified analysis plan leaves some uncertainty about selective reporting of alternative analyses or time points)		deficiency, providing some support against further downgrading; overall we judge the certainty of evidence for adjunctive vitamin D_3 in major depressive disorder − major depressive disorder, MDD − in this single trial as moderate)

Notes:

- Risk−of−Bias 2 (RoB 2) Methodology: The RoB 2 tool assesses the risk of bias in randomized trials across five fixed domains. Unlike previous versions, RoB 2 is result−specific, focusing on the bias associated with a single estimate of an intervention effect for a particular outcome:
    - Domain 1: Randomization Process: Evaluates the adequacy of the allocation sequence and concealment, and whether baseline imbalances suggest a failure of randomization.
    - Domain 2: Deviations from Intended Interventions: Assesses bias due to the trial context, focusing on either the effect of assignment (Intention−to−Treat) or adherence to the protocol.
    - Domain 3: Missing Outcome Data: Examines the availability of outcome data for all randomized participants and whether the missingness is related to the outcome itself.
    - Domain 4: Measurement of the Outcome: Evaluates the appropriateness of the measurement method and whether the outcome assessors were aware of the intervention received.
    - Domain 5: Selection of the Reported Result: Assesses potential selective reporting of outcomes or analyses based on a pre−specified plan.

- Overall Risk−of−Bias Judgment: Each domain is rated as Low Risk, High Risk, or Some Concerns. The overall judgment for a result is the least favorable (worst) rating across any of the individual domains.

- GRADE Certainty−of−Evidence Ratings: The GRADE framework (Grading of Recommendations Assessment, Development, and Evaluation) is used to rate the confidence in the body of evidence for each outcome:
  - Initial Certainty: Evidence from randomized controlled trials starts with a High default rating (4 points).
  - Downgrading Factors: Certainty is reduced (by −1 or −2 levels) based on five domains:
    1. Risk of Bias: Informed by the Cochrane RoB 2 assessments.
    2. Inconsistency: Unexplained heterogeneity in results across trials.
    3. Indirectness: Differences in populations, interventions, or outcomes compared to the research question.
    4. Imprecision: Results with wide confidence intervals or small sample sizes.
    5. Publication Bias: Selective publication of studies based on the direction or strength of their results.
  - Final Ratings: Levels are classified as High, Moderate, Low, or Very Low, indicating the degree to which we are confident that the true effect lies close to the estimate.

Table S6. Methodological quality appraisal of observational studies using the Newcastle−Ottawa Scale (NOS) and Certainty−of−Evidence judgments under the GRADE framework.

Record ID	Study	GRADE Certainty of Evidence (Rating & Rationale)	NOS – Selection (0−4 Stars; Judgment & Justification)	NOS – Comparability (0−2 Stars; Judgment & Justification)	NOS – Exposure/Outcome (0−3 Stars; Judgment & Justification)	Total NOS Score (0−9; Judgment & Justification)
**1**	Ali et al., (2019) [44]	Low<br>Observational design; limited event count; temporality and outcome ascertainment are appropriate; residual confounding cannot be excluded.	4<br>Representative cohort; exposure objectively measured; outcome absent at baseline; non−exposed drawn from same population.	2<br>Adjusted for key confounders (age, sex, maternal ethnicity, socioeconomic proxies).	2<br>Valid outcome ascertainment; follow−up likely adequate but potential under−ascertainment for later ASD diagnoses.	8<br>Good−quality prospective cohort with minor limitations in follow−up window.
**2**	Altun et al., (2018) [46]	Low<br>Observational design with potential residual confounding; objective biomarker assays and matched controls support internal validity.	4<br>Clear ASD case definition, representativeness of cases, appropriate control selection from same source, outcome − ASD absent in controls.	1<br>Matching on age, sex, and season; limited adjustment for dietary exposure or socioeconomic factors.	2<br>Exposure (biomarker) ascertained by ELISA with identical methods in cases and controls; non−response not fully characterized.	7<br>Good−quality case−control study with biomarker focus; some unmeasured confounding and response characterization limits remain.
3	Anmella et al. (2025) [47]	Low<br>Observational, single−centre cross−sectional design;	3<br>Consecutive child and adolescent inpatients	1<br>Multivariable logistic regression models	2<br>Exposures (serum folate and vitamin B_{12}, insufficiency and	6<br>Moderate−quality observational study

		temporality between folate/B_{12} status and psychiatric diagnoses cannot be established; however, large sample (N = 729) and precise effect estimates for key outcomes. Residual confounding by diet, socioeconomic status, BMI and psychotropic medication use is likely, and no information is provided on inter−rater reliability of diagnoses; no upgrading criteria (large effect, dose−response) clearly fulfilled for the vitamin−outcome associations.	from a tertiary hospital unit; main psychiatric diagnoses defined using DSM−5 and grouped into explicit categories; all comparison groups (other diagnoses) drawn from the same underlying inpatient population. Serum folate and B_{12} measured at admission as part of a standardized ward protocol. Representativeness is limited to a single hospital setting and not clearly population−based.	adjust for key demographic confounders (age, sex) and include vitamin levels/insufficiency and some clinical covariates (e.g., non−psychiatric comorbidities) in model building. Important potential confounders (dietary intake, socioeconomic status, BMI, medication profiles) are not measured or controlled, leaving residual confounding.	deficit) objectively ascertained with validated electrochemiluminescence immunoassays in a single hospital laboratory with documented quality control. Outcomes are psychiatric diagnoses made by specialists according to DSM−5 and consistently categorized. However, handling of missing laboratory data or incomplete records is only briefly reported, non−response is not characterized, and no formal assessment of diagnostic reliability is provided.	with strong, objective biomarker ascertainment and clear diagnostic categorization but limited external representativeness, incomplete control for confounding and an inherently cross−sectional design that precludes causal inference.
**4**	Boerman et al., (2016) [48]	Very low<br>Cross−sectional design without concurrent control group; potential residual confounding; indirect comparison to general population.	3<br>Regional outpatient sample with clear diagnostic grouping; no concurrent recruited control group.	2<br>Models adjusted for season and ethnicity; diagnostic group considered.	2<br>Standardized assay for 25(OH)D; outcome defined by prespecified threshold; non−response and missingness minimally detailed.	7<br>Good laboratory measurement and prespecified threshold; limitations from design and control selection.
**5**	Dhiman et al., (2021) [52]	Low<br>Observational design with potential residual confounding; large sample and dose−response	4<br>Clear case definition (EPDS > 10), hospital−based	2<br>Age and BMI matching; multivariable adjustment for key	3<br>Biomarkers assayed via validated ELISA; identical ascertainment across groups;	9<br>High methodological quality for a cross−sectional

		patterns support consistency but do not eliminate confounding.	consecutive sampling, appropriate control selection from same source population, controls defined as EPDS ≤ 10.	socio−demographic and nutritional covariates (e.g., socioeconomic status, marital dissatisfaction, pregnancy planning, delivery type).	comparable response/retention derived from archived cohort with re−consent.	case−control study with comprehensive biomarker assessment and appropriate adjustment.
**6**	Endres et al., (2016) [54]	Very low<br>Observational design; historical control without matching for key confounders (season, age, BMI, sunlight exposure); imprecision for ASD subgroup (*n* = 23).	3<br>Clear inclusion; well−defined patient groups; ascertainment of exposure (25(OH)D) objective; control cohort external and historical.	0<br>No multivariable adjustment or matching to the reference cohort for seasonality, BMI, or age; bivariate comparisons only.	2<br>Validated chemiluminescence immunoassay; consistent measurement across groups; standardized thresholds (<20 and <10 ng/mL).	5<br>Moderate quality for biomarker prevalence study; main limitation is uncontrolled confounding by season and demographics inherent to the historical control.
7	Erensoy (2020) [55]	Very low<br>Cross−sectional design with no healthy control group and no clear temporality between vitamin status and mood disorders; modest sample (N = 150) limits precision and makes effect estimates sensitive to random error. Potential selection bias from a single tertiary outpatient clinic, and important confounders (diet, socioeconomic status, comorbidities beyond major exclusions, medication profiles) are not measured or adjusted. No upgrading domains (large,	2<br>Clinical sample of 77 anxiety and 73 depression patients recruited consecutively from a single neuropsychiatry clinic; diagnoses based on BDI−II and BAI cut−offs validated in Turkish populations. However, there is no population−based frame or healthy	0<br>Analyses are primarily unadjusted comparisons (anxiety vs depression, sex and severity subgroups) and simple correlations between vitamin levels and BDI−II/BAI scores. Apart from stratification by age, sex and severity, there is no multivariable adjustment for key confounders (diet, BMI, socioeconomic status, smoking, alcohol, other	2<br>Exposures (serum vitamin B_{12} and folic acid) measured after overnight fasting with a standardized Roche Cobas e411 platform; deficiency cut−offs are pre−specified (B_{12} < 200 pg/mL; folate < 2.2 ng/mL). Mood symptoms are quantified with validated instruments (BDI−II, BAI), administered under psychologist supervision. Nonetheless, outcome classification (anxiety vs depression) is based solely on	4<br>Fair−quality cross−sectional pilot study with robust laboratory assessment and validated symptom scales, but important limitations in selection, lack of control group, absence of adjustment for confounders and cross−sectional design constrain internal validity and causal inference.

		consistent effects or dose–response) are robustly fulfilled.	reference group, and representativeness is limited to treatment–seeking adults. Inclusion/exclusion criteria (e.g., exclusion of hepatic/renal disease, diabetes, vitamin supplementation) are clearly specified, but selection remains clinic–based and potentially biased.	medical conditions), so comparability between groups is not ensured.	self–report inventories rather than structured diagnostic interviews, and handling of missing data or non–participation is not described.	
**8**	Esnafoğlu & Yaman (2017) [57]	Low<br>Observational design with potential residual confounding (season, diet, sunlight exposure); precise assays and consistent direction of effects prevent further downgrades.	3<br>Adequate case definition and source population; hospital–based controls; controls confirmed free of psychiatric diagnosis.	1<br>Groups similar on age/sex; no multivariable adjustment for key confounders (e.g., seasonality, diet, socioeconomic factors).	2<br>Standardized laboratory methods applied identically in both groups; non–response details limited.	6<br>Good biomarker measurement quality; primary limitations are control selection and limited control of confounding.
**9**	Esnafoglu & Ozturan (2020) [56]	Low<br>Observational design with potential residual confounding (diet, socioeconomic factors, sunlight); precise, standardized assays and consistent direction of effects.	3<br>Clear case definition and source population; hospital–based controls; contemporaneous recruitment.	1<br>Groups similar in age/sex; no multivariable adjustment for key confounders beyond basic matching.	2<br>Standardized chemiluminescent assays applied identically; fasting morning blood draws; identical timing to mitigate seasonality (October–May).	6<br>Moderate quality; limitations primarily in control selection and confounding control.
**10**	Fabrazzo et al., (2022) [58]	Low	3	2	2	7

		Cross−sectional, single center; although multivariable models were used, residual confounding and temporality remain	Clearly defined inpatient and outpatient subgroups from the same clinical network; objective biomarker ascertainment	Models adjusted for key covariates (e.g., sex, age, marital status, education, diagnosis, pharmacological treatments)	Uniform laboratory methods across groups; objective outcomes; no follow−up component	Good biomarker assessment and reasonable covariate control; main limitations are design and residual confounding
**11**	Huang et al., (2018) [62]	Low<br>Evidence arises from a cross−sectional observational analysis, which is inherently limited for inferring temporality and causality between vitamin status and depression; although the study benefits from a large, nationally representative sample, standardized laboratory assays and validated depression assessment (9−item Patient Health Questionnaire, PHQ−9), residual confounding (for example by diet, comorbidities and supplementation practices) and reverse causation (depression influencing vitamin status) cannot be excluded. Certainty is further limited by the use of single time−point vitamin measurements and the absence of prospective follow−up, whereas strengths include careful adjustment for major demographic and socioeconomic covariates and	3<br>Large, nationally representative U.S. sample of adults aged 20−85 years from National Health and Nutrition Examination Survey (NHANES) 2005−2006; sampling frame and weighting procedures support representativeness; clear inclusion and exclusion criteria (for example, exclusion of pregnant women, participants < 20 years and those with missing key data). One star is withheld because participation and non−response characteristics are not fully detailed, and cross−sectional design	2<br>Analyses adjust for a broad set of potential confounders, including age, sex, body mass index (BMI), family poverty−income ratio (PIR), race/ethnicity, education, marital status, smoking history, alcohol use and examination time, thereby controlling for many sociodemographic and lifestyle factors that may influence both vitamin status and depression risk.	2<br>Exposure measurement is strong: serum vitamins were quantified with validated laboratory methods (radioassay, high−performance liquid chromatography, HPLC, and liquid chromatography−tandem mass spectrometry, LC−MS/MS) and handled with standardized quality−control procedures; outcome measurement uses the validated 9−item Patient Health Questionnaire (PHQ−9) with a widely accepted cut−off (≥ 10) for depression. However, depression is based on a single self−report assessment at one time point, and there is no longitudinal follow−up to characterize outcome stability or misclassification over time, so one star is withheld.	7<br>Overall good−quality cross−sectional analysis with strong exposure and outcome measurement and extensive adjustment for confounding (3/4 selection, 2/2 comparability, 2/3 outcome), but constrained by the limitations of cross−sectional design, potential residual confounding and lack of repeated measures.

		consistent patterns in sex−stratified analyses for folate and vitamin B_{12}.	precludes establishing that exposure preceded outcome.			
**12**	Laird et al., (2023) [66]	Low<br>The certainty of evidence linking deficient−low vitamin B_{12} (cobalamin) status to incident depressive symptoms is rated low under the Grading of Recommendations, Assessment, Development and Evaluations (GRADE) framework. As an observational cohort, the starting level is low, with upgrading not applied because there is evidence from only a single national cohort, the effect size is modest (odds ratio = 1.51, 95% confidence interval 1.01−2.27) and residual confounding (for example, unmeasured dietary factors, inflammatory markers or genetic determinants of vitamin B_{12} metabolism) cannot be fully excluded. However, the direction of effect is consistent across multiple models and sensitivity analyses, temporality is clear (vitamin B_{12} status measured at baseline precedes depression onset) and the association is biologically plausible given the role of vitamin B_{12} in one−carbon	4<br>Selection is judged as very good. (1) Representativeness of the exposed cohort: participants were drawn from a nationally representative, community−dwelling sample of older adults in Ireland using a stratified cluster sampling of residential addresses, and the analytic subset with blood samples and follow−up is broadly representative of the target population. (2) Selection of the non−exposed cohort: comparison groups (normal vitamin B_{12} status versus deficient−low and low−normal vitamin B_{12} status) were drawn from the same	2<br>Comparability is judged as strong. The primary multivariable models adjusted for a comprehensive set of potential confounders, including age, sex, educational attainment, body−mass index, smoking status, alcohol use via Cut down, Annoyed, Guilty, Eye−opener (CAGE) score, physical activity level, chronic disease burden, cardiovascular disease, cognitive impairment, subthreshold depressive symptoms at baseline, antidepressant use and vitamin D status (25−hydroxyvitamin D, 25(OH)D). This adjustment strategy addresses major demographic, clinical, lifestyle and nutritional factors that could	2<br>Outcome domain is judged as good but not perfect. (1) Assessment of outcome: incident depressive symptoms were measured using the Center for Epidemiological Studies Depression Scale 8−item (CES−D−8), a validated and reliable screening instrument for depression in older adults within TILDA, but not a diagnostic clinical interview; outcome assessment was standardized but self−reported, leading to a small risk of misclassification (one star). (2) Was follow−up long enough for outcomes to occur?: the 4−year follow−up window (waves 1−3) is adequate for incident depressive symptoms to develop in older adults (one star). (3) Adequacy of follow−up of cohorts: although TILDA experiences attrition, the study excluded those without complete depression follow−up and did not report	8<br>Overall, the study is rated as high quality under the Newcastle−Ottawa Scale (NOS), with full marks in the Selection and Comparability domains and a minor shortcoming in the Outcome domain due to reliance on a screening scale rather than diagnostic interview for depression and incomplete reporting of loss−to−follow−up patterns. The strong cohort design, objective biomarker exposure assessment, rigorous confounder adjustment and adequate follow−up support confidence in the direction and approximate magnitude of the association between

	metabolism and monoamine neurotransmitter synthesis. No serious concerns were identified for indirectness or inconsistency, but imprecision and residual confounding preclude upgrading above low certainty.	underlying TILDA cohort. (3) Ascertainment of exposure: plasma vitamin B_{12} (cobalamin) and plasma folate were measured at baseline by validated microbiological assays with good inter−assay precision, providing objective and reliable exposure assessment. (4) Demonstration that outcome of interest was not present at the start of the study: participants with clinically significant depressive symptoms at baseline, defined by Center for Epidemiological Studies Depression Scale 20−item (CES−D−20) score ≥ 16, were excluded, ensuring that incident depressive symptoms were newly developed during follow−up.	confound the relationship between vitamin B12 status and incident depressive symptoms, thereby meriting two stars for comparability.	major differential loss−to−follow−up by vitamin B12 status; however, the lack of detailed comparison between responders and non−responders and potential selection bias from complete−case analysis precludes awarding the third star.	deficient−low vitamin B_{12} status and incident depressive symptoms, within the limits of observational inference.

**13**	Madley−Dowd et al., (2022) [68]	Low<br>Evidence starts at low certainty for observational designs. The large sample, prospective measurement of maternal 25−hydroxyvitamin D [25(OH)D] and rigorous, multi−source autism case ascertainment support internal validity, but there is residual concern about selection bias from missing data (notably for trait measures), limited representation of severe vitamin D deficiency and unmeasured or imperfectly measured confounders (for example, lifestyle, sunlight exposure). Precision for autism diagnosis is modest, with relatively few cases and confidence intervals spanning the null. No upgrading is applied because there is no signal of a large effect, no clear dose−response and Mendelian randomization analyses are null but imprecise.	4<br>Representativeness of the exposed cohort is high: ALSPAC is a well−described regional birth cohort with defined catchment and recruitment procedures; exposure (maternal 25−hydroxyvitamin D [25(OH)D]) was measured prospectively from stored serum samples using validated laboratory methods; autism diagnosis was absent at baseline (pregnancy) by design; and comparison groups (different 25(OH)D levels) arise from the same underlying source population.	2<br>Analyses adjust for an extensive set of a priori confounders, including offspring sex, maternal age, parity, pre−pregnancy body−mass index (BMI), smoking in pregnancy, education, occupational class and financial difficulties, in addition to sensitivity analyses restricted to European ancestry to reduce population stratification. Multiple imputation and Mendelian randomization further address potential bias sources, yielding maximal comparability given available data.	2<br>Outcomes are ascertained using validated, multi−source case finding for autism diagnosis and well−characterised trait measures; follow−up duration into childhood is adequate to capture most diagnoses; and missing outcome data are handled with multiple imputation. However, attrition is non−trivial, and children with more severe traits were somewhat less likely to have complete records, raising concerns about selection related to outcome despite mitigation via imputation.	8<br>Overall, this is a high−quality prospective cohort with strong exposure and outcome measurement and careful confounder control, but modest limitations in follow−up completeness and selection related to missing data preclude the maximum NOS score.
**14**	Okasha et al., (2020) [74]	Low<br>Under the Grading of Recommendations, Assessment, Development and Evaluations (GRADE) framework, observational case−control	2<br>One star for selection of cases: patients with major depressive disorder (MDD) and schizophrenia were	0<br>No stars for comparability: although groups were matched on age and gender and assessed in the same	2<br>Two stars for exposure/outcome: serum 25−hydroxyvitamin D [25(OH)D] was measured using a standardized	4<br>Overall, this is a small, single−centre cross−sectional case−control study with reasonably

	evidence starts at low certainty. This study is further limited by small sample size (N = 60 total, 20 per group), convenience sampling from a single psychiatric institute, and lack of adjustment for potential confounders such as body mass index, dietary vitamin D intake, and lifestyle factors (for example, physical activity, sun exposure, veiling). The cross−sectional design precludes temporal inference, and the confidence intervals around the odds ratios are wide, reflecting imprecision. There is no strong indirectness with respect to adult major depressive disorder (MDD) and schizophrenia populations, but overall certainty remains low.	clinically diagnosed using the Structured Clinical Interview for Diagnostic and Statistical Manual of Mental Disorders, Fourth Edition (DSM−IV) Axis I Disorders (SCID−I), which is an appropriate and validated diagnostic tool. One star for definition and selection of controls: healthy volunteers without psychiatric or relevant medical illnesses were recruited and matched for age, gender, and social standard. However, participants were drawn from a single tertiary−care setting by convenience sampling, representativeness of cases and controls with respect to the wider population is uncertain, and there is	time frame, there was no multivariable adjustment for important confounders such as body mass index, dietary vitamin D intake, physical activity, sun exposure habits, season, or psychotropic medication use. Potential confounding by these factors limits internal validity of the vitamin D−psychiatric diagnosis association estimates.	enzyme−linked immunosorbent assay (ELISA), applied equally to all groups, with deficiency categories clearly defined. Psychiatric diagnoses were based on a structured clinical interview (SCID−I). However, no blinding of laboratory staff to case−control status is explicitly reported, and details on repeat measurements or quality control procedures for the assay are limited, so one star is withheld.	well−defined cases and controls and objective biomarker measurement, but with limitations in representativeness, lack of adjustment for confounders, and incomplete reporting of laboratory quality control. The resulting Newcastle−Ottawa Scale (NOS) score of 4/9 reflects moderate−to−high risk of bias for etiological inference.

			no explicit statement that cases were incident rather than prevalent.			
**15**	Petruzzelli et al., (2020) [78]	Very low<br>Under the Grading of Recommendations, Assessment, Development and Evaluations (GRADE) framework, observational studies start at low certainty. Here, certainty is downgraded for several reasons. Risk of bias is substantial: the sample is a single−centre inpatient cohort, selection procedures are not clearly consecutive, and the non−ASD group comprises heterogeneous neurological and psychiatric conditions rather than healthy controls, limiting representativeness and introducing potential selection and indication biases. Confounding is only partially addressed; the logistic regression adjusts for age and sex, but not for critical factors such as season and sunlight exposure, dietary habits, body mass index, physical activity, socioeconomic status, or antiepileptic and psychotropic medications that could affect vitamin D levels and autism	3<br>Three stars in the selection domain. (1) Case definition: autism spectrum disorder (ASD) cases were rigorously defined using Diagnostic and Statistical Manual of Mental Disorders, Fifth Edition (DSM−5) criteria confirmed with standardized instruments (Autism Diagnostic Interview−Revised, ADI−R; Autism Diagnostic Observation Schedule, Second Edition, ADOS−2) and expert clinical consensus (star awarded). (2) Representativeness of the cases: ASD cases were drawn from a tertiary hospital inpatient population	1<br>One star for comparability: the multivariable logistic regression model adjusted for age and sex, which are important potential confounders of vitamin D status and autism spectrum disorder (ASD) diagnosis (one star awarded). No further adjustment was made for other relevant variables (for example, season, outdoor activity, diet, body mass index, medications), and there was no matching on these factors; therefore the second comparability star is not awarded.	2<br>Two stars in the exposure/outcome domain. Serum 25−hydroxyvitamin D [25(OH)D] concentrations were measured using a chemiluminescence immunoassay, the same method applied to both autism spectrum disorder (ASD) and non−ASD groups, and categorized according to established clinical cut−offs (star awarded). Outcome (autism spectrum disorder, ASD vs non−ASD) was based on clinical diagnosis with standardized instruments for ASD and thorough neuropsychiatric assessment for non−ASD diagnoses. However, details about blinding of laboratory personnel to ASD status, quality control procedures for the assay, and whether vitamin D measurements preceded or followed inpatient admission for acute issues are	6<br>The overall Newcastle−Ottawa Scale (NOS) score of 6 out of 9 reflects moderate risk of bias. Strengths include rigorous definition of autism spectrum disorder (ASD) cases with standardized diagnostic tools, objective and uniform measurement of serum 25−hydroxyvitamin D [25(OH)D], and adjustment for age and sex. Limitations include single−centre, inpatient, hospital−based sampling; heterogeneous non−ASD control diagnoses; incomplete control of key confounders; and imprecision due to modest sample size.

	spectrum disorder (ASD) risk. The confidence interval around the main odds ratio (OR = 10.31; 95% confidence interval, CI 1.96–54.22) is very wide, reflecting imprecision and limited sample size. Indirectness is also present, as the comparison group is neuropsychiatric rather than non−psychiatric controls. Taken together, these issues support a rating of very low certainty regarding a causal role of vitamin D deficiency in autism spectrum disorder (ASD), despite the large point estimate.	rather than a community or population−based sample; while clinically relevant, this may not be fully representative of the broader ASD population (star withheld). (3) Selection of controls: non−ASD inpatients with other neurological and psychiatric diagnoses were recruited from the same unit and time frame, representing a reasonable hospital−based control group (star awarded). (4) Definition of controls: the non−ASD group was clearly defined as lacking autism spectrum disorder (ASD), but included a mixture of neurodevelopmental, psychiatric and neurological		limited, and potential misclassification is possible, especially regarding seasonality and supplementation history (one star withheld).	These constraints temper confidence in the magnitude and generalizability of the observed association between vitamin D deficiency and autism spectrum disorder (ASD).

			conditions, not healthy controls; nonetheless, they were drawn from the same source population and assessed with similar procedures (star awarded).			
**16**	Shahini et al., (2022) [81]	Very low<br>Under the Grading of Recommendations, Assessment, Development and Evaluation (Grading of Recommendations, Assessment, Development and Evaluation, GRADE) framework, observational evidence starts at low certainty. The certainty is downgraded one level for risk of bias because of the small, single−centre convenience sample, lack of explicit consecutive recruitment of cases, absence of multivariable adjustment beyond age and sex matching, and substantial residual confounding (for example, body mass index, diet, smoking, physical activity, sunlight exposure, and antipsychotic dose were not controlled). A further downgrade is applied for imprecision, as the odds ratio (OR ≈ 2.30) has a wide	3<br>Case definition was adequate (schizophrenia diagnosed by a psychiatrist with semi−structured interviews based on Diagnostic and Statistical Manual of Mental Disorders, Fifth Edition, DSM−5, with exclusion of other psychiatric and relevant physical illnesses), and controls were clearly defined as psychiatrically healthy individuals with no personal or family history of mental disorders. Both cases and	1<br>One comparability star is awarded because age− and sex−matching of cases and controls was implemented, reducing confounding by these key demographic variables. No additional matching or statistical adjustment for other important confounders (for example, body mass index, marital status, smoking, nutritional status, season of blood draw, or antipsychotic regimen) was reported, so the second comparability star is not granted.	2<br>Serum vitamin D and homocysteine concentrations were measured using standardized commercial kits applied uniformly to cases and controls, which supports reliable and comparable exposure assessment (one star). Schizophrenia diagnosis in cases was based on structured clinical evaluation, while controls were screened for absence of psychiatric disorders, providing reasonably robust outcome ascertainment (second star). However, there is no information about blinding of laboratory personnel to case−control status, and non−differential measurement error related to pre−analytical variables (for example, time of	6<br>The total Newcastle−Ottawa Scale (NOS) score of 6 out of 9 indicates moderate methodological quality. Strengths include clear diagnostic criteria for schizophrenia, matched healthy controls from the same region, and objective biomarker measurements. Limitations relate to non−representative sampling, lack of comprehensive adjustment for confounding, small sample size, and the cross−sectional nature

		95% confidence interval (lowerOR ≈ 0.80, upperOR ≈ 6.61) due to the modest sample size. Indirectness is also present since vitamin D deficiency and schizophrenia were measured cross−sectionally, precluding temporal ordering. Taken together, these limitations support a very low certainty rating for a causal association between vitamin D deficiency and schizophrenia, despite the suggestive direction of effect.	controls were drawn from the same hospital catchment area, but recruitment used convenience sampling and the inpatient sample may not be fully representative of the broader schizophrenia population, so the representativeness star is withheld. Overall, three stars are awarded in the selection domain.		sampling, storage conditions beyond −20 °C) cannot be excluded; therefore, one exposure/outcome star is withheld.	of the case−control comparison, which constrains inference about temporality and the magnitude of association between vitamin D deficiency and schizophrenia.
**17**	Sourander et al., (2021) [82]	Moderate<br>Under the Grading of Recommendations, Assessment, Development and Evaluation (Grading of Recommendations, Assessment, Development and Evaluation, GRADE) framework, observational evidence starts at low certainty. One level is upgraded because of the strong prospective design (maternal 25−hydroxyvitamin D [25(OH)D] measured during early pregnancy, long before autism spectrum disorder (autism spectrum disorder, ASD) diagnosis), large nationwide sample, and	4<br>Case definition: autism spectrum disorder (autism spectrum disorder, ASD) cases identified from the Finnish Care Register for Health Care using International Classification of Diseases (International Classification of Diseases, ICD−9 and ICD−10) diagnostic codes, with	2<br>Two stars are awarded for comparability because the conditional logistic regression models adjusted for a comprehensive set of confounders identified a priori as related to both maternal vitamin D status and offspring autism spectrum disorder (autism spectrum disorder, ASD), including maternal age, gestational age,	3<br>Exposure assessment: maternal 25−hydroxyvitamin D [25(OH)D] concentrations were measured using the same validated immunoassay for all cases and controls, with analysts blinded to case−control status and quality control samples confirming excellent reproducibility, justifying one star. Outcome ascertainment: autism spectrum disorder (autism spectrum disorder, ASD) diagnoses were obtained from the national care register,	9<br>The total Newcastle−Ottawa Scale (Newcastle−Ottawa Scale, NOS) score of 9 out of 9 reflects excellent methodological quality: a population−based nested case−control design with prospective exposure ascertainment, objective biomarker measurement,

	high−quality national registers with validated autism spectrum disorder (autism spectrum disorder, ASD) diagnoses, which reduce risk of bias and reverse causation. A further upgrade is supported by the consistent dose−response pattern across continuous, quintile, and clinical categories of maternal 25−hydroxyvitamin D [25(OH)D], with higher exposure associated with lower risk. The certainty is not rated as high due to residual confounding by unmeasured factors (for example, maternal body mass index, dietary patterns, supplement use, or genetic variants), reliance on a single 25−hydroxyvitamin D [25(OH)D] measurement, and restriction of autism spectrum disorder (autism spectrum disorder, ASD) ascertainment to cases seen in specialized services. Overall, the association between low maternal 25−hydroxyvitamin D [25(OH)D] and increased offspring autism spectrum disorder (autism spectrum disorder, ASD) risk is judged to be supported by moderate−certainty evidence.	previously demonstrated high specificity of registry−based childhood autism diagnoses (one star). Representativeness: all eligible singleton live births with autism spectrum disorder (autism spectrum disorder, ASD) diagnoses in Finland during 1987–2004 with available maternal serum in the Finnish Maternity Cohort were included, yielding a nationwide, population−based case series (second star). Selection of controls: controls without autism spectrum disorder (autism spectrum disorder, ASD) or intellectual disability (intellectual disability, ID) were randomly selected from the same national birth	gestational week of blood draw, season of blood collection, maternal smoking, immigration status, maternal psychopathology, and maternal substance abuse; paternal psychiatric diagnoses and other perinatal risk factors were considered as additional covariates in model building. Matching on sex and date of birth further controlled for key demographic factors.	which has been validated and is expected to have high specificity for childhood autism and related conditions (second star). Non−response and missingness: virtually all eligible case−control pairs had sufficient serum for analysis, and missing data for covariates were minimal; the nested case−control design within a defined birth cohort minimizes differential non−response, supporting a third star.	rigorous case and control selection, and extensive confounder adjustment. Remaining concerns include unmeasured residual confounding (for example, maternal body mass index, diet, supplement use, or other micronutrients) and the use of a single maternal 25−hydroxyvitamin D [25(OH)D] measurement, but these limitations do not materially undermine the internal validity of the main association.

			cohort via the Population Register Centre, ensuring derivation from the same underlying population (third star). Ascertainment of exposure: maternal 25−hydroxyvitamin D [25(OH)D] was measured using a standardized chemiluminescence microparticle immunoassay on stored serum samples, with blinded laboratory staff and documented low assay coefficient of variation (≈ 2.1%), providing high−quality objective exposure assessment (fourth star).			
**18**	Terock et al., (2020) [83]	Very low<br>Under the Grading of Recommendations, Assessment, Development and Evaluation (Grading of Recommendations, Assessment, Development and Evaluation, GRADE) framework, observational evidence starts at	3<br>Selection is strong but not fully cohort−like. (1) Representativeness: the Study of Health in Pomerania (Study of Health in Pomerania,	2<br>Two stars for comparability: multivariable logistic regression adjusted for a prespecified set of key confounders plausibly related to both vitamin	3<br>(1) Exposure ascertainment: serum 25−hydroxyvitamin D [25(OH)D] was measured in a central laboratory under standardized conditions with blinded technicians and documented coefficients of	8<br>Overall Newcastle−Ottawa Scale (Newcastle−Ottawa Scale, NOS) score indicates good methodological

	low certainty. For this study, the cross−sectional design with simultaneous assessment of serum 25−hydroxyvitamin D [25(OH)D] and posttraumatic stress disorder (posttraumatic stress disorder, PTSD) precludes establishing temporal precedence and leaves substantial potential for reverse causation (for example, posttraumatic stress disorder (posttraumatic stress disorder, PTSD) leading to behavioural changes that lower vitamin D levels). Although the population−based sampling frame, standardized outcome assessment, and comprehensive adjustment for confounders (sex, age, waist circumference, physical inactivity, schooling, depressive symptoms, season) are strengths, residual confounding by unmeasured lifestyle and nutritional factors (for example, detailed sun exposure, dietary intake, non−prescribed supplementation) remains likely. Precision is limited by the relatively small number of posttraumatic stress disorder (posttraumatic stress disorder, PTSD) cases (n = 63), and the immunoassay used showed	SHIP) is a population−based cohort in northeastern Germany, and the analytic sample includes all eligible adults with at least one traumatic event after exclusion of subjects with extreme 25−hydroxyvitamin D [25(OH)D] values, prescribed vitamin D treatment, severe renal disease, primary hyperparathyroidism, or missing data, supporting good representativeness of trauma−exposed community adults (one star). (2) Selection of non−exposed: comparison participants are drawn from the same trauma−exposed source population but do not meet criteria for posttraumatic stress disorder (posttraumatic stress	D status and posttraumatic stress disorder (posttraumatic stress disorder, PTSD), including sex, age, waist circumference, physical inactivity, years of schooling, depressive symptoms, and season of blood sampling, thereby partially controlling for lifestyle, body composition, and affective comorbidity. Additional models included trauma load and propensity score matching to test robustness, further supporting adequate control of measured confounding.	variation, justifying one star despite assay variability (first star). (2) Outcome assessment: posttraumatic stress disorder (posttraumatic stress disorder, PTSD) was assessed using the PTSD module of the Structured Clinical Interview for the Diagnostic and Statistical Manual of Mental Disorders, Fourth Edition (Diagnostic and Statistical Manual of Mental Disorders, Fourth Edition, DSM−IV), administered by trained and certified examiners using a widely accepted research instrument (second star). (3) Non−response and missing data: the analytic sample is clearly defined, with exclusions transparently reported and no indication of substantial differential non−response between exposed and non−exposed groups, supporting adequate completeness of outcome data (third star).	quality for a cross−sectional observational study: population−based sampling, objective biomarker measurement, standardized diagnostic assessment of posttraumatic stress disorder (posttraumatic stress disorder, PTSD), and extensive adjustment for confounding. The lack of temporality between 25−hydroxyvitamin D [25(OH)D] and posttraumatic stress disorder (posttraumatic stress disorder, PTSD), reliance on self−reported physical activity and schooling as proxies for lifestyle and socioeconomic status, and absence of detailed data on diet and sun exposure are the main limitations that preclude a higher

		relatively high coefficients of variation, increasing measurement error for 25−hydroxyvitamin D [25(OH)D]. No upgrading was applied because the effect size for vitamin D deficiency is moderate (odds ratio ≈ 2,0) and there is no clear evidence of a consistent dose−response across independent cohorts. Overall, the certainty of evidence for the association between vitamin D deficiency and posttraumatic stress disorder (posttraumatic stress disorder, PTSD) in this single cross−sectional study is rated very low.	disorder, PTSD), satisfying the requirement that cases and non−cases come from the same underlying cohort (second star). (3) Ascertainment of exposure: serum 25−hydroxyvitamin D [25(OH)D] was measured using a standardized automated chemiluminescence immunoassay with quality control, providing objective biochemical exposure assessment (third star). (4) Demonstration that outcome of interest was not present at start of study is not applicable in a cross−sectional design, so a fourth selection star is not awarded.			score and underpin the very−low GRADE certainty rating.
**19**	Van der Leeuw et al., (2020) [84]	Very low<br>Under the Grading of Recommendations, Assessment,	4<br>Case definition and representativeness:	2<br>Analyses comparing vitamin D levels	3<br>Exposure ascertainment: serum 25−hydroxyvitamin D3	9<br>Overall, the study achieves a high

	Development and Evaluation (Grading of Recommendations, Assessment, Development and Evaluation, GRADE) framework, observational evidence starts at low certainty. Here, the cross−sectional design with concurrent assessment of psychotic disorder and 25−hydroxyvitamin D [25(OH)D] status precludes establishing whether low vitamin D preceded illness onset or resulted from illness−related behaviors (for example, social withdrawal, low outdoor activity), introducing serious concerns about reverse causation. Although case and control groups were well defined and analyses adjusted for major confounders (sex, age, body mass index, smoking, ethnicity, season), residual confounding by unmeasured lifestyle and nutritional factors (dietary vitamin D intake, supplement use, detailed sun−exposure patterns) is likely, and the odds ratio used in this meta−analysis is derived from a crude cross−tabulation rather than fully adjusted logistic models. The within−patient associations between vitamin D and symptom	patients were recruited from specialized mental health services across multiple regions and met Diagnostic and Statistical Manual of Mental Disorders, Fourth Edition (Diagnostic and Statistical Manual of Mental Disorders, Fourth Edition, DSM−IV) criteria for non−affective psychotic disorders based on the Comprehensive Assessment of Symptoms and History (Comprehensive Assessment of Symptoms and History, CASH), providing a clear and validated case definition from a clinically relevant spectrum (first star). The recruitment strategy within the Genetic Risk and	between patients and controls adjusted for a prespecified set of confounders: sex, age, body mass index (body mass index, BMI), smoking (cigarettes per day), ethnicity and sampling season, all of which are plausibly associated with both 25−hydroxyvitamin D [25(OH)D] status and psychotic disorder, justifying two stars for comparability.	[25(OH)D3] was measured by a validated laboratory protocol using solid−phase extraction and liquid chromatography−tandem mass spectrometry (liquid chromatography−tandem mass spectrometry, LC−MS/MS), with samples stored at −80 °C before analysis and the same method applied to patients and controls (first and second stars). Non−response and missing data: only 22 of 651 original plasma samples were unavailable due to insufficient volume or laboratory error, leaving 629 analyzable participants; exclusions were transparently reported, and there is no indication of differential sample loss between groups, supporting a low risk of bias from non−response (third star).	Newcastle−Ottawa Scale (Newcastle−Ottawa Scale, NOS) score based on rigorous case and control definition, community−based recruitment, high−quality biomarker measurement and appropriate adjustment for several key confounders. The main limitations relate to the cross−sectional design and limited detail on unmeasured lifestyle factors that could influence 25−hydroxyvitamin D [25(OH)D], which are captured in the GRADE certainty rating rather than the Newcastle−Ottawa Scale (Newcastle−Ottawa Scale, NOS) total.

severity are small, and no dose–response across multiple exposure levels is formally quantified in odds–ratio form. No upgrading is warranted, so overall certainty for the association between vitamin D insufficiency and psychotic disorder in this single cross–sectional study is judged to be very low.	Outcome of Psychosis (Genetic Risk and Outcome of Psychosis, G.R.O.U.P.) study yields a reasonably representative sample of treated psychosis patients in the Netherlands (second star). Controls were drawn from the same geographic catchment areas via random mailings and advertisements, and were screened with the Comprehensive Assessment of Symptoms and History (Comprehensive Assessment of Symptoms and History, CASH) and Family Interview for Genetic Studies to exclude any personal or first–degree family history of psychotic disorder (third and fourth stars).

**20**	Yazici et al. (2019) [87]	Low<br>Observational, cross−sectional design with moderate risk of selection bias and residual confounding (e.g., diet, body mass index, socioeconomic status and medication use not fully accounted for) and no longitudinal follow−up; nonetheless, vitamin D, vitamin B_{12} and folate were objectively quantified using standardized assays, the sample size was moderate and the large odds ratio for vitamin B_{12} deficiency in schizophrenia versus healthy controls, together with biologically plausible mechanisms, provides some upgrading for magnitude of effect, yielding an overall low certainty rating.	3<br>Cases with schizophrenia and substance use disorder were defined using Diagnostic and Statistical Manual of Mental Disorders, Fifth Edition criteria, and healthy controls were drawn from the same institution without major medical or psychiatric comorbidity; vitamin assays were performed in a single hospital laboratory; however, the clinical, hospital−based sampling frame, limited description of how controls were selected and absence of participation−rate data reduce representativeness and result in one star being withheld.	1<br>Age and sex were included as covariates in analysis of covariance models when evaluating vitamin levels, providing partial adjustment for key demographic confounders; there was no multivariable adjustment for diet, smoking, alcohol consumption beyond the diagnostic grouping, physical activity, body mass index, socioeconomic factors or psychotropic medication, leaving substantial residual confounding, and therefore only one comparability star is awarded.	2<br>Exposure assessment for vitamin D, vitamin B_{12} and folate relied on standardized laboratory measurements with clearly defined thresholds for deficiency and insufficiency, and diagnostic grouping into schizophrenia, substance use disorder and healthy controls followed clinical criteria; nonetheless, the retrospective cross−sectional design involved single time−point measurements, no blinding of laboratory personnel explicitly reported and limited detail on completeness and handling of missing vitamin data, so one star is withheld.	6<br>Moderate−quality observational evidence: strengths include standardized biochemical assessment and clearly defined diagnostic categories, whereas limitations relate to hospital−based sampling, cross−sectional design and incomplete control of confounding; these issues warrant caution in causal interpretation but still provide informative estimates of the association between psychiatric diagnoses and vitamin deficiency burden.
**21**	Yee et al. (2016) [88]	Low<br>Evidence is based on a single, modest−sized case−control study (N = 62) with cross−sectional	3<br>Cases were clearly defined as first−episode	2<br>Groups were matched on key demographic variables (age, sex,	2<br>Exposure assessment for vitamin D−related biomarkers (total vitamin D, vitamin	7<br>Overall, this is a moderate−quality observational study:

		measurement of vitamin D status and symptom severity, which precludes firm conclusions about temporality or causality; selection of cases and controls from a single mental health institution introduces potential selection bias and limits external validity. Nonetheless, vitamin D–related biomarkers were measured objectively with validated assays in a blinded laboratory, cases were clinically well characterised, and the observed association between lower bioavailable vitamin D and more severe negative symptoms is biologically plausible and consistent with some prior work, supporting an overall low.	psychosis (first–episode psychosis, FEP) using a structured diagnostic interview (Structured Clinical Interview for DSM–IV Axis I Disorders, SCID–I), and healthy controls without psychiatric history were recruited from the same institution; matching on age, sex and ethnicity was implemented. However, the sampling frame was institutional rather than population–based and participation rates were not fully described, reducing representativeness and resulting in one selection star withheld.	ethnicity) and regression analyses additionally adjusted for gender, ethnicity and duration of untreated psychosis when examining associations between vitamin D measures and symptom severity, providing reasonable control for major confounders of vitamin D status in this setting; although residual confounding by lifestyle or nutritional factors is possible, the matching and adjustment strategy justifies awarding both comparability stars.	D–binding protein, albumin, calcium and calculated bioavailable vitamin D) relied on standardized chemiluminescence immunoassay and enzyme–linked immunosorbent assay procedures in a single laboratory with consistent sample handling, and psychosis status and symptoms were assessed with validated clinical instruments; nonetheless, measurements were taken at a single time point, information on sun exposure and dietary intake was not collected and details on laboratory blinding are limited, so one exposure/outcome star is withheld.	strengths include clearly defined first–episode psychosis (first–episode psychosis, FEP) cases, matched controls, standardized biomarker assessments and appropriate basic adjustment for confounding, whereas limitations include cross–sectional design, institutional sampling and incomplete control for lifestyle and nutritional confounders; the study provides useful but not definitive evidence that lower bioavailable vitamin D is associated with first–episode psychosis and negative symptom severity.
**22**	Yektaş et al. (2019) [89]	Low<br>Evidence arises from a cross–sectional case–control design without longitudinal follow–up, which precludes	3<br>Case groups (attention deficit hyperactivity disorder and autism spectrum	1<br>The authors considered age and gender as covariates in multivariate analyses of	2<br>Exposures (serum vitamin B_{12}, folate and homocysteine) were measured objectively using standardized enzyme–linked	6<br>Overall, this is a moderate–quality observational study: it benefits from

	establishing temporality between micronutrient abnormalities and neurodevelopmental disorders and leaves open the possibility that dietary patterns, selective eating (particularly in autism spectrum disorder), comorbid conditions or other unmeasured confounders explain part of the observed associations. However, diagnoses were based on structured clinical interviews or Diagnostic and Statistical Manual of Mental Disorders, Fifth Edition criteria, laboratory measurements used standardized enzyme−linked immunosorbent assay techniques with reported precision, and the magnitude and consistency of the differences in vitamin B_{12} and homocysteine across groups, especially for autism spectrum disorder, provide a coherent pattern that is consistent with mechanistic hypotheses linking one−carbon metabolism to neurodevelopmental psychopathology; overall, this supports a low (rather than very low) certainty rating under the Grading of Recommendations, Assessment, Development and Evaluation framework.	disorder) were defined using the Schedule for Affective Disorders and Schizophrenia for School−Age Children − Present and Lifetime Version or Diagnostic and Statistical Manual of Mental Disorders, Fifth Edition criteria, and healthy controls were recruited from the same hospital setting with explicit exclusion of chronic medical conditions and psychiatric history; all participants were medication−free and had not received vitamin supplements. Nonetheless, recruitment was confined to a single centre, participation rates were not fully described and controls were not population−based, so representativeness is limited and one	covariance when testing group differences in vitamin B_{12}, folate and homocysteine, which provides partial adjustment for important demographic confounders; however, potentially influential factors such as detailed dietary intake, body mass index, socioeconomic status, physical activity, and, particularly in autism spectrum disorder, restrictive eating patterns were not measured or controlled for, so residual confounding remains substantial and only one comparability star is awarded.	immunosorbent assay−based laboratory methods with reported coefficients of variation, and case/control status and symptom dimensions were assessed with validated diagnostic interviews and rating scales, supporting good internal validity; nevertheless, measurements were based on single time points, it is not clearly stated whether laboratory personnel were blinded to diagnostic group, and some violation of multivariate test assumptions required robust statistics, so one exposure/outcome star is withheld.	structured diagnoses, objective biochemical measurements and an a priori power calculation, but its cross−sectional design, single−centre sampling and incomplete control for lifestyle and nutritional confounders mean that the results should be interpreted as associative rather than causal; the data remain informative for characterising vitamin B_{12} and homocysteine abnormalities in autism spectrum disorder and attention deficit hyperactivity disorder and for generating hypotheses about modifiable nutritional pathways in these conditions.

	selection star is withheld.	

Notes:

- Newcastle−Ottawa Scale (NOS) Criteria: The NOS evaluates the methodological quality of observational studies (cohort and case−control) using a star−based system across three domains, with a maximum of 9 stars:
  - Selection (Max 4 stars): Assesses the representativeness of the exposed/case group, selection of the non−exposed/control group, ascertainment of exposure/definition of cases, and demonstration that the outcome of interest was not present at the start of the study.
  - Comparability (Max 2 stars): Awarded for the comparability of cohorts or cases and controls based on the design or analysis. Typically, one star is given for controlling for the most important factor (e.g., age) and a second for any additional factors.
  - Outcome/Exposure (Max 3 stars): Evaluates the assessment of outcome/exposure, whether follow−up was long enough for outcomes to occur, and the adequacy of follow−up (e.g., <20% loss to follow−up).
  - Quality Classification: Total scores are categorized as High Quality (7−9 stars), Fair/Moderate Quality (5−6 stars), or Low Quality/Poor (≤4 stars).
- GRADE Certainty−of−Evidence Judgments: The GRADE (Grading of Recommendations Assessment, Development and Evaluation) framework assesses the confidence in the estimated effect for each outcome across the body of evidence:
  - Initial Certainty: Evidence from observational studies begins with a Low default rating.
  - Downgrading Factors: The certainty is decreased by one (serious) or two (very serious) levels based on five domains: Risk of Bias (informed by NOS scores), Inconsistency (unexplained heterogeneity), Indirectness (differences in population, intervention, or outcomes), Imprecision (wide confidence intervals), and Publication Bias.
  - Upgrading Factors: Observational evidence may be upgraded for Large Magnitude of Effect, Dose−Response Gradient, or when Residual Confounding would decrease the observed effect.
  - Final Certainty Levels: High: Very confident the true effect is close to the estimate; Moderate: Moderately confident; the true effect is likely close, but may be substantially different; Low: Limited confidence; the true effect may be substantially different; Very Low: Very little confidence in the effect estimate.

Table S7. Comparative random-effects meta-analysis results across nutrient- and comparison-specific evidence streams.

No.	Exposure/ Intervention	k	Pooled OR	95% CI	p (overall)	I^2 (%)	τ^2	Q (df)	p (Q)	Eligible for Phase 2+3 (k≥10)
1	Vitamin D (Supplementation vs Placebo)	17	0.439	(0.272; 0.710)	0.0023	84.2	0.5820	101.30 (16)	< 0.0001	Yes
2	Vitamin D (Status vs Outcome)	13	0.615	(0.424; 0.890)	0.0142	83.3	0.2336	71.73 (12)	< 0.0001	Yes
3	Vitamin B_{12} (Status vs Outcome)	10	0.310	(0.115; 0.834)	0.0253	94.8	1.7957	174.01 (9)	< 0.0001	Yes
4	Vitamin B_{12} (Supplementation vs Placebo)	4	0.989	(0.563; 1.737)	0.9525	23.5	0.0148	3.92 (3)	0.2702	No

Notes:

- Pooled estimates are reported as odds ratios (ORs) with 95% confidence intervals (CIs) under random-effects models. OR < 1 indicates a more favorable psychiatric profile in the higher-status or supplementation group according to the harmonized direction of effect.
- Evidence streams meeting the prespecified k ≥ 10 threshold were eligible for Phase 2 subgroup analyses and Phase 3 meta-regression.
- Vitamin B_{12} supplementation versus placebo/usual care did not meet the k ≥ 10 threshold and is retained as sparse exploratory evidence only.
- Vitamin B_{12} supplementation versus standard care without placebo (k = 1) and vitamin D supplementation versus a non-placebo comparator (k = 1) were not pooled with placebo/usual-care evidence streams.

Table S8. Phase 2 study-level effect estimates used for phenotype-informed exploratory subgroup and age-moderator analyses.

Effect ID	Study	Design	Exposure/ Intervention	Phenotype-informed exploratory subgroup	Age subgroup	OR (95% CI)
1	Ali et al. (2019) [44]	Observational	Vitamin D (Status vs Outcome)	Mixed/Others	Children/Adolescents	1.06 (0.95; 1.18)
2	Altun et al. (2018) [46]	Observational	Vitamin B_{12} (Status vs Outcome)	Mixed/Others	Children/Adolescents	0.0016 (0.0005; 0.0050)
3	Anmella et al. (2025) [47]	Observational	Vitamin B_{12} (Status vs Outcome)	Serotonergic	Children/Adolescents	0.82 (0.72; 0.93)
4	Boerman et al. (2016) [48]	Observational	Vitamin D (Status vs Outcome)	Dopaminergic	Adults	0.5595 (0.3332; 0.9394)
5	Dehbokri et al. (2019) [51]	RCT	Vitamin D (Supplementation vs Placebo)	Dopaminergic	Children/Adolescents	0.58 (0.28; 1.20)
6	Dhiman et al. (2021) [52]	Observational	Vitamin B_{12} (Status vs Outcome)	Serotonergic	Adults	0.514 (0.289; 0.912)
7	Elshorbagy et al. (2018) [53]	RCT	Vitamin D (Supplementation vs Placebo)	Dopaminergic	Children/Adolescents	0.04 (0.01; 0.15)
8	Endres et al. (2016) [54]	Observational	Vitamin D (Status vs Outcome)	Mixed/Others	Adults	0.64 (0.43; 0.95)
9	Erensoy (2020) [55]	Observational	Vitamin B_{12} (Status vs Outcome)	Serotonergic	Adults	0.54 (0.26; 1.12)
10	Esnafoğlu & Yaman (2017) [57]	Observational	Vitamin B_{12} (Status vs Outcome)	Serotonergic	Children/Adolescents	0.16 (0.07; 0.38)
11	Esnafoglu & Ozturan (2020) [56]	Observational	Vitamin B_{12} (Status vs Outcome)	Serotonergic	Children/Adolescents	0.22 (0.11; 0.44)
12	Fabrazzo et al. (2022) [58]	Observational	Vitamin D (Status vs Outcome)	Mixed/Others	Adults	0.20 (0.11; 0.37)
13	Ghaderi et al. (2017) [59]	RCT	Vitamin D (Supplementation vs Placebo)	Mixed/Others	Adults	0.414927 (0.172935; 0.995544)
14	Hemamy et al. (2021) [60]	RCT	Vitamin D (Supplementation vs Placebo)	Mixed/Others	Children/Adolescents	0.30 (0.12; 0.74)
15	Huang et al. (2018) [62]	Observational	Vitamin B_{12} (Status vs Outcome)	Serotonergic	Adults	1.89 (1.15; 3.11)

16	Kaviani et al. (2022) [63]	RCT	Vitamin D (Supplementation vs Placebo)	Serotonergic	Adults	0.32 (0.12; 0.83)
17	Kerley et al. (2017) [64]	RCT	Vitamin D (Supplementation vs Placebo)	Mixed/Others	Children/Adolescents	1.88 (0.59; 6.03)
18	Krivoy et al. (2017) [65]	RCT	Vitamin D (Supplementation vs Placebo)	Dopaminergic	Adults	1.25 (0.44; 3.52)
19	Laird et al. (2023) [66]	Observational	Vitamin B_{12} (Status vs Outcome)	Serotonergic	Adults	1.51 (1.01; 2.27)
20	Libuda et al. (2020) [67]	RCT	Vitamin D (Status vs Outcome)	Serotonergic	Children/Adolescents	1.091833 (0.750599; 1.431331)
21	Madley–Dowd et al. (2022) [68]	Observational	Vitamin D (Status vs Outcome)	Mixed/Others	Children/Adolescents	0.98 (0.90; 1.06)
22	Marsh et al. (2017) [69]	RCT	Vitamin D (Supplementation vs Placebo)	Mixed/Others	Adults	1.904423 (0.474927; 7.636604)
23	Mazahery et al. (2019) [70]	RCT	Vitamin D (Supplementation vs Placebo)	Mixed/Others	Children/Adolescents	0.633075 (0.188537; 2.125762)
24	Mohammadpour et al. (2018) [72]	RCT	Vitamin D (Supplementation vs Placebo)	Mixed/Others	Children/Adolescents	0.283655 (0.104352; 0.771047)
25	Naeini et al. (2019) [73]	RCT	Vitamin D (Supplementation vs Placebo)	Mixed/Others	Children/Adolescents	0.259848 (0.108501; 0.622309)
26	Okasha et al. (2020) [74]	Observational	Vitamin D (Status vs Outcome)	Mixed/Others	Adults	0.215686 (0.047608; 0.977159)
27	Okereke et al. (2020) [75]	RCT	Vitamin D (Supplementation vs Placebo)	Serotonergic	Adults	0.97 (0.87; 1.09)
28	Omidian et al. (2019) [76]	RCT	Vitamin D (Supplementation vs Placebo)	Serotonergic	Adults	0.484945 (0.200239; 1.174457)
29	Petruzzelli et al. (2020) [78]	Observational	Vitamin D (Status vs Outcome)	Mixed/Others	Children/Adolescents	0.096993 (0.018443; 0.510204)
30	Rahman et al. (2023) [79]	RCT	Vitamin D (Supplementation vs Placebo)	Serotonergic	Adults	0.99 (0.90; 1.08)
31	Rouhi et al. (2018) [80]	RCT	Vitamin D (Supplementation vs Placebo)	Serotonergic	Adults	0.147465 (0.063042; 0.344944)
32	Shahini et al. (2022) [81]	Observational	Vitamin D (Status vs Outcome)	Dopaminergic	Adults	0.312500 (0.030791; 3.171569)
33	Sourander et al. (2021) [82]	Observational	Vitamin D (Status vs Outcome)	Mixed/Others	Children/Adolescents	0.72 (0.59; 0.89)
34	Terock et al. (2020) [83]	Observational	Vitamin D (Status vs Outcome)	Mixed/Others	Adults	0.96 (0.93; 0.99)

35	Van der Leeuw et al. (2020) [84]	Observational	Vitamin D (Status vs Outcome)	Dopaminergic	Adults	0.458714 (0.308671; 0.681690)
36	Vaziri et al. (2016) [85]	RCT	Vitamin D (Supplementation vs Placebo)	Serotonergic	Adults	0.249416 (0.132881; 0.468148)
37	Vellekkatt et al. (2020) [86]	RCT	Vitamin D (Supplementation vs Placebo)	Serotonergic	Adults	0.167533 (0.055057; 0.509780)
38	Yazici et al. (2019) [87]	Observational	Vitamin B_{12} (Status vs Outcome)	Dopaminergic	Adults	0.16 (0.08; 0.30)
39	Yee et al. (2016) [88]	Observational	Vitamin D (Status vs Outcome)	Dopaminergic	Adults	0.465056 (0.188679; 1.146268)
40	Yektaş et al. (2019) [89]	Observational	Vitamin B_{12} (Status vs Outcome)	Mixed/Others	Children/Adolescents	0.073365 (0.030426; 0.176904)

Notes: This table lists the single effect per study retained for Phase 2 synthesis and moderator testing. The subgroup variable denotes phenotype-informed exploratory categories used to organize clinical heterogeneity across studies; these categories do not indicate direct measurement of serotonergic, dopaminergic, neuroimmune, astroglial, or metabolic pathway activity. Age subgroup was coded as Adults or Children/Adolescents according to the recruitment frame of each study.

Table S9. Phase 2 random-effects subgroup meta-analyses by phenotype-informed exploratory subgroup.

No.	Exposure_Intervention	Phenotype-informed exploratory subgroup	k	Pooled OR (95% CI)	I^2 (%)	τ^2	τ	Q (df)	p (Q)
1	Vitamin D (Supplementation vs Placebo)	Dopaminergic	3	0.324 (0.004; 26.712)	87.8	2.763	1.662	16.44 (2)	0.0003
2	Vitamin D (Supplementation vs Placebo)	Serotonergic	7	0.406 (0.194; 0.848)	88.7	0.548	0.740	52.99 (6)	<0.0001
3	Vitamin D (Supplementation vs Placebo)	Mixed/Others	7	0.519 (0.241; 1.118)	55.1	0.353	0.594	13.36 (6)	0.0377
4	Vitamin D (Status vs Outcome)	Dopaminergic	4	0.487 (0.399; 0.593)	0.0	0.000	0.002	0.51 (3)	0.9158
5	Vitamin D (Status vs Outcome)	Serotonergic	1	Not pooled (k<3)	–	–	–	–	–
6	Vitamin D (Status vs Outcome)	Mixed/Others	8	0.582 (0.304; 1.114)	86.5	0.405	0.636	51.73 (7)	<0.0001
7	Vitamin B_{12} (Status vs Outcome)	Dopaminergic	1	Not pooled (k<3)	–	–	–	–	–
8	Vitamin B_{12} (Status vs Outcome)	Serotonergic	7	0.610 (0.264; 1.410)	88.4	0.714	0.845	51.53 (6)	<0.0001
9	Vitamin B_{12} (Status vs Outcome)	Mixed/Others	2	Not pooled (k<3)	–	–	–	–	–

Notes: Subgroup strata represent phenotype-informed exploratory categories and should not be interpreted as direct biological pathway measurements. Pooled estimates are reported only for strata with $k \geq 3$. Strata with $k < 3$ were not pooled because such estimates would be statistically unstable. OR < 1 indicates a more favorable psychiatric profile in the higher-status or supplementation group according to the harmonized direction of effect. Heterogeneity is reported using I^2, τ^2, τ, Cochran's Q, and the corresponding Q-test p-value.

Table S10. Phase 2 tests for subgroup differences using phenotype-informed exploratory subgroup as a categorical moderator (Q-between).

Exposure/Intervention	k	Q between	df between	p between	τ^2
Vitamin D (Supplementation vs Placebo)	17	0.519	2	0.771	0.710
Vitamin D (Status vs Outcome)	13	1.774	2	0.412	0.249
Vitamin B_{12} (Status vs Outcome)	10	14.311	2	0.001	1.473

Notes: Q-between tests whether pooled estimates differ across phenotype-informed exploratory subgroup strata (Dopaminergic, Serotonergic, Mixed/Others) within each nutrient- and comparison-specific evidence stream. These tests evaluate statistical heterogeneity across broad study-level clinical categories and do not constitute direct tests of serotonergic or dopaminergic pathway activity. τ^2 is the estimated between-study variance from the corresponding random-effects model.

Table S11. Phase 2 tests for subgroup differences using age subgroup as a categorical moderator (Adults vs. Children/Adolescents; Q-between).

Exposure/Intervention	k	Q between	df between	p between	τ^2
Vitamin D (Supplementation vs Placebo)	17	0.524	1	0.469	0.610
Vitamin D (Status vs Outcome)	13	3.747	1	0.053	0.159
Vitamin B_{12} (Status vs Outcome)	10	3.372	1	0.066	2.961

Notes: Q-between tests whether pooled estimates differ between Adults and Children/Adolescents within each nutrient- and comparison-specific evidence stream. Age subgroup was coded according to the recruitment frame of each study. τ^2 is the estimated between-study variance from the corresponding random-effects model. These analyses are exploratory and should be interpreted in relation to study count, heterogeneity, and confidence-interval width.

Table S12. Phase 3 study-level covariates, phenotype-informed exploratory subgroup assignments, and quality/certainty assessments used for meta-regression and covariate adjustment.

Effect ID	Study	Study design	Exposure/ Intervention	Phenotype-informed exploratory subgroup	RoB2 overall	NOS total score	GRADE certainty	OR	log OR	SE logOR	95%CI	Publication year	Age subgroup
**1**	Ali et al. (2019) [44]	Observational	Vitamin D (Status vs Outcome)	Mixed/ Others	Not applicable	8	Low	1.060	0.058269	0.055308	0.950; 1.180	2019	Children/ Adolescents
**2**	Altun et al. (2018) [46]	Observational	Vitamin B_{12} (Status vs Outcome)	Mixed/ Others	Not applicable	7	Low	0.002	−6.418074	0.574334	0.001; 0.005	2018	Children/ Adolescents
**3**	Anmella et al. (2025) [47]	Observational	Vitamin B_{12} (Status vs Outcome)	Serotonergic	Not applicable	6	Low	0.820	−0.198451	0.065289	0.720; 0.930	2025	Children/ Adolescents

**4**	Boerman et al. (2016) [48]	Observational	Vitamin D (Status vs Outcome)	Dopaminergic	Not applicable	7	Very low	0.560	−0.580716	0.264362	0.333; 0.939	2016	Adults
**5**	Dehbokri et al. (2019) [51]	RCT	Vitamin D (Supplementation vs Placebo)	Dopaminergic	Some concerns	Not applicable	Low	0.580	−0.547746	0.373133	0.280; 1.200	2019	Children/ Adolescents
**6**	Dhiman et al. (2021) [52]	Observational	Vitamin B_{12} (Status vs Outcome)	Serotonergic	Not applicable	9	Low	0.514	−0.665532	0.293167	0.289; 0.912	2021	Adults
**7**	Elshorbagy et al. (2018) [53]	RCT	Vitamin D (Supplementation vs Placebo)	Dopaminergic	Some concerns	Not applicable	Low	0.040	−3.246903	0.686623	0.010; 0.150	2018	Children/ Adolescents
**8**	Endres et al. (2016) [54]	Observational	Vitamin D (Status vs Outcome)	Mixed/Others	Not applicable	5	Very low	0.640	−0.447990	0.201250	0.430; 0.950	2016	Adults
**9**	Erensoy (2020) [55]	Observational	Vitamin B_{12} (Status vs Outcome)	Serotonergic	Not applicable	4	Very low	0.540	−0.608806	0.369220	0.260; 1.120	2020	Adults
**10**	Esnafoğlu & Yaman (2017) [57]	Observational	Vitamin B_{12} (Status vs Outcome)	Serotonergic	Not applicable	6	Low	0.160	−1.843722	0.440653	0.070; 0.380	2017	Children/ Adolescents
**11**	Esnafoglu & Ozturan (2020) [56]	Observational	Vitamin B_{12} (Status vs Outcome)	Serotonergic	Not applicable	6	Low	0.220	−1.508423	0.349606	0.110; 0.440	2020	Children/ Adolescents
**12**	Fabrazzo et al. (2022) [58]	Observational	Vitamin D (Status vs Outcome)	Mixed/ Others	Not applicable	7	Low	0.200	−1.592281	0.308361	0.110; 0.370	2022	Adults

**13**	Ghaderi et al. (2017) [59]	RCT	Vitamin D (Supplementation vs Placebo)	Mixed/ Others	Some concerns	Not applicable	Low	0.415	−0.879653	0.446524	0.173; 0.996	2017	Adults
**14**	Hemamy et al. (2021) [60]	RCT	Vitamin D (Supplementation vs Placebo)	Mixed/ Others	Some concerns	Not applicable	Moderate	0.300	−1.193772	0.458824	0.120; 0.740	2021	Children/ Adolescents
**15**	Huang et al. (2018) [62]	Observational	Vitamin B_{12} (Status vs Outcome)	Serotonergic	Not applicable	7	Low	1.890	0.636577	0.253791	1.150; 3.110	2018	Adults
**16**	Kaviani et al. (2022) [63]	RCT	Vitamin D (Supplementation vs Placebo)	Serotonergic	Some concerns	Not applicable	Moderate	0.320	−1.139434	0.492964	0.120; 0.830	2022	Adults
**17**	Kerley et al. (2017) [64]	RCT	Vitamin D (Supplementation vs Placebo)	Mixed/Others	Some concerns	Not applicable	Moderate	1.880	0.632790	0.593991	0.590; 6.030	2017	Children/ Adolescents
**18**	Krivoy et al. (2017) [65]	RCT	Vitamin D (Supplementation vs Placebo)	Dopaminergic	Some concerns	Not applicable	Moderate	1.250	0.220394	0.529769	0.440; 3.520	2017	Adults
**19**	Laird et al. (2023) [66]	Observational	Vitamin B_{12} (Status vs Outcome)	Serotonergic	Not applicable	8	Low	1.510	0.412110	0.206589	1.010; 2.270	2023	Adults
**20**	Libuda et al. (2020) [67]	RCT	Vitamin D (Status vs Outcome)	Serotonergic	Some concerns	Not applicable	Low	1.092	0.087858	0.164666	0.751; 1.431	2020	Children/ Adolescents
**21**	Madley–Dowd et al. (2022) [68]	Observational	Vitamin D (Status vs Outcome)	Mixed/ Others	Not applicable	8	Low	0.980	−0.020203	0.041742	0.900; 1.060	2022	Children/ Adolescents

**22**	Marsh et al. (2017) [69]	RCT	Vitamin D (Supplementation vs Placebo)	Mixed/ Others	Some concerns	Not applicable	Low	1.904	0.644179	0.708558	0.475; 7.637	2017	Adults
**23**	Mazahery et al. (2019) [70]	RCT	Vitamin D (Supplementation vs Placebo)	Mixed/ Others	High	Not applicable	Low	0.633	−0.457166	0.618008	0.189; 2.126	2019	Children/ Adolescents
**24**	Mohammadpour et al. (2018) [72]	RCT	Vitamin D (Supplementation vs Placebo)	Mixed/ Others	Some concerns	Not applicable	Moderate	0.284	−1.259997	0.510200	0.104; 0.771	2016	Children/ Adolescents
**25**	Naeini et al. (2019) [73]	RCT	Vitamin D (Supplementation vs Placebo)	Mixed/ Others	Some concerns	Not applicable	Moderate	0.260	−1.347657	0.445581	0.109; 0.622	2018	Children/ Adolescents
**26**	Okasha et al. (2020) [74]	Observational	Vitamin D (Status vs Outcome)	Mixed/ Others	Not applicable	4	Low	0.216	−1.533930	0.770829	0.048; 0.977	2020	Adults
**27**	Okereke et al. (2020) [75]	RCT	Vitamin D (Supplementation vs Placebo)	Serotonergic	Low	Not applicable	Moderate	0.970	−0.030459	0.057510	0.870; 1.090	2020	Adults
**28**	Omidian et al. (2019) [76]	RCT	Vitamin D (Supplementation vs Placebo)	Serotonergic	Some concerns	Not applicable	Moderate	0.485	−0.723719	0.451288	0.200; 1.174	2019	Adults
**29**	Petruzzelli et al. (2020) [78]	Observational	Vitamin D (Status vs Outcome)	Mixed/ Others	Not applicable	6	Very low	0.097	−2.333114	0.846966	0.018; 0.510	2020	Children/ Adolescents

**30**	Rahman et al. (2023) [79]	RCT	Vitamin D (Supplementation vs Placebo)	Serotonergic	Low	Not applicable	High	0.990	−0.010050	0.046511	0.900; 1.080	2023	Adults
**31**	Rouhi et al. (2018) [80]	RCT	Vitamin D (Supplementation vs Placebo)	Serotonergic	Some concerns	Not applicable	Low	0.147	−1.914167	0.433568	0.063; 0.345	2018	Adults
**32**	Shahini et al. (2022) [81]	Observational	Vitamin D (Status vs Outcome)	Dopaminergic	Not applicable	6	Very low	0.312	−1.163151	1.182335	0.031; 3.172	2022	Adults
**33**	Sourander et al. (2021) [82]	Observational	Vitamin D (Status vs Outcome)	Mixed/ Others	Not applicable	9	Moderate	0.720	−0.328504	0.104872	0.590; 0.890	2021	Children/ Adolescents
**34**	Terock et al. (2020) [83]	Observational	Vitamin D (Status vs Outcome)	Mixed/ Others	Not applicable	8	Very low	0.960	−0.040822	0.015949	0.930; 0.990	2020	Adults
**35**	Van der Leeuw et al. (2020) [84]	Observational	Vitamin D (Status vs Outcome)	Dopaminergic	Not applicable	9	Very low	0.459	−0.779329	0.202117	0.309; 0.682	2020	Adults
**36**	Vaziri et al. (2016) [85]	RCT	Vitamin D (Supplementation vs Placebo)	Serotonergic	Some concerns	Not applicable	Moderate	0.249	−1.388635	0.321257	0.133; 0.468	2016	Adults
**37**	Vellekkatt et al. (2020) [86]	RCT	Vitamin D (Supplementation vs Placebo)	Serotonergic	Some concerns	Not applicable	Moderate	0.168	−1.786577	0.567756	0.055; 0.510	2020	Adults
**38**	Yazici et al. (2019) [87]	Observational	Vitamin B_{12} (Status vs Outcome)	Dopaminergic	Not applicable	6	Low	0.160	−1.856500	0.339911	0.080; 0.300	2019	Adults

**39**	Yee et al. (2016) [88]	Observational	Vitamin D (Status vs Outcome)	Dopaminergic	Not applicable	7	Low	0.465	−0.765598	0.460260	0.189; 1.146	2016	Adults
**40**	Yektaş et al. (2019) [89]	Observational	Vitamin B_{12} (Status vs Outcome)	Mixed/ Others	Not applicable	6	Low	0.073	−2.612303	0.449059	0.030; 0.177	2019	Children/ Adolescents

Notes: This table reports the study-level covariates and quality/certainty assessments used for Phase 3 meta-regression and covariate screening. The subgroup variable denotes phenotype-informed exploratory categories and should not be interpreted as direct measurement of serotonergic or dopaminergic pathway activity. RoB 2 overall judgments apply to randomized trials; NOS total score applies to observational studies; GRADE certainty reflects the certainty-of-evidence judgment assigned to the corresponding estimate. "Not applicable" indicates that the instrument was not used for that study design. logOR and SE_logOR are reported on the natural log scale.

Table S13. Phase 3 meta-regression and covariate-adjustment summary for phenotype-informed exploratory subgroup, age, and study-level covariates.

**Exposure /Intervention**	**k**	**Phenotype-informed subgroup moderator (QM)**	**Subgroup df**	**Subgroup p**	**Age moderator (QM)**	**Age df**	**Age p (QM)**	**Age beta (Children vs Adults)**	**Age p (HK)**	**Year p (HK)**	**RoB2 ord p (HK)**	**GRADE p (HK)**	**NOS total score p (HK)**
Vitamin D (Supplementation vs Placebo)	17	0.519	2	0.771	0.524	1	0.469	−0.328	0.499	0.706	0.265	0.287	Not applicable
Vitamin D (Status vs Outcome)	13	1.774	2	0.412	3.747	1	0.053	0.528	0.110	0.871	Not applicable	0.601	0.161
Vitamin B_{12} (Status vs Outcome)	10	14.311	2	0.001	3.372	1	0.066	−2.041	0.107	0.171	Not applicable	0.692	0.843

*Notes: Omnibus tests are reported for phenotype-informed exploratory subgroup and age moderators; covariate p-values reflect the Phase 3 inference approach implemented for meta-regression. The phenotype-informed subgroup moderator tests whether effect estimates differ across Dopaminergic, Serotonergic, and Mixed/Others study-level exploratory categories; it does not test directly measured pathway activity. The age moderator tests differences between Adults and Children/Adolescents. Age beta (Children

vs Adults) is the estimated coefficient on the log(OR) scale; positive values indicate higher log(OR) in Children/Adolescents relative to Adults. "HK" indicates Hartung–Knapp-type inference for p-values where applicable. "Not applicable" indicates not estimated or not applicable in the corresponding model.

Table S14. SMD-based interpretability analysis for supplementation evidence streams.

Evidence family	k	Primary pooled OR (95% CI)	Pooled SMD (Hedges' g) (95% CI)	I^2 (%)	τ^2 (SMD scale)
Vitamin D (Supplementation vs Placebo/usual care)	17	0.439 (0.272–0.710)	-0.454 (-0.718–-0.189)	84.2	0.1769
Vitamin B_{12} (Supplementation vs Placebo/usual care)	4	0.989 (0.563–1.737)	-0.006 (-0.317–0.304)	23.5	0.0045

Notes: This table provides SMD-based interpretation for supplementation evidence streams, where psychiatric outcomes were frequently reported as continuous symptom-rating scales. SMD values were obtained by back-transforming pooled log(OR) estimates using the inverse logistic approximation $SMD = (\sqrt{3}/\pi) \times \log(OR)$. Negative SMD values indicate more favorable psychiatric outcomes in the supplementation group according to the harmonized direction of effect. For supplementation evidence streams, SMD estimates should be regarded as the primary metric for clinical interpretability, whereas OR estimates are retained as harmonized cross-family metrics. τ^2 on the SMD scale was computed as $\tau^2\_SMD = (\sqrt{3}/\pi)^2 \times \tau^2\_\log(OR)$. The vitamin B_{12} supplementation evidence stream included only four placebo-controlled randomized comparisons and should be interpreted as sparse exploratory evidence.

Exploratory Supplementary Analysis: Vitamin B_{12} Supplementation versus Placebo/Usual Care

The placebo-controlled vitamin B_{12} supplementation evidence family included four randomized comparisons and did not meet the prespecified $k \geq 10$ threshold for primary pooled inference, Phase 2 subgroup analysis, Phase 3 meta-regression, or small-study-effect assessment. Therefore, these results are presented as exploratory supplementary evidence. The pooled OR was null (OR = 0.989, 95% CI 0.563–1.737; p = 0.9525), and the corresponding pooled SMD was essentially zero (SMD = −0.006, 95% CI −0.317 to 0.304). These findings do not support routine vitamin B_{12} supplementation across unselected psychiatric populations, while the sparse evidence base prevents strong inference for deficiency-enriched subgroups.

Table S15. Exploratory study-level effect estimates for vitamin B12 supplementation versus placebo/usual care.

No.	Study	logOR	SE	Weight (%)	OR (95% CI)
1	Hendren et al. (2016) [61]	−1.1082	0.6094	6.80	0.330 (0.100; 1.090)
2	Allott et al. (2019) [45]	−0.0869	0.3745	16.90	0.917 (0.440; 1.910)
3	Chen et al. (2024, Taiwan) [49]	−0.0161	0.5131	9.40	0.984 (0.360; 2.690)
4	De Koning et al. (2016) [50]	0.1195	0.1516	66.90	1.127 (0.830; 1.530)
	Random effects model (IV, KH−adjusted)			100.00	0.989 (0.563; 1.737)
	Prediction Interval (PI)				(0.519; 1.883)

Notes: This table reports the four placebo-controlled randomized comparisons contributing to the exploratory vitamin B12 supplementation evidence family. logOR denotes the natural logarithm of the odds ratio, SE denotes the standard error of logOR, and OR denotes the odds ratio after back-transformation. OR < 1 indicates a more favorable psychiatric profile in the supplementation group according to the harmonized direction of effect. Because k = 4, these estimates were not eligible for Phase 2 subgroup analysis, Phase 3 meta-regression, or small-study-effect assessment.

Table S16. Exploratory random-effects meta-analysis of vitamin B12 supplementation versus placebo/usual care.

Domain	Statistic	Value
Pooled effect	Number of studies (*k*)	4
	Odds Ratio (OR, 95% CI)	0.989 (0.563; 1.737)
	LogOR (SE)	−0.011 (0.187)
	p−value (overall effect)	0.9525
Heterogeneity	Cochran's Q test (Chi^2)	3.92

Domain	Statistic	Value
	Degrees of freedom (df)	3
	p−value (Q test)	0.2702
	I^2 (95% CI), %	23.5 (0; 88.3)
	τ^2 (95% CI)	0.0148 (0; 4.115)
	τ (95% CI)	0.12 (0; 2.028)
	Higgins' H statistic (95% CI)	1.14 (1; 2.921)
Prediction interval (PI)	95% PI (OR scale)	0.519 to 1.883

Notes: OR (Odds Ratio) represents the relative odds of the psychiatric outcome in the vitamin B12 supplementation group compared with placebo/usual care under the harmonized direction of effect. This evidence family included only four randomized comparisons and did not meet the prespecified threshold for primary pooled inference or moderator analysis. The pooled OR was null, and the corresponding SMD estimate reported in Table S14 was also essentially zero. These results should be interpreted as sparse exploratory evidence rather than as a stable estimate of supplementation efficacy.
